# Hybrid machine learning driven optimization of multilayer SPR sensor for high sensitivity milk fat detection

**DOI:** 10.1371/journal.pone.0355996

**Published:** 2026-09-02

**Authors:** Md. Al Amin Islam Utshob, Maymona Binte Juwel, Nahyan Al Mahmud, S. M. Ishraqul Huq, Khandakar Mohammad Ishtiak

**Affiliations:** 1 Department of Electrical & Electronic Engineering, Ahsanullah University of Science and Technology, Dhaka, Bangladesh; 2 Department of Electrical and Computer Engineering, McGill University, Montreal, Quebec, Canada; Beni-Suef University, EGYPT

## Abstract

Accurate determination of fat content in milk is crucial for ensuring the quality, nutritional value, and economic grading of dairy products. In this study, a highly sensitive multilayer surface plasmon resonance biosensor is proposed for fat content detection in milk samples using the angular interrogation method at a fixed wavelength of 633 nm. The proposed sensor is designed based on a Kretschmann configuration and includes a SiO_2_ prism and optimized multilayer films of MgO, Ag, BaTiO_3_, and BP. The combination of high refractive index dielectric materials like MgO and BaTiO_3_ with ultrathin BP plays a vital role in enhancing the performance of SPR biosensors. To ensure accuracy in the SPR biosensor design, the performance of the SPR biosensor has been rigorously analyzed using the transfer matrix method, finite element method, and finite difference time-domain method. Moreover, the parameter optimization process is performed by a hybrid technique that uses the brute-force method, ML, and refinement processes, making it possible to determine the optimal thickness of the layers for the highest sensitivity. The proposed sensor has a refractive index range of 1.345 to 1.3621, which relates to the fat content in the milk product. When the refractive index of the sensor was set at 1.3621, the sensitivity was found to be high at 401.40 deg/RIU, the minimum reflectance was 0.086, the quality factor was 148.33 RIU^-1^, and the accuracy was 0.369 deg^-1^. This study has made a promising numerical SPR sensing technique in the development of conventional SPR biosensors and has the potential to be used in the food industry for the monitoring of milk quality and adulteration.

## 1. Introduction

Milk is one of the most essential components of human nutrition, and it is widely regarded as one of the most commonly used food items in the world. Milk is very valuable in terms of its nutritional content, including essential nutrients such as proteins, carbohydrates, vitamins, and minerals, including salts and calcium, all of which are very important for overall well-being [1]. Milk is a very valuable nutritional component, and it is easily accessible and affordable, thus making it one of the commonly used food items in daily life, especially in developing regions of the world. Because of its cost-effectiveness, even in developing regions, people in poor economic conditions are able to afford it for fulfilling their nutritional demands [2]. In developing regions, a large portion of the dairy market is occupied by small-scale vendors, thus proving its economic importance. Milk is one of the most commonly used nutritional components, and it is widely recommended by nutritionists for daily consumption in combination with dairy products such as yogurt and cheese, mainly for fulfilling nutritional demands, especially for calcium, thus developing strong bones and teeth [3,4]. Although water constitutes approximately 87% of milk’s composition, its nutritional values are obtained from the remaining 13% [5]. In this context, fat content is one of the vital quality parameters that affects both nutritional values and monetary values. Thus, it is critical to measure fat content in terms of percentage in the dairy industry. Additionally, milk adulteration has emerged as a serious food safety issue globally, especially in developing nations where there are economic cheating and lack of quality control [6,7]. Adulterations involve diluting milk with water, skimmed from milk fat, and adding inexpensive components such as starch, urea, detergents, and preservatives to make more profits [8]. This results in both reduced nutritional value of milk and health hazards. Therefore, fast and effective methods for analyzing milk quality, especially its fat content, have become crucial.

Milk fat content can be measured using traditional techniques such as the Gerber Test, Babcock Test, and Fourier Transform Infrared Spectroscopy [9–11]. These traditional techniques have been used for decades. However, they are associated with the use of chemical reagents and are time-consuming. Moreover, they are not suitable for real-time detection. In addition, they might not produce precise results when dealing with complex samples. These limitations have raised the need for rapid detection techniques. In the above-mentioned context, surface plasmon resonance-based sensors have been identified as a promising alternative for the analysis of fat in milk samples [12]. SPR-based sensors are considered suitable for precise measurements owing to their label-free analysis, quick response, and high sensitivity characteristics [13,14]. The working mechanism of the SPR-based sensors involves the detection of changes in the refractive index of the samples, which is directly related to the fat percentage in the milk samples. Such a technique facilitates the real-time analysis of the samples without the involvement of chemicals or extensive processing steps [15]. Such efficiency, accuracy, and ease of operation of the SPR-based sensors make it a much better alternative for the analysis of fat in the milk samples [16].

Surface plasmon resonance is an optical phenomenon that occurs when there is an interface between a metal and a dielectric [17]. When this interface is exposed to light under specific conditions, it is able to couple with free electrons in the metal and cause oscillation. This oscillation is known as a surface plasmon polariton [18]. This coupling occurs when the momentum of the light is equivalent to that of the plasmons. This is usually done using a prism coupling technique. When this occurs, a sharp drop in reflectivity is observed at a specific angle known as the resonance angle [19]. The principle of SPR is based on changes in refractive index [20]. When an analyte is introduced to a sensor that is based on SPR, any changes in refractive index cause changes in the propagation of plasmons. This is manifested as a shift in the resonance angle. The dip is caused by transfer of energy from photons to plasmons. It is due to high sensitivity of surface plasmon resonance (SPR) to refractive index changes [21]. In this study, a highly sensitive SPR sensor based on a sensitive technique known as angular interrogation is proposed by employing a modified Kretschmann configuration [22]. The Kretschmann configuration has become the most commonly used SPR excitation configuration because of its simplicity, efficiency of the excitation process, and good optical coupling between the light source and the metal-dielectric interface. In comparison to other SPR excitation configurations, it has high measurement stability, good feasibility, and high sensitivity, which makes it appropriate for measuring refractive indices [23]. This configuration can be applied as a platform for the real-time detection of milk fat content. The structure of the proposed SPR sensor is a multi-layer consisting of a SiO_2_ prism, a MgO layer, a silver layer, a BaTiO_3_ layer, and a black phosphorus layer. The SiO_2_ prism is used in this study owing to its high transparency and high refractive index, thus allowing for high efficiency in the coupling of incident light with surface plasmons [24]. A thin layer of MgO is used to improve the efficiency of light confinement and thus increase the efficiency of coupling incident light with surface plasmons [25]. Silver, a metal, is used in this study owing to its high efficiency in supporting surface plasmons. Silver has a high ability to support SPR and thus is used in this study [26]. Furthermore, a thin layer of BaTiO_3_ is used in this study owing to its high refractive index and dielectric constants, thus enhancing the efficiency of electromagnetic field localization. Finally, an ultrathin layer of black phosphorus (BP) is deposited on top as the sensing medium. BP is a two-dimensional material that offers excellent optical and electronic properties [27,28]. These properties include high carrier mobility, bandgap tunability, and excellent light-matter interactions [29]. This ensures better absorbability of the analyte molecules and better sensitivity using the field confinement effect [30]. Additionally, the structural parameters of the designed multilayer SPR sensor are optimized using a hybrid optimization technique that incorporates brute-force algorithms for optimization, and iterative optimization. This optimization technique ensures that the optimal thickness values of the layers are obtained, resulting in better sensitivity for refractive index variations of the analyte molecules [31]. The detection principle behind the suggested SPR sensor is based on angular interrogation. The alterations in the milk’s components composition, such as alterations in the fat component or in its adulteration, alter the effective refractive index of the medium, as shown by experimental studies [32]. Such alterations in the effective refractive index change the momentum matching required for exciting surface plasmons in the metal-analyte interface, thereby causing an alteration in the SPR resonance angle. Consequently, the suggested sensor indirectly senses any alterations in milk’s components composition through the alteration in its refractive index. However, it should be noted that current analysis uses the refractive index values obtained from experimentally performed studies for numerical evaluation, whereas the direct calibration of fat concentration and SPR response will be done by using the actual milk samples in future experiments. In future experiments, the sensitivity of the detection system may be improved by functionalizing the biosensor surface using biorecognition layers like lipase, PAA-Chitosan-Lipase, or PMMA-Lipase. Lipase enzyme acts on the triglycerides in milk fat and cleaves them into glycerol and fatty acids. In addition to generating the biochemical reaction, there is a corresponding change in the local refractive index, which causes a shift in SPR resonance, thus enabling selective detection of milk fat [33]. Immobilization of enzymes using polymer assistance, such as in PAA-Chitosan or PMMA matrices, enhances the enzyme immobilization, reusability, and selectivity of biosensor systems [34,35]. The recent SPR studies conducted with regard to the sensing of the milk fat quality have shown some promising results when using different combinations of materials and structural arrangements. Nevertheless, the study presented in [36] was mostly concerned with the biosensing performance without looking into structural optimization in detail. At the same time, the study in [37] focused on the development of increased sensitivity due to the use of material engineering. The same can be noted about the works presented in [38,39]. However, no mathematical model for the fast sensor design was built in the course of these experiments. In its turn, this work uses the approach of multilayer structure optimization together with the predictive MLR model for fast performance estimation.

In this research, a systematically optimized multi-layer SiO_2_/MgO/Ag/BaTiO_3_/BP SPR biosensor is developed for precise detection of fat content in milk products by addressing the need for efficient and timely quality evaluation of dairy products. A unique contribution-focused perspective is provided to emphasize the novelty of this research. In this research, a multi-layer SPR biosensor is developed by utilizing the combined effect of high refractive index dielectric materials MgO and BaTiO_3_ and a two-dimensional black phosphorus (BP) material. These materials are selected for their high refractive index and their potential to improve the performance of SPR biosensors. To ensure the precision and reliability of the performance of this biosensor, a comprehensive analysis is carried out using a transfer matrix method (TMM), finite element method (FEM), and a finite difference time-domain (FDTD) method. In addition to this, structural parameters are optimized using a hybrid optimization approach that combines brute force and iterative optimization techniques to identify the optimal structural configurations for maximum sensitivity and detection precision. Unlike conventional SPR studies that concentrate on the basic structural tuning, the present study has put more emphasis on the robust and systematic optimization technique, which has significantly improved the overall sensing characteristics. Although the present study has followed a simulation-based approach, the design has the potential to be used in conjunction with conventional SPR setups in the real-time monitoring of milk quality and adulteration.

## 2. Design methodology

### 2.1 Modeling framework and simulation strategy

The proposed multilayer SPR sensor was analyzed using a holistic approach involving analytical modeling, numerical analysis, and artificial intelligence. Initially, the optical response of the proposed SPR sensor was analyzed using the transfer matrix method (TMM) with the help of the MATLAB programming environment. In this regard, p-polarized light of wavelength 633 nm was used [40], and the angle of incidence was varied systematically from 60 deg to 89.99 deg to determine the resonance angle and calculate the angular sensitivity of the proposed SPR sensor. In order to achieve optimal performance, a brute-force optimization approach was used to optimize the thickness of each layer of the multilayer SPR sensor.

In order to further authenticate and endorse the results obtained for the accuracy of the analysis, a full-wave electromagnetic simulation was performed using the Finite Element Method (FEM) in COMSOL Multiphysics software package. The simulation parameters were chosen to be at a 633nm wavelength, and the angle of incidence ranged from 70 deg to 89.9 deg to observe the resonance effect. An extremely fine mesh was created using physics-controlled meshing for precise spatial resolution, including mapped meshing for the ultrathin films and tetrahedral meshing for the sensing region. The mesh size for this simulation was chosen to be 0.2nm, ensuring precise simulation results for the evanescent electromagnetic fields near the interfaces. The results of the simulation were obtained using the solver PARDISO with a relative tolerance of 1 × 10^−6^, and the convergence of the results was verified by refining the mesh and ensuring that the changes in the results were less than 0.1%. The results of the FEM analysis clearly validate the excitation of the surface plasmons, as indicated by the detailed 2D and 3D plots of the electric field, especially the strong Z-component of the electric field observed at the resonance angle of 80.11 deg.

Further electromagnetic simulation calculations were performed using the Finite Difference Time Domain (FDTD) method to assess and authenticate the reliability and precision of the results obtained. The simulation area was established at 500 nm × 500 nm in-plane dimensions, and a grid size of 0.1 nm or less was allowed to ensure precise simulation results for the ultrathin multilayer structure. Moreover, a strict convergence criterion of 1 × 10^−6^ was enforced to guarantee numerical stability and precision. The refractive index (RI) of the medium was varied from 1.3450 to 1.3621 to cover various aqueous environments [41]. The simulated results for resonance characteristics and sensitivity showed outstanding agreement and consistency with those calculated from TMM and FEM results, thus proving the reliability and precision of the proposed sensor.

### 2.2 Structural modelling

The suggested SPR sensor is composed of a multilayer structure that includes SiO_2_, MgO, Ag, BaTiO_3_, BP, and the sensing medium. In this sensor structure, the Kretschmann configuration is used to generate SPs using the prism coupling technique. This configuration is commonly used because of its straightforward alignment technique and precise control over optical parameters. Therefore, this configuration is highly appropriate for using SPR sensors. In this sensor structure, a SiO_2_ prism with RI = 1.457 is used as a coupling prism [24]. This prism is used to allow the incident light to couple with the metal layer at a constant wavelength of λ = 633 nm. A thin MgO layer with RI = 1.7346 is used between the prism and the plasmonic metal layer [42]. This layer is used as an adhesive layer. This layer is used to enhance the bonding between the prism and the metal layer. This layer is also used to enhance the electromagnetic field [43]. This makes the resonance dip sharper, the sensitivity stronger, and the damping losses in the Ag layer lower.

The complex RI of Ag, utilized as the plasmonic layer in the proposed SPR structure, is calculated using the Drude-Lorentz model for the working wavelength *λ*. This is represented by Equation (1), where *λₚ* and *λ*_*c*_ represent the plasmon wavelength and collision wavelength of silver, respectively. In this context, *λₚ* = 1.7614 × 10^−5^ m and *λ*_*c*_ = 1.4541 × 10^−7^ m [44,45],


$$\begin{array}{c} n_{Ag}=\;{\left(1-\frac{\lambda_{c}\times \lambda^{2}}{{\lambda_{p}^{2}(\lambda }_{c}+i\lambda )}\right)}^{\frac{1}{2}} \end{array}$$ (1)

**Fig 1:**
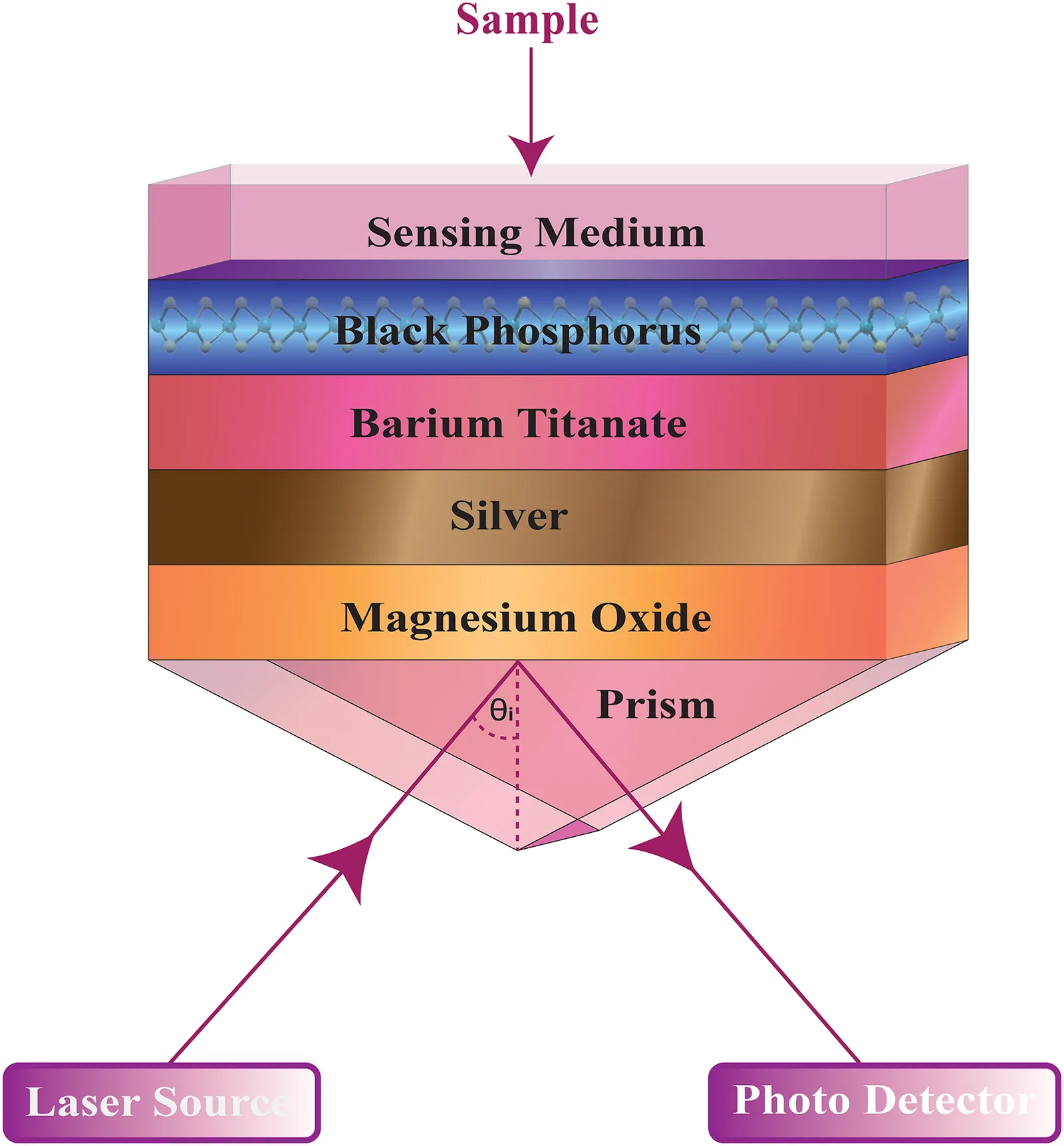
The schematic diagram of proposed multilayer SPR biosensor.

The inclusion of the BaTiO_3_ layer is based on its high dielectric constant, good chemical stability, and durability in different conditions [46]. Here, it is assumed that the RI of the BaTiO_3_ is equal to 2.4042 [47]. This value is very high, so it improves the localization of the electromagnetic wave at the interface of the metal and dielectric, thus increasing the sensitivity of the SPR sensor by enhancing its response to small changes in the RI of the analyte medium. An ultrathin layer of black phosphorus is included at the top of the structure. Its refractive index is given by 3.5 + 0.01i [44]. It must be emphasized that the selected BP thickness value (0.53 nm) equals the experimental value of the thickness of a monolayer of black phosphorus It is a material characteristic; thus, the optimal thickness cannot be obtained [48,49]. Black phosphorus is known for its excellent optoelectronic properties, including high carrier mobility and strong interaction with light [50]. These characteristics improve the sensitivity of the SPR sensor by increasing its response even for small changes in the refractive index of the analyte. Its smooth surface is very suitable for the adsorption of the analyte. Table 1 presents a summary of all the layers used in this proposed SPR configuration, including their respective refractive indices and thicknesses. In this study, it is assumed that a change in the concentration of milk fat is used as a parameter for analysis, thus causing a change in the RI of the analyte. These changes in the RI will, in turn, cause a change in the propagation of the surface plasmons on the interface of the metal and dielectric. Thus, a change in the resonance angle is observed. The range of this change is determined by the change in RI for different concentrations of fat in milk. Fig. 1 presents a schematic representation of this proposed multilayer SPR sensor.

**Table 1:** Thickness and RI values of the sensor materials at wavelength 633 nm.

Materials	Thickness (nm)	RI	References
SiO_2_	–	1.457	[24]
MgO	18	1.7346	[42]
Ag	50	0.031259 + 4.3902i	[51]
BaTiO_3_	3	2.4042	[47]
BP	0.53	3.5 + 0.01i	[44]

### 2.3 Mathematical modelling

Prism Coupling is one of the most frequently used techniques of SPs excitation in the Kretschmann configuration. This technique enables precise control of experimental conditions and parameters. In addition, it is relatively easy to align [52]. In this configuration, the resonant SPs excitation is achieved by the interaction of a transversely magnetic polarized light beam and the interface of a metal and a dielectric material at a certain angle of incidence and wavelength. The resonance condition is achieved when the propagation constant of the incident light (*K*_*light*_) matches the wave vector of SPs (*β*_*SP*_). Therefore, the prism coupling configuration can be written as [53]:


$$\begin{array}{c} K_{light}=\beta_{SP} \end{array}$$ (2)

$$\begin{array}{c} \frac{\text{2}\pi }{\lambda }n_{p}sin\left(\theta_{i}\right)=\frac{\text{2}\pi }{\lambda }Re\left\{\sqrt{\frac{\varepsilon_{m}\varepsilon_{d}}{\varepsilon_{m}{+\varepsilon }_{d}}}\right\} \end{array}$$ (3)

Here, *n*_*p*_ and *λ* denote the RI of the prism and the wavelength of the incident light, respectively. *θ*_*i*_ denotes the angle of incidence, while *ε*_*m*_, *ε*_*d*_ represents the real part of the dielectric parameter associated with the metal. The angle of incidence for SPR can be determined using the following equation [54]:


$$\begin{array}{c} \theta_{i}={sin}^{-\text{1}}\left[\left(\frac{\text{1}}{n_{p}}\right)Re\left\{\sqrt{\frac{\varepsilon_{m}\varepsilon_{d}}{\varepsilon_{m}{+\varepsilon }_{d}}}\right\}\right] \end{array}$$ (4)

Angular modulation is utilized to examine the performance of the proposed multilayer structure. The reflectance of p-polarized incident light is computed by the Transfer Matrix Method, as per the Fresnel reflection theory of multilayer structures, to examine the optical properties of the proposed structure [55]. The characteristic surface plasmon resonance curve is achieved by plotting the total reflected intensity (*R*_*p*_) as a function of the incidence angle (*θ*). The equation describing the total reflectivity (*R*_*p*_) and the reflection coefficient (*q*_*p*_) are as follows [56]:


$$\begin{array}{c} R_{p}=\;q_{p}q_{p}^{*}\;=\;{\left|q_{p}\right|}^{\text{2}} \end{array}$$ (5)

$$\begin{array}{c} q_{p}=\frac{\left(M_{\text{1}\text{1}}+M_{\text{1}\text{2}}n_{N}\right)-\left(M_{\text{2}\text{1}}+M_{\text{2}\text{2}}n_{N}\right)}{\left(M_{\text{1}\text{1}}+M_{\text{1}\text{2}}n_{N}\right)+\left(M_{\text{2}\text{1}}+M_{\text{2}\text{2}}n_{N}\right)} \end{array}$$ (6)

Here, M denotes the characteristics matrix associated with the SPR sensor [57]. The transverse RI and attributes matrix are delineated as follows [58],


$$\begin{array}{c} n_{k}=\sqrt{\left([\begin{array}{c} \mu_{k}\\ \varepsilon_{k} \end{array}]\right)}cos\theta_{k}=\sqrt{\frac{\varepsilon_{k}-{\left(n_{p}sin\theta \right)}^{\text{2}}}{\varepsilon_{k}^{\text{2}}}} \end{array}$$ (7)

$$\begin{array}{c} M_{if}={\left[\prod_{K=\text{2}}^{N-\text{1}}(\begin{array}{cc} \text{cos}\beta_{k} & -isin\;\beta_{k}\\ -in_{k}sin\;\beta_{k} & \text{cos}\beta_{k} \end{array})\right]}_{if}=[\begin{array}{cc} M_{\text{1}\text{1}} & M_{\text{1}\text{2}}\\ M_{\text{2}\text{1}} & M_{\text{2}\text{2}} \end{array}] \end{array}$$ (8)

Furthermore, the equations determine the propagation constant (*β*_*k*_) and wave impedance (*z*_*k*_) associated with the *k*^*th*^ layer [59],


$$\begin{array}{c} \beta_{k}=\frac{\text{2}\pi }{\lambda }n_{k}cos\theta_{k}\left(z_{k}-z_{k-\text{1}}\right)=\frac{\text{2}\pi }{\lambda }d_{k}\sqrt{\varepsilon_{k}-{\left(n_{p}sin\theta \right)}^{\text{2}}\;} \end{array}$$ (9)

$$\begin{array}{c} z_{k}=\frac{k_{x}n_{k}cos\theta_{k}}{\left(\frac{\text{2}\pi c}{\lambda_{inc}}\right)\varepsilon_{k}^{\text{2}}} \end{array}$$ (10)

Where, *d*_*k*_ and *ε*_*k*_ are the thickness and permittivity associated with *k*^*th*^ stratum, respectively. And for the *k*^*th*^ stratum, polar input angle *θ*_*k*_ is given by [56]:


$$\begin{array}{c} \theta_{k}=\frac{\text{1}}{cos\left(\sqrt{\text{1}-\left(\frac{n_{k-\text{1}}}{n_{k}}\right)(sin\theta {)}^{\text{2}}}\right)} \end{array}$$ (11)

### 2.4 Optical response and material compatibility characterization of the SPR sensor

**Fig 2:**
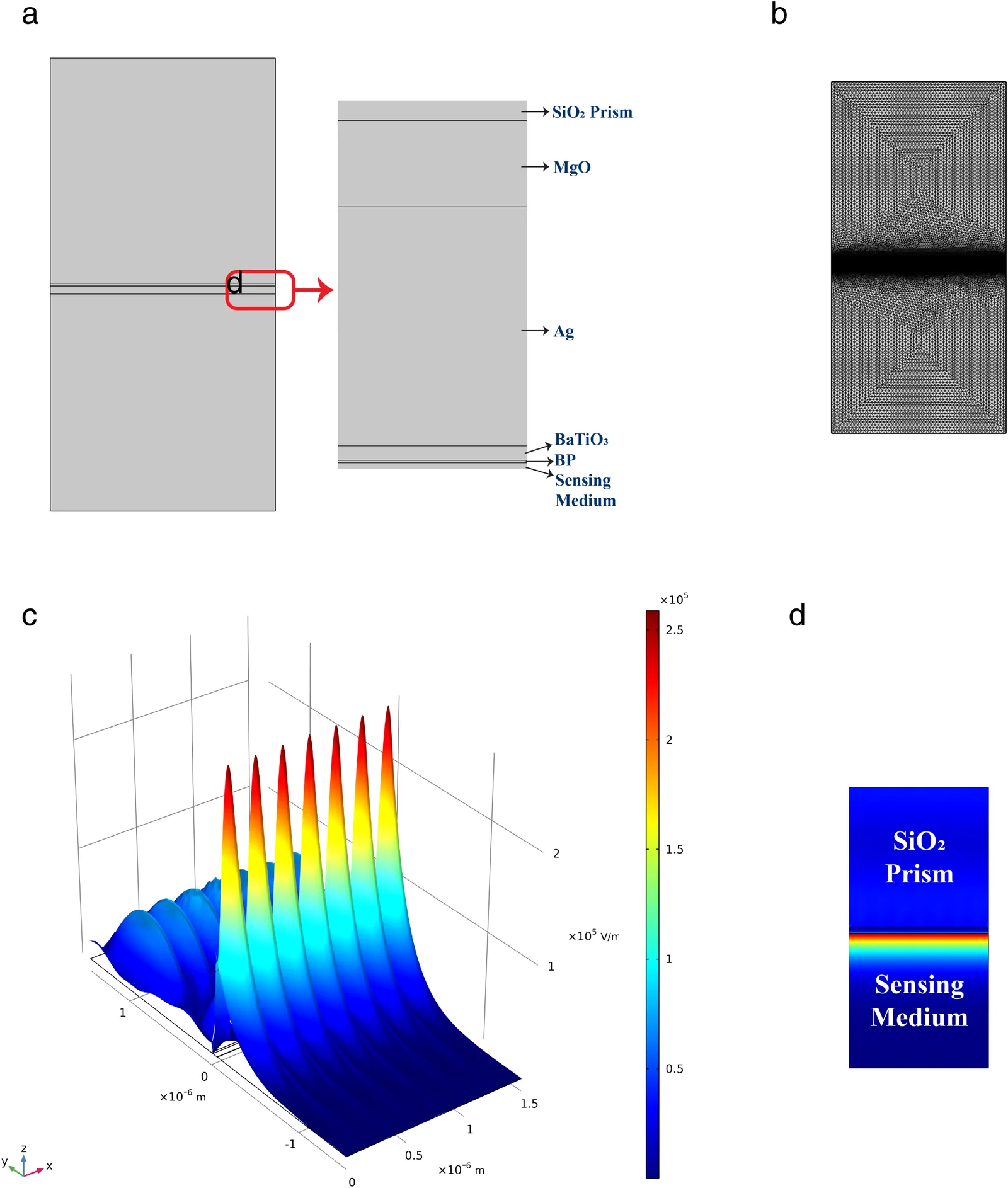
(a) Schematic of the proposed biosensor using COMSOL, (b) Mesh view of the design under multilayer sensor structure, (c) 2D electric field distribution (V/m) at a resonant angle of 80.11 deg, and (d) 3D image of the z component of electric field (V/m) at resonance angle 80.11 deg as imaged through the height surface.

In order to further validate the analytical results obtained using the transfer matrix method (TMM), the proposed SPR sensor structure has also been analyzed using the finite element method (FEM), and the results are presented in Fig 2(a), which depicts the electric field intensity distribution in the proposed SPR sensor structure consisting of the SiO_2_ prism, MgO layer, Ag layer, BaTiO_3_ layer, BP, and the sensing medium (SM). The mesh distribution in the SPR structure, as presented in Fig 2(b), demonstrates the physics-controlled fine mesh with higher density in the SPR region due to the variation in the electric field. The electric field intensity profile at the resonance angle, as illustrated in Fig 2(c), indicates the electric field enhancement at the Ag/BaTiO_3_/BP interface, confirming the strong coupling between the incident electromagnetic wave and SPPs, followed by the gradual decrease of the electric field in the direction of plasmon propagation in the metal layer and the dielectric medium. In addition, the three-dimensional diagram of the electric field intensity, as presented in Fig 2(d), clearly demonstrates the electric field localization at the sensing interface, with the peaks and valleys confirming the stable propagation of plasmons in the metal-dielectric interface. Moreover, the optimized thickness of the MgO, Ag, BaTiO_3_, and BP layers contributes to the confinement of the electromagnetic field and the light-matter interaction in the sensing region, thus improving the sensing characteristics of the proposed SPR biosensor for the detection of fat in milk.

### 2.5 SPR performance metrics

One of the main parameters for performance measurement of SPR biosensors is the sensitivity (S). The sensitivity is defined by the ratio of the variation of refractive index (*Δn*) and variation of resonance angle (*Δθ*_*res*_) with respect to each other. It is also defined by [60]:


$$\begin{array}{c} \text{S}=\frac{\Delta \theta_{\text{res}}}{\Delta \text{n}}(\text{in deg}/\text{RIU }) \end{array}$$ (12)

There are several other key parameters which have to be considered to estimate the overall efficiency of SPR sensors. Quality Factor (QF), Detection Accuracy (DA), Minimum reflectance (R_min_), Full Width at Half Maximum (FWHM), and Figure of Merit (FOM) are commonly being used to test the efficiency of SPR sensors. Below is a description of these parameters [61]:


$$\begin{array}{c} \text{FWHM}=\theta_{\text{max}}-\theta_{\text{min}}(\text{deg}) \end{array}$$ (13)

$$\begin{array}{c} \text{QF}=\frac{\text{S}}{\text{FWHM}}\left(\text{in RI}{\text{U}}^{-\text{1}}\right) \end{array}$$ (14)

$$\begin{array}{c} \text{DA }=\frac{\text{1}}{\text{FWHM}}\left({\text{deg}}^{-\text{1}}\right) \end{array}$$ (15)

$$\begin{array}{c} \text{FOM}=\text{S}\times \frac{\text{1}-{\text{R}}_{\text{min}}}{\text{FWHM}} \end{array}$$ (16)

## 3. Iterative search-based optimization of the SPR sensor parameters

### 3.1 Development of the proposed SPR biosensor

The SPR biosensor, as proposed, is designed by optimizing the geometric parameters of each of the layers of the multilayer configuration, which shows a strong reflectance variation as a function of the angle of incidence of the light beam. The sensing region of the SPR biosensor is implemented by optimizing the multilayer configuration of the MgO/Ag/BaTiO_3_/BP layers deposited over the SiO_2_ prism, which enables the detection of fat content in the milk with high sensitivity.

**Fig 3:**
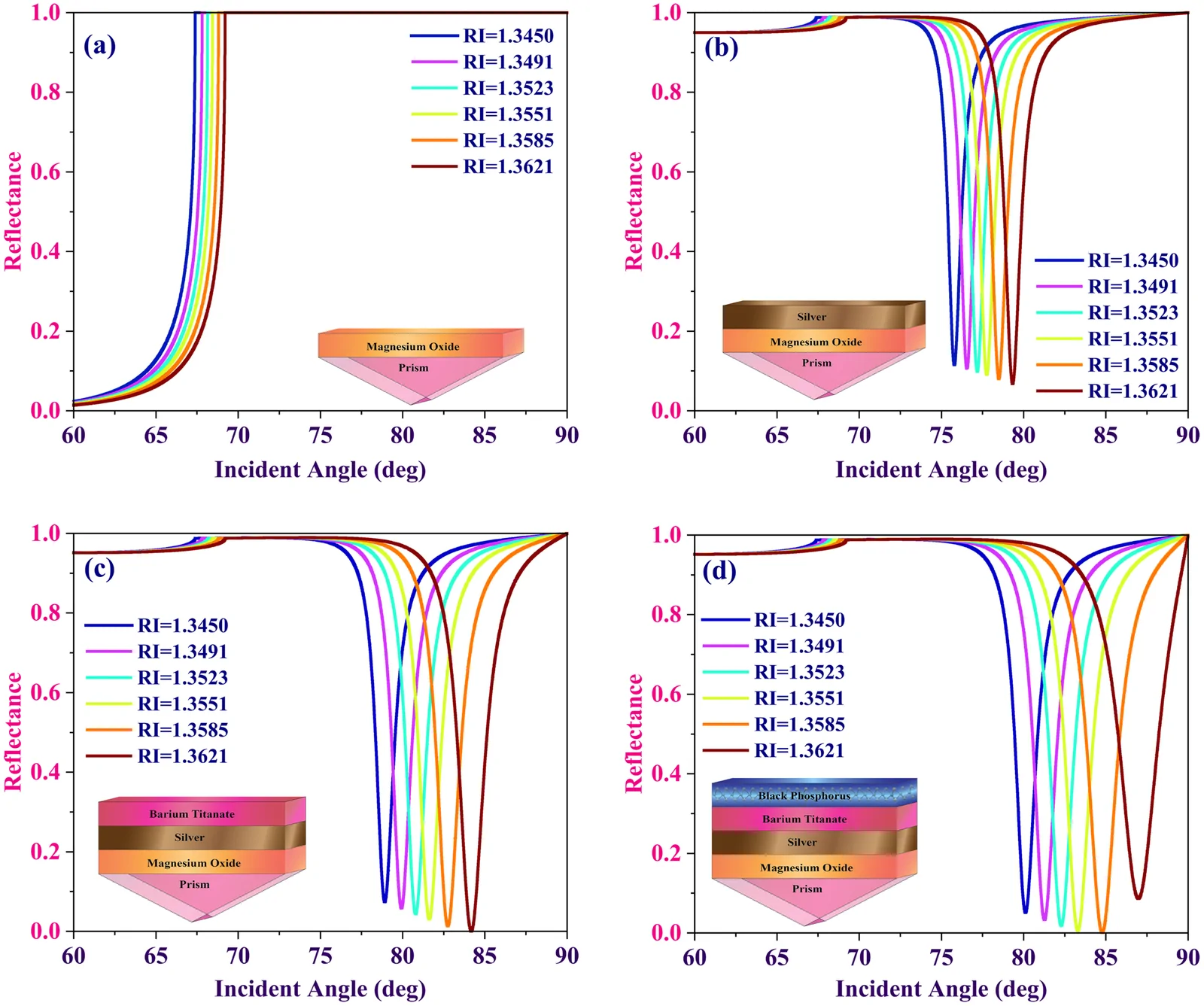
Reflectance vs Incident Angle (deg) curve for (a) SiO_2_ + MgO, (b) SiO_2_ + MgO + Ag, (c) SiO_2_ + MgO + Ag + BaTiO_3,_ and (d) SiO_2_ + MgO + Ag + BaTiO_3_ + BP.

The BP layer helps to improve the light-matter interaction and the confinement of the electromagnetic fields within the sensing region of the SPR biosensor, which improves the overall performance of the SPR biosensor. The reflectance curves for different RI values of the sensing region, which varies according to the fat content of the milk, are shown in Fig 3. The shift in the resonance angle of the SPR biosensor for increasing RI is shown, which confirms the high sensitivity of the SPR biosensor as proposed. The multilayer configuration of the SPR biosensor, including the BP layer, is shown to improve the performance of the SPR biosensor by enhancing the light-matter interaction within the SPR structure. The systematic structural refinement significantly enhances plasmonic response and overall sensor efficiency in Table 2.

**Table 2:** Performance metrics of the biosensor at different development stages for an analyte RI of 1.3621.

Configuration	Sensitivity (deg/RIU)	R_min_	QF (RIU^-1^)	DA (deg^-1^)
SiO_2_ + MgO	0	0.013	0	0
SiO_2_ + MgO + Ag	207.13	0.067	177.79	0.858
SiO_2_ + MgO + Ag + BaTiO_3_	307.54	0.000	165.52	0.538
SiO_2_ + MgO + Ag + BaTiO_3_ + BP	401.40	0.086	148.06	0.368

### 3.2 Impact of choosing prism

**Fig 4:**
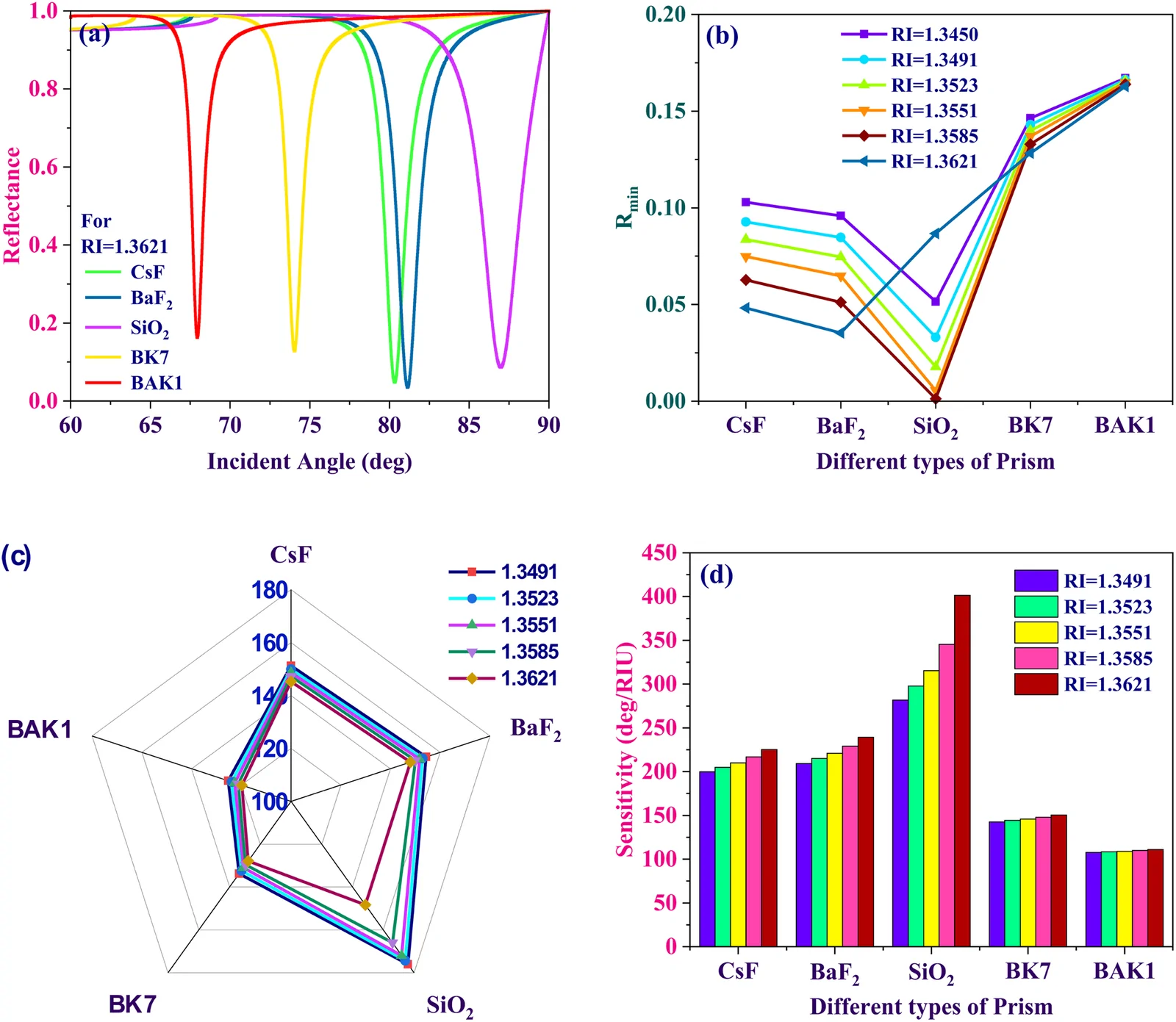
Performance analysis using different prism: (a) Reflectance vs Incident Angle (deg) for RI = 1.3621, (b) R_min_, (c) QF, and (d) Sensitivity.

In order to select the most appropriate material for the coupling prism of the SPR biosensor for fat detection in milk, a comparative analysis of the sensing capabilities of different materials, including SiO_2_, BaF_2_, CsF, BK7, and BAK1, was performed. As shown in Fig 4, among all the materials, the SiO_2_-based SPR configuration has the best sensing performance with the highest RI of 1.3621.

The SiO_2_-based SPR configuration can achieve the highest sensitivity of 401.40 deg/RIU, the lowest R_min_ of 0.086, the highest QF of 148.33 RIU^-1^, and the highest DA of 0.369 deg^-1^, while the BaF_2_-based SPR configuration can only achieve a relatively low sensitivity of 239.29 deg/RIU, the lowest R_min_ of 0.035, the highest QF of 147.989 RIU^-1^, and the highest DA of 0.618 deg^-1^, which is relatively low. Similarly, the CsF prism offers a sensitivity of 225.14 deg/RIU, which is associated with a R_min_ of 0.048, a QF of 145.25 RIU^-1^, and a DA of 0.645 deg^-1^. The BK7 prism offers a sensitivity of 150.35 deg/RIU, which is associated with a R_min_ of 0.128, a QF of 127.84 RIU^-1^, and a DA of 0.850 deg^-1^, while the BAK1 prism offers the lowest sensitivity of 110.87 deg/RIU, which is associated with a R_min_ of 0.162, a QF of 119.73 RIU^-1^, and a DA of 1.0799 deg^-1^. The superior performance of the SiO_2_ prism can be attributed to the optimal refractive index, which enables the enhancement of the momentum matching between the incident optical wave and the surface plasmon waves at the interface of the metal and the dielectric material, thereby enhancing the excitation of the surface plasmons and the electromagnetic fields. Thus, the SiO_2_ prism is identified as the most appropriate coupling medium for the proposed SPR biosensor, which enables the detection of fat content in milk with high sensitivity and accuracy.

### 3.3 MgO layer variation

**Fig 5:**
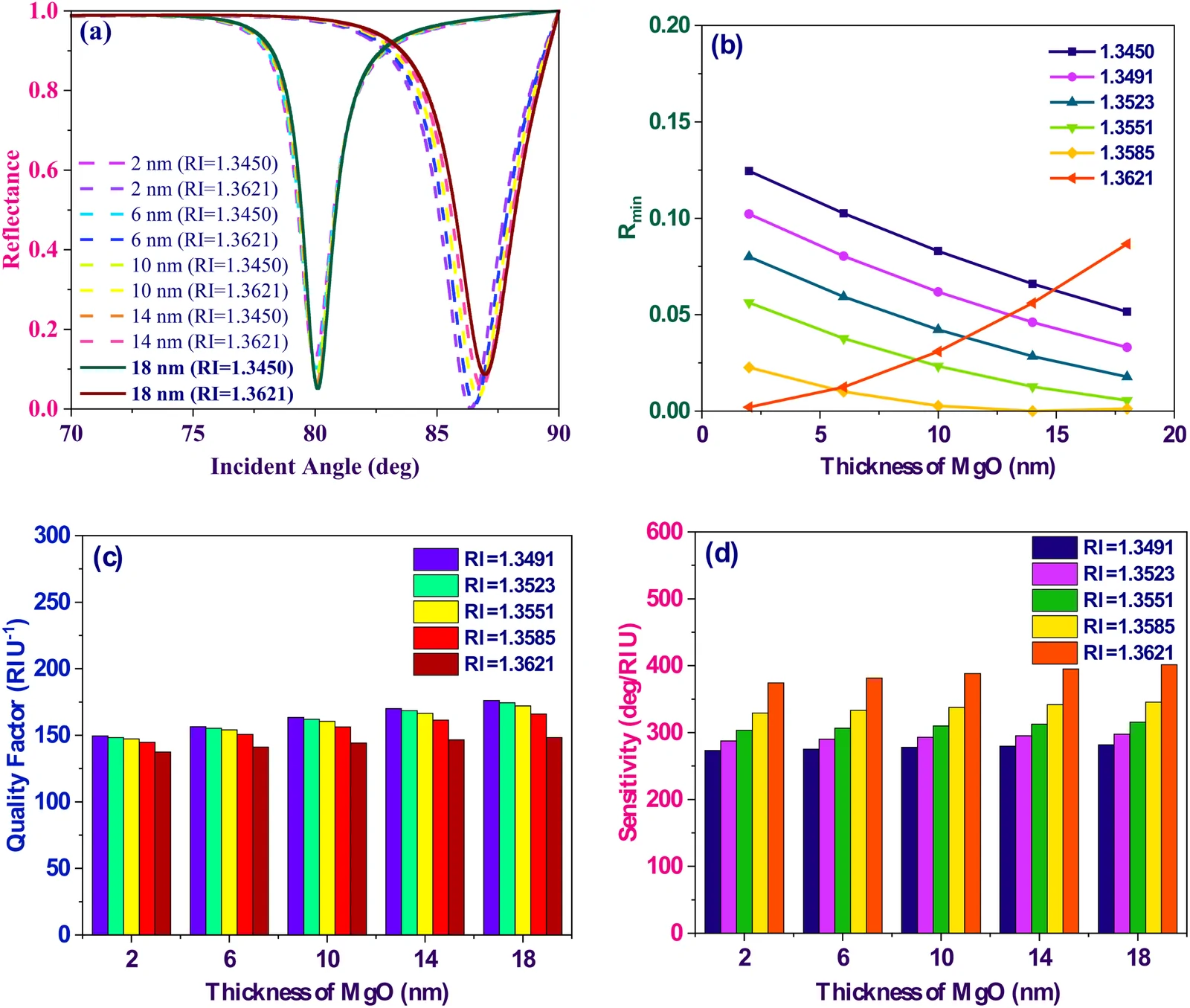
Variation in the thickness of MgO layer: (a) Reflectance vs Incident Angle (deg), (b) R_min_, (c) QF, and (d) Sensitivity.

A systematic optimization of the MgO thickness was performed to obtain the best configuration for maximizing the sensing capabilities of the proposed SPR biosensor for fat detection in milk, as shown in Fig 5. The purpose of using MgO in the developed multilayer SPR sensor is mainly for improving the adhesion (buffering) function of the system that helps in achieving greater stability at the prism-Ag interface. Besides this, MgO also serves some other purposes in terms of optical behavior of the developed SPR sensor. Owing to its dielectric nature, MgO changes the condition of surface plasmon coupling and electromagnetic field distribution at the metal-dielectric interface, causing shifting in resonance angle and better sensing properties. The thickness of the MgO film was varied from 2 to 18 nm, and the corresponding performance parameters were critically examined at the highest RI of 1.3621.

It was observed that at a low thickness of 2 nm, the SPR biosensor offers a sensitivity of 374.26 deg/RIU with a relatively low R_min_ of 0.002 and a DA of 0.367 deg^-1^, which is inadequate for the proper confining of the electromagnetic fields. However, as the thickness increases to 6 and 10 nm, the SPR biosensor offers a sensitivity of 381.28 deg/RIU with QF of 141.11 RIU^-1^ and 388.24 deg/RIU with QF of 144.16 RIU^-1^, respectively, with moderate R_min_ of 0.012 and 0.030, but fails to provide optimal plasmon coupling. Further increasing the value to 14 nm yields sensitivity of 394.85 deg/RIU with R_min_ of 0.056, along with DA of 0.371 deg^-1^. However, the sensitivity and accuracy are not optimal. Interestingly, for the MgO thickness of 18 nm, the sensor exhibits the maximum sensitivity of 401.40 deg/RIU, with the R_min_ remaining constant at 0.086, which is below the critical reflectance threshold of 0.1, along with the QF being 148.33 RIU^−1^ and the DA being 0.369 deg^-1^. Thus, the optimal momentum matching, the confinement of the electromagnetic field, and the plasmonic effect are maximized. The lower values of the MgO thickness do not yield optimal sensitivity along with the reflectance. Thus, the optimal MgO thickness is considered to be 18 nm, which maximizes the sensitivity and the reflectance.

### 3.4 Ag layer variation

**Fig 6:**
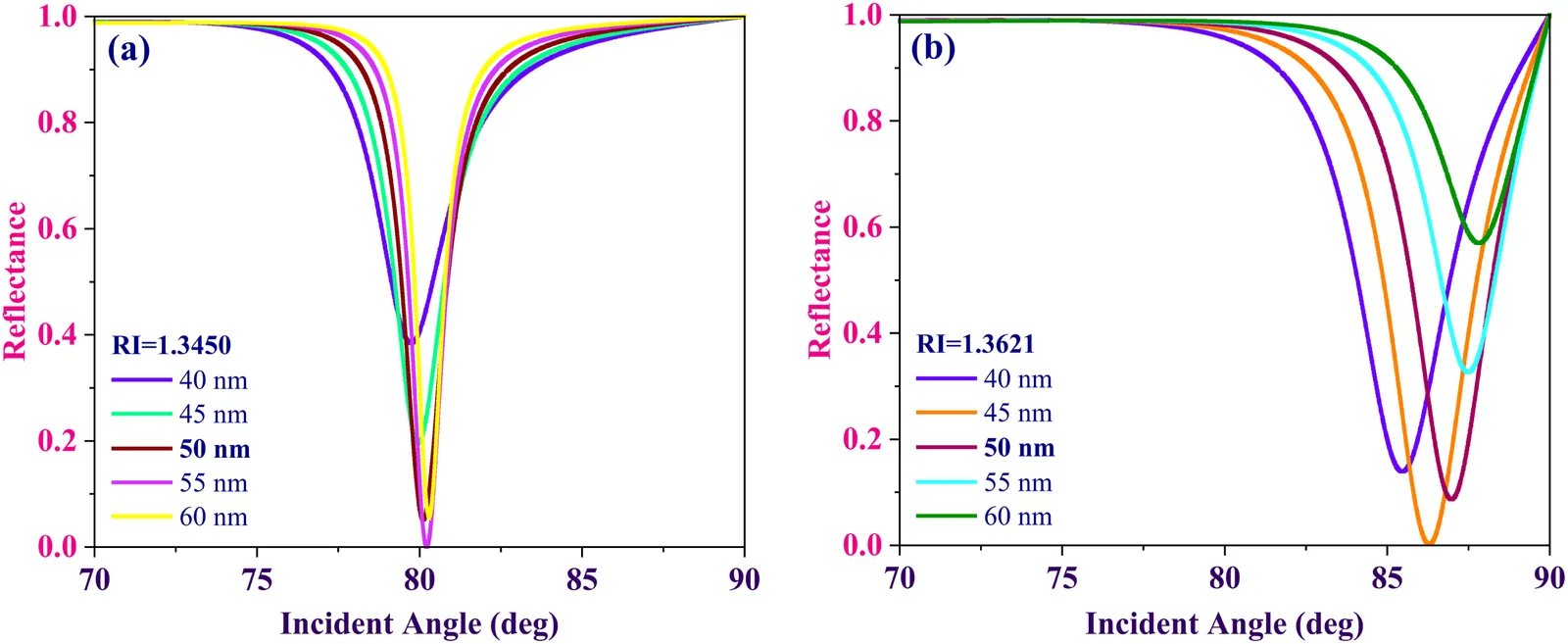
Variation in the thickness of Ag layer: (a) Reflectance vs Incident Angle (deg) for RI = 1.3450, (b) Reflectance vs Incident Angle (deg) for RI = 1.3621.

Moreover, a detailed parametric analysis for the Ag layer thickness is performed to find the optimum thickness for achieving the best possible sensor performances for the proposed SPR sensor for fat detection in milk, as shown in Fig 6. The thickness is varied from 40 to 60 nm, as shown in Table 3 and corresponding performances are evaluated at the RI 1.3621. At 40 nm thickness, the sensor’s sensitivity is low at 334.67 deg/RIU, and the R_min_ is high at 0.139. Moreover, the sensor’s DA is low at 0.302 deg^-1^ with QF of 101.11 RIU^-1^. By increasing the thickness to 45 nm, the sensor’s sensitivity is improved to 370.52 deg/RIU, and the R_min_ is significantly reduced to 0.002 with QF 128.78 RIU^-1^. However, the sensor’s performances are not satisfactory at this thickness. By increasing the thickness to 50 nm, the sensor’s performances are found to be optimum, and the sensor’s sensitivity is maximized to 401.40 deg/RIU with the R_min_ is low at 0.0867, and it is below the critical threshold of 0.1. Moreover, the sensor’s QF is found to be optimum at 148.33 RIU^-1^, and the DA is 0.369 deg^-1^. This indicates optimal momentum matching and efficient excitation of surface plasmons. Further increasing the thickness to 55 nm and 60 nm results in higher sensitivities of 424.97 deg/RIU and 440.11 deg/RIU, respectively. However, these configurations suffer from increased R_min_ values of 0.326 and 0.569, along with degraded DA, indicating poor resonance sharpness and unstable plasmon excitation. Therefore, despite the higher sensitivity at larger thicknesses, the inability to maintain R_min_ below 0.1 makes these configurations unsuitable. Consequently, the Ag thickness of 50 nm is selected as the optimal value, as it provides the best trade-off between high sensitivity and controlled reflectance, ensuring stable, sharp, and efficient SPR response for accurate fat detection in milk.

**Table 3:** Performance analysis for varying different Ag thickness.

Thickness of Ag layer (nm)	Analyte RI	Sensitivity (deg/RIU)	R_min_	DA (deg^-1^)	QF (RIU^-1^)
	1.3491	257.07	0.355	0.363	93.41
	1.3523	269.17	0.325	0.351	94.71
40	1.3551	282.07	0.291	0.340	96.14
	1.3585	302.44	0.234	0.324	98.26
	1.3621	334.67	0.139	0.302	101.11
	1.3491	271.46	0.165	0.485	131.65
	1.3523	285.61	0.136	0.462	132.04
45	1.3551	301.08	0.104	0.439	132.34
	1.3585	326.51	0.055	0.404	132.08
	1.3621	370.52	0.002	0.347	128.78
	**1.3491**	**281.70**	**0.033**	**0.625**	**176.06**
	**1.3523**	**297.67**	**0.017**	**0.585**	**174.38**
50	**1.3551**	**315.44**	**0.005**	**0.545**	**171.99**
	**1.3585**	**345.48**	**0.0013**	**0.480**	**166.01**
	**1.3621**	**401.40**	**0.086**	**0.369**	**148.33**
	1.3491	289.02	0.003	0.770	222.66
	1.3523	306.30	0.012	0.710	217.54
55	1.3551	325.74	0.030	0.646	210.56
	1.3585	359.55	0.085	0.543	195.51
	1.3621	424.97	0.326	0.372	158.21
	1.3491	294.14	0.082	0.907	266.92
	1.3523	312.19	0.114	0.822	256.73
60	1.3551	332.97	0.158	0.733	244.29
	1.3585	369.55	0.255	0.591	218.41
	1.3621	440.11	0.569	0.368	162.16

### 3.5 BaTiO_3_ layer variation

**Fig 7:**
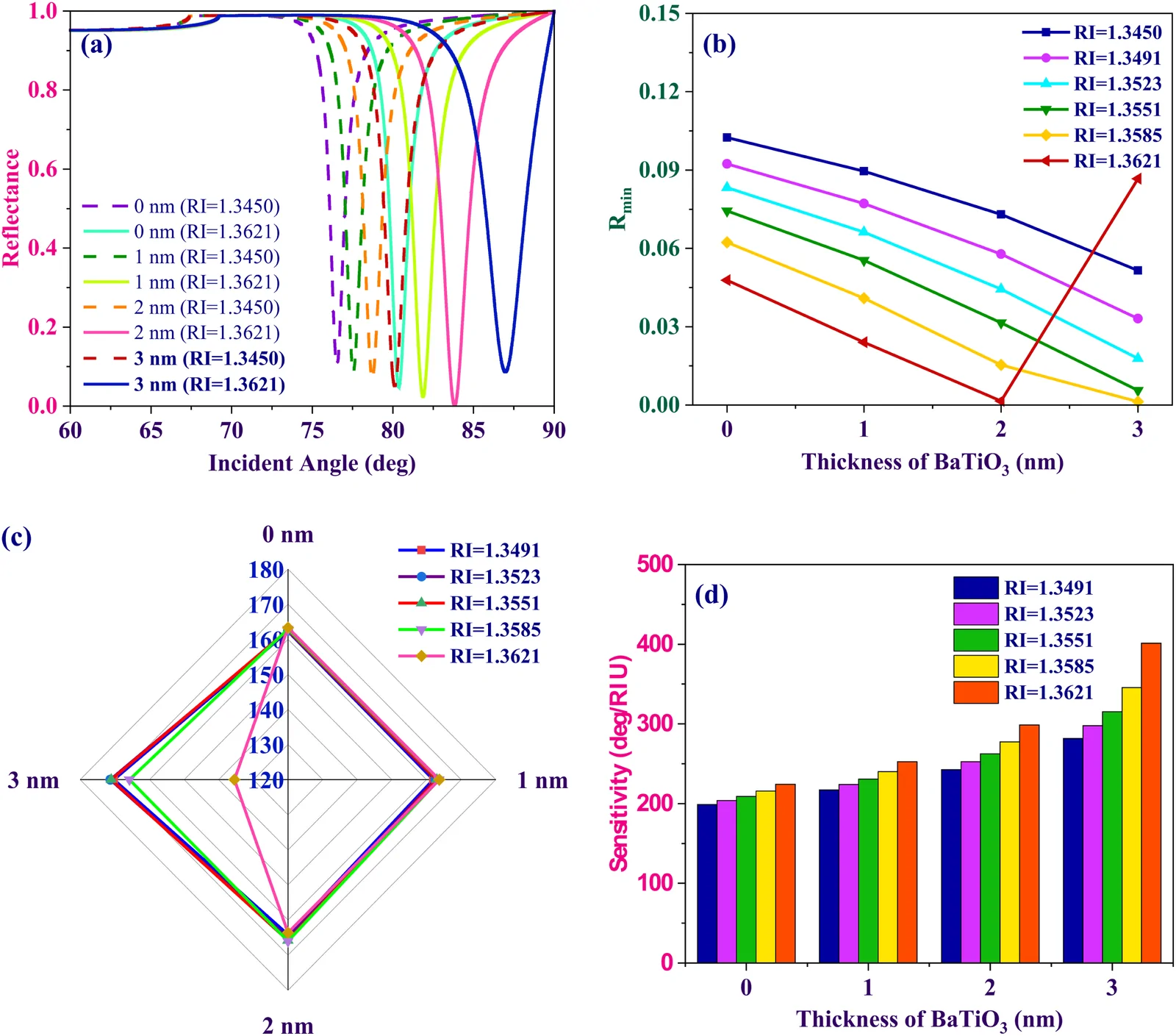
Variation in the thickness of BaTiO_3_ layer: (a) Reflectance vs Incident Angle (deg), (b) R_min_, (c) FOM, and (d) Sensitivity.

A detailed study of the thickness of the BaTiO_3_ layer was performed to find its optimum value for improving the efficiency of the proposed SPR biosensor for fat detection in milk, as shown in Fig 7. In this study, the thickness of the BaTiO_3_ layer was varied over the range of 0 nm to 3 nm. Then, the sensing parameters were calculated for this maximum RI value of 1.3621. The sensor shows a very low sensitivity of 224.15 deg/RIU with a R_min_ of 0.047 and high accuracy of 0.765 deg^-1^ at a thickness of 0 nm. This shows that there is no dielectric layer. Hence, the SPR signal is very low. By increasing the thickness of the BaTiO_3_ layer to 1 nm, the sensitivity of the sensor increases to 252.45 deg/RIU with a R_min_ of 0.024. However, further increasing the thickness to 2 nm shows that the sensitivity is 298.53 deg/RIU with a significantly low R_min_ of 0.001. However, the DA is relatively higher. It is interesting to note that for a thickness of 3 nm, the highest sensitivity is observed to be 401.40 deg/RIU along with the R_min_ of 0.0867, which is below the critical value of 0.1 and is associated with high QF value of 148.33 RIU^-1^ and DA of 0.369 deg^-1^. This configuration is associated with optimal dielectric loading, momentum matching, and high electromagnetic field confinement on the metal/dielectric interface. On the other hand, for other thickness values, high sensitivity along with controlled resonance characteristics are not observed. Hence, for BaTiO_3_ thickness of 3 nm, optimal thickness is observed for efficient fat detection in milk.

### 3.6 Impact of TMDC layer

**Fig 8:**
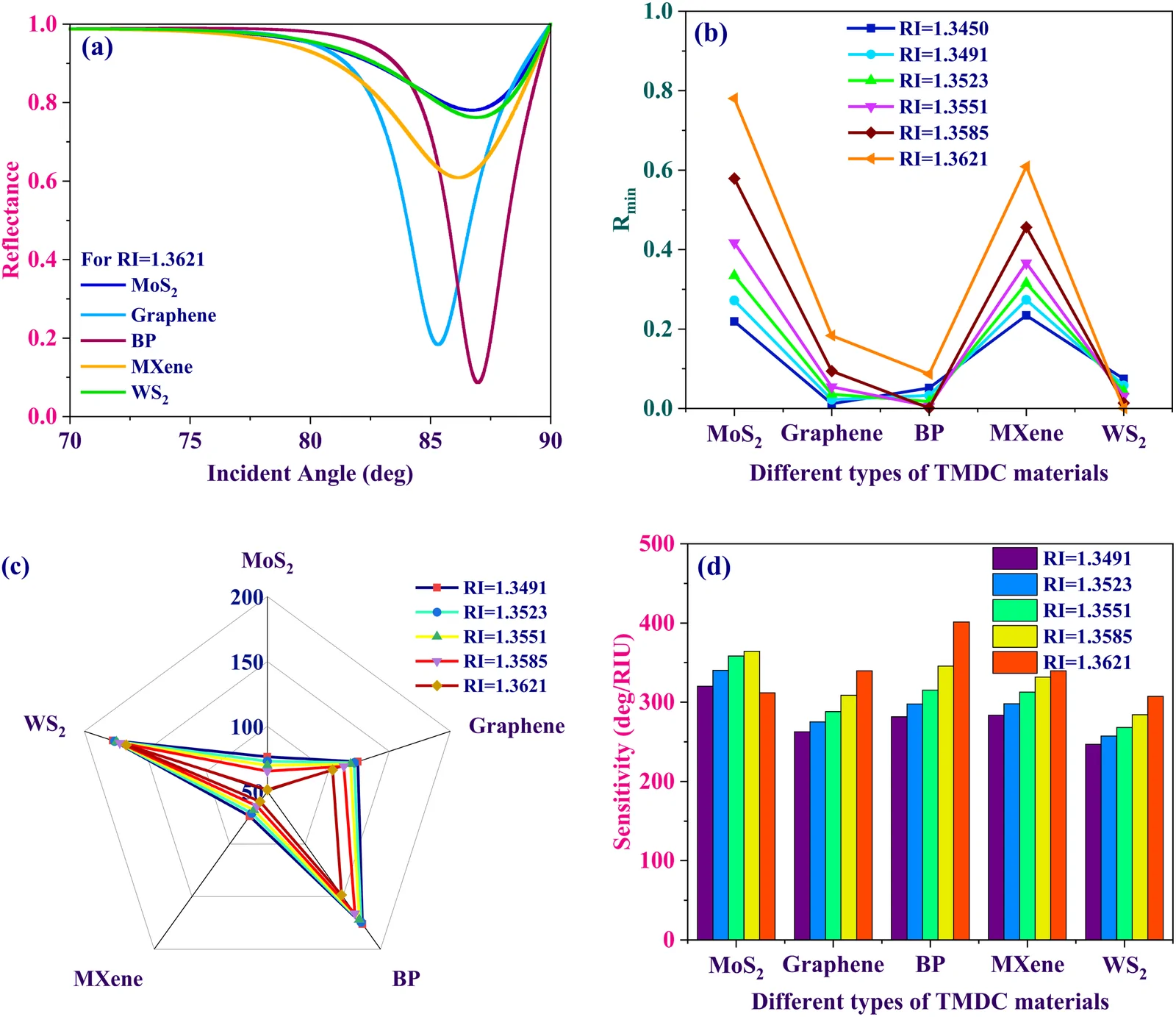
Performance evaluation using various TMDC layer: (a) Reflectance vs Incident Angle (deg) for RI = 1.3621, (b) R_min_, (c) QF, and (d) Sensitivity.

A detailed comparative analysis of different types of 2D materials such as black phosphorus (BP), graphene, MXene, MoS_2_, and MoSe_2_ was performed to select the best option for improving the efficiency of the proposed SPR biosensor for fat detection in milk, as shown in Fig 8. The analysis was performed for all materials at the highest value of RI 1.3621 and on the basis of different parameters such as sensitivity, R_min_, QF, and DA. Among all materials, the BP-based configuration provides better performance compared to other materials in terms of sensitivity up to 401.40 deg/RIU and R_min_ up to 0.086, which is below the critical value of 0.1. At the same time, the proposed configuration provides better values of QF up to 148.33 RIU^-1^ and accuracy up to 0.369 deg^-1^ compared to other materials. On the other hand, graphene provides less sensitivity up to 339.70 deg/RIU and high R_min_ up to 0.1835 with QF of 103.56 RIU^-1^. Similarly, the sensitivity of MXene is 339.64 deg/RIU and QF of 59.67 RIU^-1^, with a high R_min_ of 0.609, indicating low resonance characteristics. The configuration of MoS_2_ offers a sensitivity of 311.87 deg/RIU, with a R_min_ of 0.780 and QF of 51.33 RIU^-1^, while the configuration of MoSe_2_ offers a sensitivity of 330.46 deg/RIU, with a R_min_ of 0.762 and QF of 57.47 RIU^-1^, indicating low performance due to high reflectance and low field confinement. It can be seen that some materials exhibit moderate sensitivity, but the reflectance condition is not maintained at a low level, indicating unstable plasmon excitation and broad resonance profiles. The high performance of BP can be attributed to its unique optical properties, which are anisotropic, and its high mobility, which enables efficient light-matter interactions and high electromagnetic field confinement at the sensing interface. Therefore, BP is chosen as the optimum 2D material, as it offers the best balance between the highest sensitivity and reflectance, ensuring highly efficient, stable, and reliable SPR sensing for fat detection in milk.

### 3.7 Optimization of SPR sensor layer thickness using brute-force method

Milk fat content is one of the significant parameters for determining dairy product quality, nutritional value, and possible adulteration, thus requiring the development of sensitive and accurate detection techniques. Among all possible sensing techniques, SPR sensors are identified as a potential option for detecting dairy product quality. Therefore, in this study, a multilayer SPR sensor is designed to optimize the performance of the sensor for detecting milk fat content. In this study, a multilayer SPR sensor is designed by incorporating a SiO_2_/MgO/Ag/BaTiO_3_/BP multilayer structure. SiO_2_ is used as a prism for exciting SPR, whereas Ag is used for enhancing the performance of the SPR sensor by exploiting its superior optical properties. Dielectric layers, including MgO and BaTiO_3_, are incorporated to further optimize the performance of the SPR sensor by enhancing electromagnetic field confinement. An ultrathin layer of BP is used for enhancing analyte adsorption due to its superior optical and electronic properties.

To attain optimal sensing performance, a brute-force optimization technique is implemented to obtain the optimal layer thickness. The technique involves an exhaustive search for all possible parameter values within specified limits. For angular interrogation SPR sensors, an intensive resonance dip is critical for determining the resonance angle correctly. The smaller R_min_ implies better coupling of the light from the beam into surface plasmons and, thus, the higher plasmon excitation and better resonance contrast. Therefore, one could consider R_min_ < 0.1 as a practical criterion for achieving high enough resonance dip while optimizing the sensor design. The method ensures an optimal solution by avoiding local minima. In order to determine the relationship between structural parameters and performance of sensors, the Multiple Linear Regression (MLR) model was utilized. Simulation dataset was created based on Transfer Matrix Method (TMM) by changing thicknesses of MgO (0–20 nm), Ag (30–60 nm), and BaTiO_3_ (1–5 nm) layers, while fixing the thickness of silicon layer to be 0.53 nm. Thus, 726 samples were obtained, where the input features were thicknesses of MgO, Ag, and BaTiO_3_ layers, and output features were sensitivity, Rmin, FWHM, Q, DA, and FOM. Training data constituted 80% of all available dataset, while testing data were the remaining 20%. The performance of regression model for prediction was determined via Root Mean Square Error (RMSE), Mean Absolute Error (MAE) and coefficient of determination (R^2^). Trained regression model was further incorporated into Brute Force optimization algorithm to find the optimal set of layer thicknesses meeting the requirements of design process. The Multiple Linear Regression (MLR) analysis was employed on the data set created by TMM in order to formulate the mathematical relation between the structural parameters and sensor performance. The input parameters were made up of the thickness of MgO, Ag, and BaTiO_3_ layers, while the response variables were sensitivity, R_min_, FWHM, QF, DA, and FOM. In comparison to traditional optimization based on sweeping through values of the parameters resulting in maximum sensitivity of 401.40 deg/RIU, the proposed hybrid ML-assisted optimization resulted in increased sensitivity of 424.61 deg/RIU and improved sensing performance.

**Fig 9:**
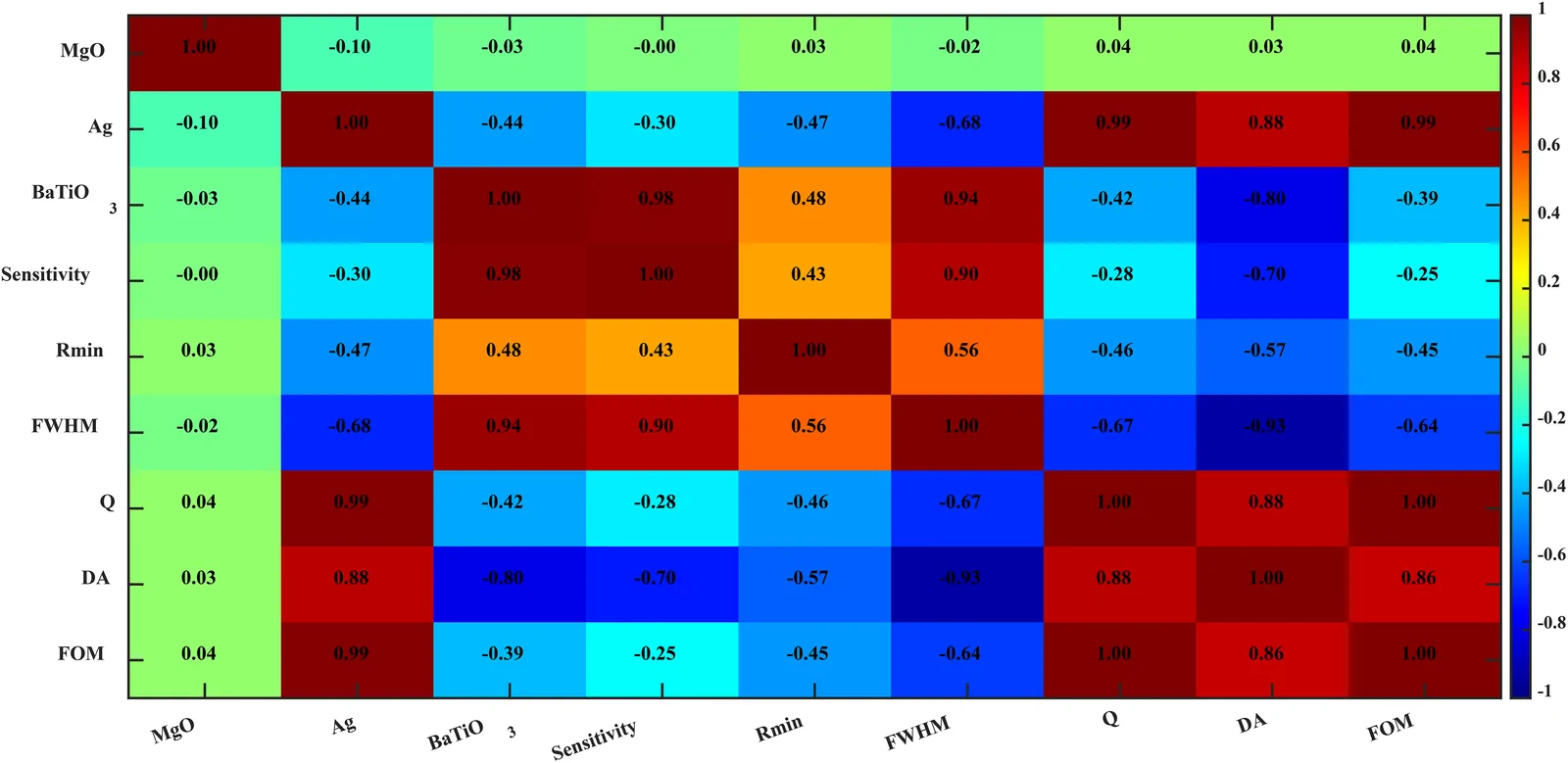
Pearson correlation heat map of design parameters and performance parameters of the proposed SPR sensor.


Before the regression analysis was carried out, Pearson correlation matrices were determined to examine the monotonicity between the input parameters and their corresponding output parameters, as shown in Fig 9. Pearson correlation matrices provide a quantitative measure of the linear relationship between the input parameters and their corresponding output parameters. In this regard, the Pearson correlation coefficient *r* is used to assess the extent of correlation that exists between two parameters, *x* and *y*, where *m* observations are made. The mathematical representation of the Pearson correlation coefficient calculation is shown in the following equation [62]:

$$\begin{array}{c} =\frac{m\left(\sum xy\right)-\left(\sum x\right)\left(\sum y\right)}{\sqrt{\left[m\sum x^{\text{2}}-{\left(\sum x\right)}^{\text{2}}\right]\times \left[m\sum y^{\text{2}}-{\left(\sum y\right)}^{\text{2}}\right]}} \end{array}$$ (17)

The aim of linear regression analysis is to determine the line that most closely fits the relationship between the variables. It is also known as the regression line or line of greatest fit. It can be expressed by the equation below:


$$\begin{array}{c} Y=\beta_{\text{0}}+\beta_{\text{1}}X_{\text{1}}+\beta_{\text{2}}X_{\text{2}}+\ldots \beta_{n}X_{n} \end{array}$$ (18)

Where Here, Y is the dependent (response or target) variable, whereas X_1_, X_2_, …, X_n_ represent the independent (predictor or feature) variables. The symbol β₀ stands for the intercept, which is the value of the dependent variable when all the independent variables are equal to zero. Similarly, β_1_, β_2_, …, β_n_ represent the regression weights that measure the importance of each independent variable on the dependent variable. The obtained equations were capable of measuring the effect of each structural parameter on the sensor performance. The models that were developed showed very low mean square error (0.0000 ~ 3.4763), indicating their good predictive ability.

### 3.8 Optimization strategy and comparative analysis of iterative and brute force techniques

The performance metrics of the proposed multilayer SPR biosensor, using both iterative and brute force optimization techniques, show a high level of agreement between the two optimization techniques, as can be seen in Table 4. In the iterative optimization technique, the optimal configuration can be obtained by setting the thickness of the MgO layer to 18 nm, the thickness of the Ag layer to 50 nm, and the thickness of the BaTiO_3_ layer to 3 nm, keeping the thickness of the BP layer fixed at 0.53 nm. This configuration can significantly enhance the overall performance of the proposed biosensor. In the brute force optimization technique, the thickness of the BP layer is fixed at 0.53 nm, whereas the thickness of the MgO layer varies from 0 to 20 nm in steps of 1 nm, the thickness of the Ag layer varies from 30 to 60 nm in steps of 1 nm, and the thickness of the BaTiO_3_ layer varies from 1 to 5 nm with increments of 0.1 nm. Thus, through this exhaustive search, it is found that the optimum configuration is MgO = 17 nm, Ag = 48 nm, and BaTiO_3_ = 3.2 nm. It is important to mention that the 0.1 nm step in thickness that was used for optimization is just the step size used for numerical scanning of the parameter space and does not reflect the needed precision of fabrication at all. The small step size has been chosen in order to find the exact point of the global minimum as well as to detect the nuances in behavior of the SPR signal. Nevertheless, the designed sensor utilizes practically attainable nominal layer thicknesses. While the numerical optimization has shown that the almost optimal thickness of the BaTiO_3_ layer is about 3.2 nm, we have chosen 3 nm taking into account the possibility to produce this thickness by known methods of thin film fabrication, e.g., Atomic Layer Deposition (ALD), Magnetron Sputtering, and Pulsed Laser Deposition (PLD) [63,64]. Thus, it is found that the iterative method is more favorable due to its simplicity and better compatibility with practical constraints. In addition, the similarity in results obtained from both methods proves that this iterative method is computationally efficient and reliable without compromising the performance of the biosensor.

**Table 4:** Evaluation metrics of the proposed multilayer SPR biosensor.

Sensing Medium’s RI	Resonance Angle	R_min_	FWHM	Sensitivity	DA	FOM	QF
	(deg)		(deg)	(deg/RIU)	(deg^-1^)		(RIU^-1^)
**Using Iterative Method**							
1.3450	80.11	0.051	1.497	–	–	–	–
1.3491	81.26	0.033	1.600	281.70	0.625	170.23	176.06
1.3523	82.28	0.017	1.707	297.67	0.585	171.27	174.38
1.3551	83.29	0.005	1.834	315.44	0.545	171.04	171.99
1.3585	84.77	0.001	2.083	345.48	0.480	165.63	165.85
1.3621	86.97	0.086	2.711	401.40	0.368	135.23	148.06
**Using Brute Force Algorithm**							
1.3450	80.35	0.098	1.716	–	–	–	–
1.3491	81.53	0.072	1.828	287.56	0.547	145.97	157.30
1.3523	82.57	0.047	1.945	304.65	0.514	149.15	156.63
1.3551	83.62	0.024	2.085	324.05	0.479	151.63	155.42
1.3585	85.18	0.001	2.367	357.92	0.422	151.07	151.21
1.3621	87.61	0.099	3.054	424.61	0.327	125.16	139.03

### 3.9 Analysis of electric field distribution and penetration depth

**Fig 10:**
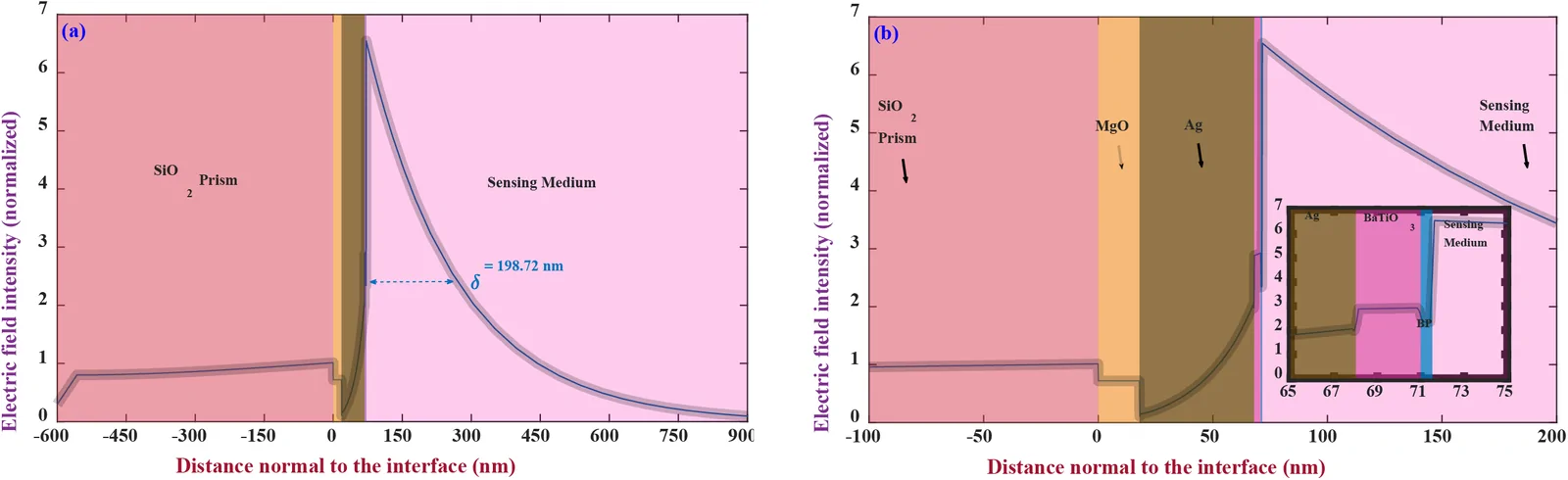
(a) Penetration depth using FDTD method, and (b) FDTD based analysis of electric field intensity vs normalized distance for the proposed sensor.

The efficient excitation of surface plasmons in the proposed sensor configuration is validated by the electric field intensity distribution calculated at an analyte of RI = 1.3450 and resonance angle of 80.11 deg, as shown in Fig 10(b). The propagation of an electromagnetic wave in the sensing medium leads to an exponential decay in electric field intensity from its maximum value at the sensor-analyte interface. The penetration depth is found to be 198.72 nm, as shown in Fig 10(a), indicating an increased sensing region in the analyte medium. The penetration depth is generally described as the distance in meters from the interface at which the electric field intensity decays to 1/e (or about 37%) of its initial value [65,66]. The improved sensing capabilities of the sensor can be attributed to the electric field intensity concentration at the interface as well as the increased penetration depth of the surface plasmon wave in the analyte medium.

### 3.10 Performance evaluation of linearity and dynamic range in the SPR biosensor

**Fig 11:**
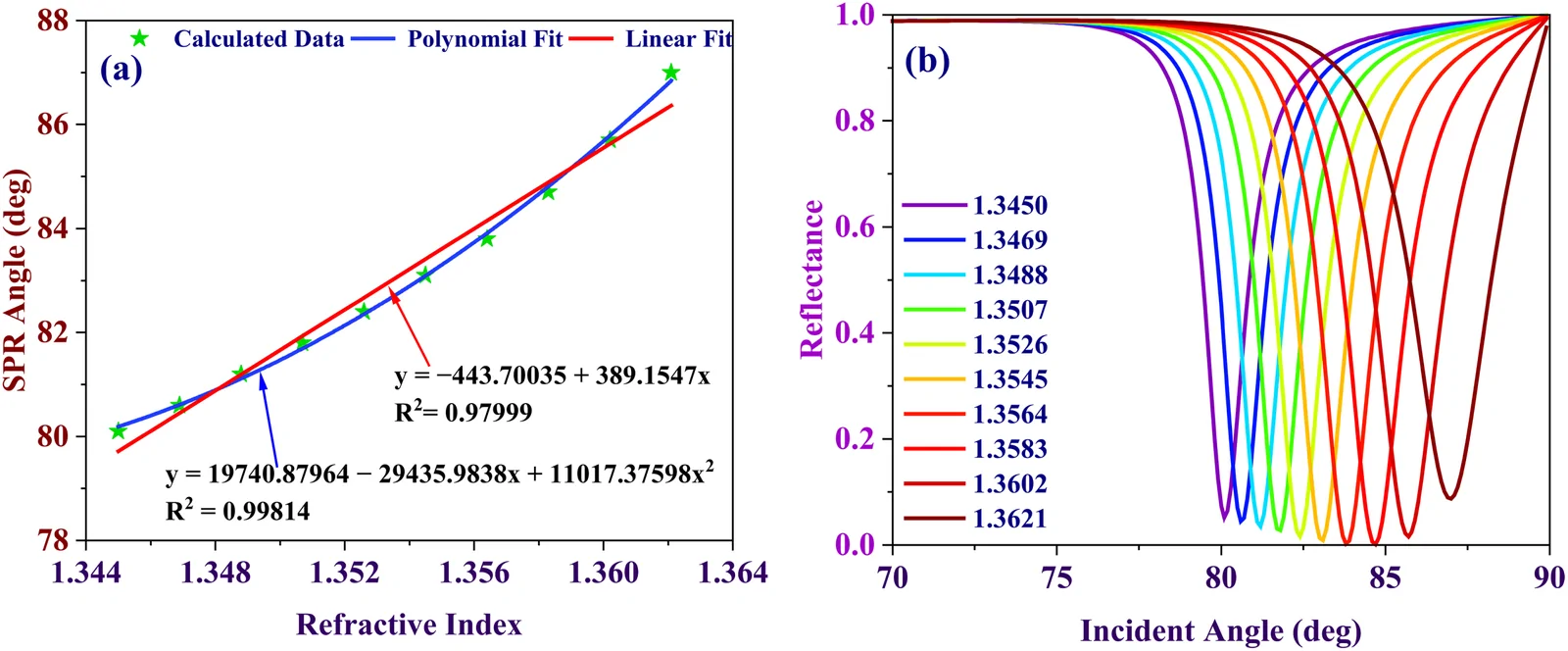
(a) The RI of the corresponding fat in milk is polynomial and linear correlated with the resonance dip angle, and (b) Reflectance vs Incident Angle (deg) for RI 1.3450 to 1.3621.

The relationship between the RI of fat-containing milk samples and the resonance angle of the SPR biosensor is shown in Fig 11(b), using linear and polynomial curve fitting techniques. The RI of the analyte changes from 1.3450 to 1.3621, and the resonance angle changes from 80 deg to 87 deg. The linear correlation between resonance angle and RI is described by the equation 𝑦=−443.70035 + 389.1547𝑥, while the polynomial fit is expressed as 𝑦 = 19740.87964 − 29435.9838𝑥 + 11017.37598𝑥^2^. The coefficients of determination are R^2^ = 0.97999 for the linear relationship and R^2^ = 0.99814 for the polynomial model, indicating a strong agreement between the fitted curves and the observed data. The proximity of the values to unity further establishes the high predictability and reliability of the sensor response. In addition to that, Fig 11(a) shows the variation of reflectance for different incident angles and RI values for varying fat concentration in milk. It can be observed that there is a consistent trend in the shift of the resonance angle to higher incident angles as the RI increases. This demonstrates the high sensitivity and stability of the suggested SPR biosensor, validating its potential for fat content determination in milk based on RI changes.

### 3.11 Feasible fabrication approach and robustness analysis with 10% error margin

**Fig 12:**
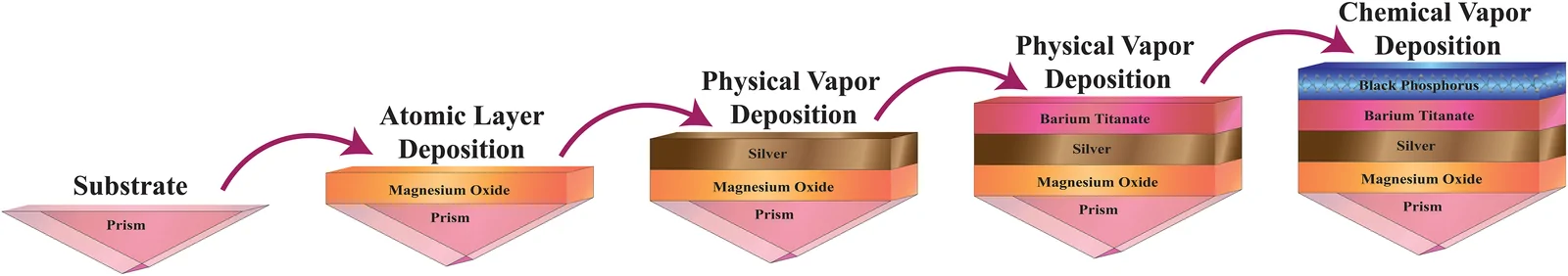
Possible fabrication techniques of the proposed SPR sensor.

The SiO_2_ prism substrate is subjected to a rigorous cleaning process using sequential ultrasonic cleaning in acetone, isopropanol, and deionized water. After cleaning the substrate, it is dried using nitrogen gas and then subjected to a thermal treatment process for the removal of moisture and other impurities [67]. To deposit the MgO layer on the SiO_2_ prism substrate, the ALD process is employed [68]. This process is particularly useful for the deposition of a uniform layer of the MgO material with good control over the thickness of the layer. Finally, the silver layer is deposited using the PVD process over the MgO layer [51]. This process is particularly useful for the deposition of the silver layer with minimal loss and good smoothness of the layer. After this, a high refractive index material of BaTiO_3_ is deposited using a PVD technique to increase the confined electromagnetic field at the sensing interface [69]. The precision control of film thickness by the PVD technique is critical in enhancing the performance of the sensor. Finally, an ultrathin black phosphorus (BP) material is synthesized and coated on the surface of the previously created BaTiO_3_ using a CVD technique [27]. CVD is a technique that allows high-quality BP films to be formed with strong properties of light-matter interaction [70]. The BP material is used to create the active sensing interface. The fabrication of the multilayer structure is complete and ready for functionalization and characterization. The step-by-step fabrication process is shown in Fig 12.

To examine the practical feasibility and robustness of the developed SPR biosensor design, a comprehensive fabrication tolerance analysis was carried out. In this analysis, a systematic change in thickness was considered for all metallic and dielectric layers by varying their values by ±10%. This is a reasonable assumption since these variations can be caused by inaccurate fabrication conditions that can occur during thin-film deposition techniques. In addition to this, a combined tolerance analysis was carried out by varying the thicknesses of all three layers – MgO, Ag, and BaTiO_3_ within a range of ±10%. A summary of changes in all three performance parameters sensitivity, minimum reflectance, and resonance angle change with respect to a combined tolerance analysis is provided in Table 5. From this analysis, it can be concluded that even though a certain degree of change is observed in all three performance parameters, these changes are negligible and minor.

**Table 5:** Influence of ±10% thickness error on the performance metrics of the proposed sensor for RI of 1.3621.

Performance parameter	Error possibility of all layers for +10%	Error possibility of all layers for −10%
Sensitivity (deg/RIU)	443.04	333.56
R_min_	0.752	0.032
FWHM	3.343	2.625
DA (deg^-1^)	0.299	0.381
QF (RIU^-1^)	132.52	127.07
FOM	32.86	122.93

### 3.12 Numerical validation of the proposed SPR sensor

**Fig 13:**
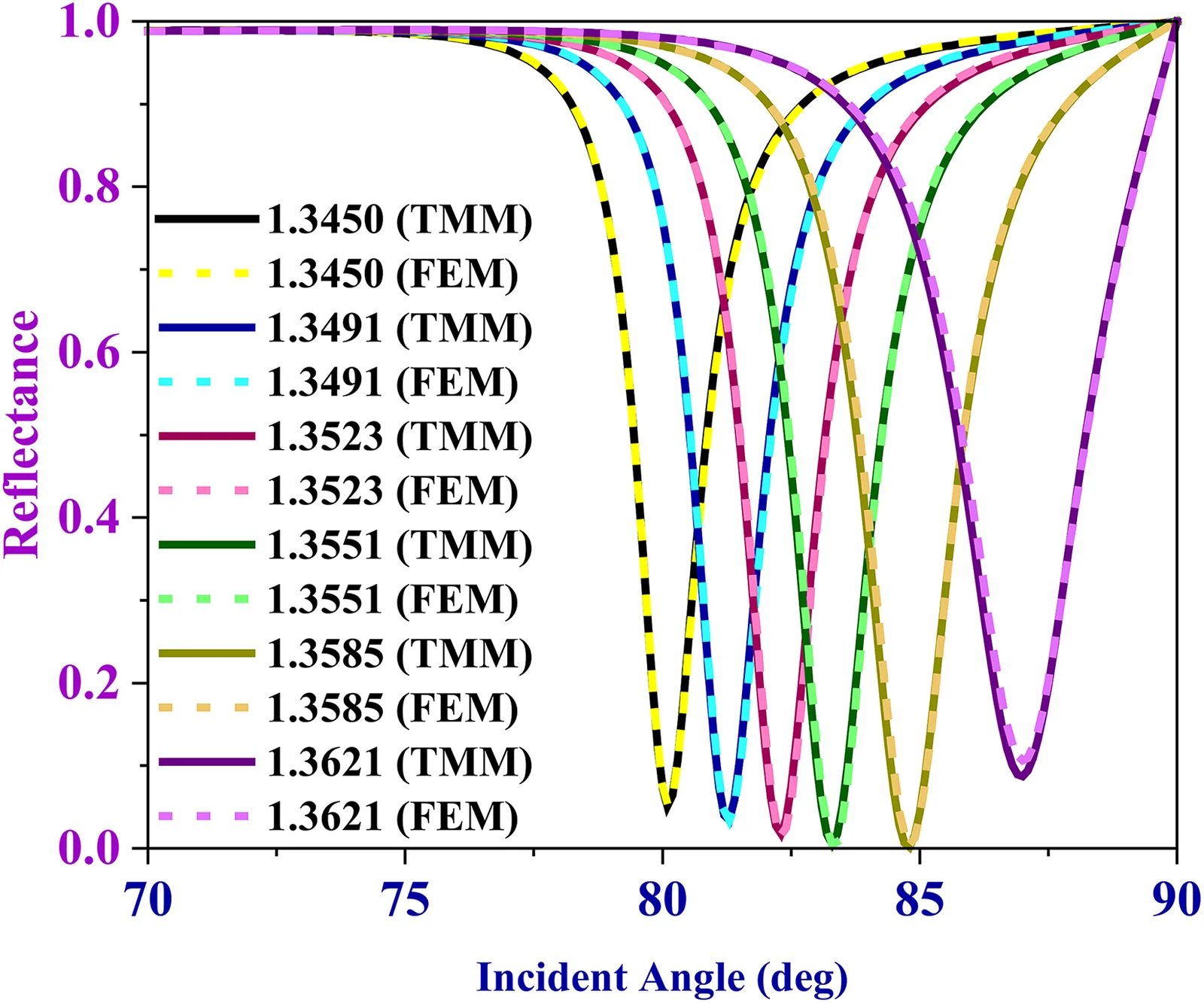
Numerical validation of the proposed SPR sensor: comparison of TMM- and FEM-based reflectance curves.

The validity of the proposed SPR sensor model was established by conducting a comparative analysis of the simulated reflectance spectrum and resonance angle calculated by both TMM (MATLAB) and FEM (COMSOL Multiphysics 6.2) shown in Fig 13. Both models yielded very similar resonance curves with almost perfect matching in the resonance angle and minimum reflectance. The maximum difference in the calculated resonance angle by the two methods was shown to be less than 0.1°, ensuring the consistency and reliability of the used numerical models in terms of optical behavior predictions. In addition, the distribution of the electric field generated by the TMM model was verified by the FEM approach. Both models had a maximum electric field intensity of approximately 2.5 × 10^5^ V/m and a plasmon penetration depth of approximately 200 nm.

### 3.13 Simulation scope, limitations, and future directions

This paper considers the modeling of the design, analysis, and optimization of the SPR sensing device proposed for the detection of milk fat by using the experimental refractive indices of different concentrations of milk fat. The purpose of this research is the investigation of the performance of the designed multilayer SPR structure with respect to the refractive index sensing and the optimal setting of its parameters for getting high sensitivity and narrow resonant spectrum. The validation of the numerical simulation model was performed by means of cross-checking of the results obtained by means of TMM and FEM methods. Despite all these promising results, some restrictions must be mentioned. The current research is totally theoretical and involves simulations only without actual manufacturing and characterization of the designed sensor. Additionally, the sensing principle is based on the variations in refractive index and does not take into account surface functionalization, biomolecular interactions or molecular selectivity. Thus, the obtained results should be treated as the refractive index sensing ability of the suggested SPR-based platform and not as the actual milk fat biosensor. Indeed, the real milk is quite a complicated biological medium which consists of fats, proteins, carbohydrates, minerals, water and other components; hence, some additional studies will be needed to test the sensor performance in the conditions where different physicochemical parameters are changing at the same time. The future works will involve the manufacturing and experimental testing of the suggested sensor using real milk samples. In addition, it is possible to use some selective bio-recognition layers, like lipase, PAA-Chitosan-Lipase and PMMA-Lipase coatings for the triglyceride recognition among others.

## 4. Comparison of the proposed work with previous studies

The performance of the proposed biosensor in terms of SPR response is compared with previously reported works, as depicted in Table 6, and the respective percentage variation is discussed in detail. From this comparative study, it is evident that the sensitivity of the proposed biosensor is significantly improved, achieving a higher value by 18.3%, 12.2%, 79.0%, 46.7%, and 34.8% compared to ref-1, ref-2, ref-3, ref-4, and ref-5, respectively. Similarly, the QF of the proposed biosensor is significantly improved, achieving a higher value by 42.7%, 28.6%, 374.9%, and 88.8% compared to ref-1, ref-2, ref-3, and ref-5, respectively. Similarly, in terms of DA, although lower values are observed for the proposed biosensor compared to ref-1 and ref-2, significant improvements are achieved by 166% and 98% compared to ref-3 and ref-4, respectively. Overall, it is evident from the comparative analysis provided in Table 6 that this proposed biosensor has better performance in terms of certain parameters, such as sensitivity and QF, thus proving it to be a very efficient and advanced biosensor.

**Table 6:** Comparison between the present work and previous studies.

Ref work	Application	Sensor configuration	Sensitivity (deg/RIU)	QF (RIU^-1^)	DA (Deg^-1^)	Ref.
This work	Milk Fat	SiO_2_ + MgO + Ag + BaTiO_3_ + BP (Iterative) SiO_2_ + MgO + Ag + BaTiO_3_ + BP (ML)	401.40 424.61	148.33 139.03	0.369 0.327	– –
Ref-1	Milk Fat	BK7 + Ag + AgGaS_2_ + LiGaS_2_ + BlueP/WSe_2_	359.00	97.42	1.66	[16]
Ref-2	TB	BK7 + Ag + Tl3AsSe3 + BP	378.36	108.10	1.848	[41]
Ref-3	Milk Adulteration	BK7 + Cr + Au + AgBP/WS_2_	237.20	29.27	0.123	[71]
Ref-4	Acetone	BaF_2_ + ZnO + Cu + Si + MXene	289.40	–	0.165	[72]
Ref-5	Fluorides	CaF_2_/P3HT:PC61BM/Ag	315.10	73.63	–	[73]

## 5. Conclusion

In the present work, a highly sensitive and efficient multilayer SPR biosensor has been successfully developed for the detection of fat content in milk by employing the angular interrogation method. The proposed SPR sensor, which is based on the optimized Kretschmann configuration of the SiO_2_/MgO/Ag/BaTiO_3_/BP multilayer, offers superior sensing performance by virtue of the improved confining of the electromagnetic field and the intensified plasmonic effects. The combined effects of the high RI dielectric materials and the ultra-thin BP film are seen to play a crucial role in enhancing the light-matter interactions and the penetration depth of the evanescent field, thereby offering superior sensing performance. The accuracy and reliability of the proposed SPR design are validated by employing TMM, FEM, and FDTD approaches, which show good agreement among the results obtained by the three approaches. To ensure the best sensing capability, the thickness parameters of the multilayer are optimized using a combination of iterative and brute-force optimization methods, allowing the determination of the arrangement that yields the lowest reflectance and improved sensitivity. The sensor exhibits a remarkably high sensitivity of 401.40 deg/RIU at a RI of 1.3621, along with a low minimum reflectance of 0.086, a high-quality factor of 148.33 RIU^-1^, and a detection accuracy of 0.3695 deg^-1^. Moreover, the linear shift in the angle of resonance is a proof of the high sensitivity, stability, and reliability of the sensor. The proposed biosensor offers a rapid, label-free, and highly precise method for detecting fat content in milk, which is critical for ensuring the quality of milk and dairy products, detecting adulteration, and maintaining regulatory standards. Due to its reliability and high-performance characteristics, the proposed SPR biosensor possesses immense potential for being implemented for various applications in dairy quality control and food safety monitoring.
