# Supplementary material for: Hybrid machine learning driven optimization of multilayer SPR sensor for high sensitivity milk fat detection
Source: PLoS One. 2026 Sep 2;21(9):e0355996. doi: 10.1371/journal.pone.0355996 (PMC13537604; doi:10.1371/journal.pone.0355996)
Supplement: Supplementary file 1 [file pone.0355996.s001.pdf]

Reviewer Report: Numerical and Theoretical Assessment of the Manuscript on Hybrid Machine Learning Driven Optimization of a Multilayer SPR Sensor for Milk Fat Detection

Overall Recommendation

This manuscript presents a simulation-based multilayer SPR sensor for milk fat detection using a SiO2/MgO/Ag/$BaTiO_3$/BP architecture and reports a maximum angular sensitivity of 401.40 deg/RIU at 633 nm. The topic is relevant and the authors attempt a relatively broad numerical framework by combining transfer matrix method (TMM), finite element method (FEM), and finite-difference time-domain (FDTD) simulations with a brute-force optimization strategy. However, the current version has substantial methodological and theoretical weaknesses that prevent confidence in the reported performance claims. The most important issues concern numerical inconsistency in the optimization logic, insufficient disclosure of the optimization workflow, weak physical justification for the chosen objective criteria, inadequate treatment of the analyte as a real milk system, and the lack of simulation studies that test the robustness of the model under realistic perturbations. On this basis, the manuscript requires **minor revision**.

Major Concerns

1. Numerical inconsistency in the brute-force optimization framework

The manuscript claims a hybrid optimization approach (brute-force, machine learning, iterative refinement), but the evidence shown relies mainly on one-parameter-at-a-time thickness sweeps. This creates a mismatch: a true brute-force method should explore the full multidimensional parameter space and report details like search range, step size, total configurations, and layer interactions. If the method is actually sequential, it cannot be considered global optimization, since layer properties in multilayer SPR systems are interdependent. Optimizing layers independently ignores cross-coupling effects that influence resonance behavior. While sequential sweeps can provide insight, they should not be presented as equivalent to a full multidimensional optimization.

2. Unclear objective function and post-hoc selection of “optimal” thickness

The manuscript claims optimization for maximum sensitivity, but the chosen design does not always correspond to the highest sensitivity values. Instead, selections are based on trade-offs with

factors like minimum reflectance and resonance quality. This indicates a multi-objective optimization problem, not a single-objective one, and it should be clearly presented as such. Additionally, the use of a fixed reflectance threshold ($R_{min} < 0.1$) appears arbitrary and lacks proper justification. A more rigorous approach would define a clear merit function or use multi-objective (e.g., Pareto) optimization to balance sensitivity and resonance characteristics.

3. Claimed hybrid ML optimization is not methodologically established

The manuscript claims a hybrid optimization approach involving machine learning, but provides no meaningful details about the ML model, such as architecture, data, training process, or validation. The mention of simple tools like correlation and linear regression is insufficient to support a true ML-based framework. If ML was only used for basic trend analysis, the novelty claim is overstated. If it played a real role in optimization, the paper should present clear evidence showing how it improves efficiency or accuracy compared to brute-force methods. As written, the "hybrid ML-driven optimization" claim appears overstated relative to the methodological documentation available in the manuscript.

4. Missing cross-method numerical consistency metrics

The manuscript claims strong agreement between TMM, FEM, and FDTD, but does not provide a clear quantitative comparison of results (e.g., resonance angle, reflectance, sensitivity) across the methods. Since this agreement is a key validation point, the paper should include explicit comparisons with error metrics. Simply stating "outstanding agreement" without numerical evidence or uncertainty analysis is insufficient.

5. Convergence reporting is not yet adequate for a high-precision claim

The manuscript reports very fine mesh/grid settings and strict tolerances, but does not provide sufficient evidence of numerical convergence or stability. Key elements like mesh/grid convergence studies, time-window validation (for FDTD), and sensitivity to simulation parameters are missing. Without uncertainty analysis, it is unclear whether the reported performance improvements are numerically robust, especially since the gains over prior work are relatively modest.

6. The analyte model is physically oversimplified for milk fat sensing

The manuscript models sensing as a simple refractive-index shift, assuming milk fat concentration directly maps to RI. However, milk is a complex, scattering colloidal medium, and its optical response depends on multiple factors beyond RI. Because these complexities are not considered, the study effectively validates only a generic RI sensor, not a realistic milk-fat detection system. The claim should be limited to RI sensing unless a more accurate milk model is included.

7. Biosensor terminology is not fully justified

he manuscript refers to the device as a "biosensor," but the analysis is based only on bulk refractive-index sensing. It does not include key elements of true biosensing, such as surface functionalization, binding kinetics, or selective biorecognition mechanisms. As a result, the optical response is imposed through RI changes rather than a physical interfacial process, making the design more accurately a theoretical RI sensor rather than a demonstrated milk-fat biosensor.

8. Comparison framework is only partly appropriate

The manuscript compares its sensor with prior SPR studies that are not specific to milk. [ref.51], [ref.52]. While general RI-based comparisons are acceptable for optical benchmarking, they do not fully support claims of superiority for milk fat sensing. The paper should clearly separate generic sensor comparisons from application-specific comparisons (e.g., milk or food matrices). Without this distinction, the practical relevance of the comparison is overstated.

9. Lack of experimental validation substantially weakens reliability

The work is entirely simulation-based, with no experimental fabrication, measurements, or validation against standard milk-analysis methods. While simulation studies can be valid, this one lack sufficient physical rigor and clear model limitations. Combined with simplified analyte assumptions and unclear optimization, the results are not experimentally supported. The study should therefore be presented as a theoretical design rather than a validated milk-quality sensing solution.

Minor Issues

- The term "major breakthrough" is too strong for a simulation-only study without experimental validation.
- The manuscript should clearly distinguish "high sensitivity" from "best practical sensor," because high angular sensitivity alone does not guarantee superior detectability.
- The wording around MgO as an "adhesive" and an optical enhancement layer should be clarified, since these are different physical roles.
- The BP layer thickness of 0.53 nm is effectively atomic-scale and needs a stronger fabrication and stability discussion.
- The data-availability statement appears weak for a numerical paper; scripts, parameter files, and simulation settings should be shared if possible.

Additional Simulation Work Recommended

The manuscript would be substantially strengthened by adding the following simulation studies.

1. Perform true multidimensional optimization (or a well-documented surrogate/evolutionary method) instead of sequential sweeps. .
2. Include convergence studies for mesh, grid, and angular steps.
3. Add fabrication-tolerance and uncertainty analysis to test robustness.
4. Use a more realistic model for milk (e.g., effective-medium approach) or limit claims to generic RI sensing.
5. Analyze surface adsorption effects to support the "biosensor" claim.

.**Final Recommendation**

Decision: Minor Revision. The work is interesting and potentially publishable after substantial improvement, but the present version does not yet provide a sufficiently transparent or physically complete basis for the strong claims made about optimization, sensing validity, and practical applicability.
