# Supplementary material for: Hybrid machine learning driven optimization of multilayer SPR sensor for high sensitivity milk fat detection
Source: PLoS One. 2026 Sep 2;21(9):e0355996. doi: 10.1371/journal.pone.0355996 (PMC13537604; doi:10.1371/journal.pone.0355996)
Supplement: Supplementary file 2 [file pone.0355996.s003.pdf]

Subject: Thank You for the Revision Opportunity - Manuscript

Dear **Professor Zaky A. Zaky,**

I would like to sincerely thank you for your valuable guidance and for giving me the opportunity to revise my manuscript entitled *"Hybrid Machine Learning Driven Optimization of Multilayer SPR Sensor for High Sensitivity Milk Fat Detection."*

I deeply appreciate the constructive feedback provided by you and the reviewers. Your comments have been instrumental in identifying key areas for improvement, including section organization, figure and table modifications, abstract and conclusion refinement, and overall presentation quality. I have carefully addressed every point raised and made the necessary revisions to enhance the manuscript's readability, accuracy, and clarity. I also ensured that the data presentation, references, and formatting fully comply with the journal's requirements.

Thank you once again for your and the reviewers time, effort, and thoughtful insights. The revision process has been an enriching experience, and I believe that the manuscript has significantly improved as a result of your valuable feedback.

I look forward to your further assessment of the revised version.

Sincerely,
Md. Al Amin Islam Utshob

Response to Editor

Dear **Professor Zaky A. Zaky,**

On behalf of all co-authors, I would like to express our sincere gratitude for your valuable time and consideration in handling our manuscript entitled *"Hybrid Machine Learning Driven Optimization of Multilayer SPR Sensor for High Sensitivity Milk Fat Detection."* We are also truly grateful to the reviewers for their insightful and constructive comments, which have been invaluable in improving the clarity, rigor, and overall quality of our work.

We have carefully revised the manuscript in accordance with all the comments and suggestions provided by the reviewers and have included a detailed point-by-point response outlining the changes made. We believe that the revisions have strengthened the manuscript considerably.

We sincerely thank you for the valuable comments and suggestions, which have significantly improved the quality of our manuscript. Based on the reviewer's and editor's recommendations, we have thoroughly revised the manuscript. The major revisions are summarized below:

- **Page 1:** Content from **Line 23** has been revised and updated. Additionally, new text has been added in **Lines 42-47**.
- **Page 2: Lines 67-69** have been removed. New content has been incorporated in **Lines 69-70, 72-77, and 92-98**.
- **Page 3:** New content has been added in **Lines 99-114**.
- **Page 4:** New content has been added in **Lines 181-183**.
- **Page 7:** The content from **Line 245** has been revised and updated.
- **Page 10:** New content has been added in **Lines 293-298**.
- **Page 14:** New content has been added in **Lines 385-404**.
- **Page 15: Lines 405-410** have been removed, and new content has been added in **Lines 425-427**.
- **Page 16:** Part of the content in **Lines 445-446** has been removed, and new content has been added in **Lines 438-445**.
- **Page 17:** The caption/title of **Figure 11** has been revised.
- **Page 18:** A new subsection (**Section 3.7**) has been introduced, and new content has been added in **Lines 504-511**.
- **Page 19:** A new subsection (**Section 3.8**) has been added. New content has been incorporated in **Lines 515-531**, and a new **Figure 13** has been included.
- **Page 20:** A new row titled **"Application"** has been added to **Table 6**.

In addition, the manuscript has been carefully proofread, and grammatical, typographical, and language-related errors have been corrected throughout the manuscript to improve its clarity and readability.

As per the reviewer's feedback, new citations have been added at 6, 7, 8, 15, 19, 26, 28, 32-39, 48, 49, 51, 53, 62, 63, 64, and 66.

To make revisions transparent for the reviewers and editor, we have applied the following color-coding in the revised manuscript:

~~Red text~~ → indicates removed lines/content.

Green text → indicates newly added lines/content.

This ensures that all changes are easily traceable.

We believe these revisions significantly improve the clarity, focus, and overall quality of the manuscript. We thank you and the reviewers for your constructive feedback and look forward to your further consideration.

Response to Reviewer #2

Reviewer#2, Concern #1: The idea of combining SPR with machine learning is promising, but the novelty is not clearly established. Similar approaches using ML for optical sensing and classification have already been reported. The authors should clearly highlight what is new in their framework (e.g., dataset, model, feature extraction, or sensing strategy).

Author Response: Thank you for this important remark from the reviewer. We do agree that the novelty of the suggested machine learning hybrid method must be emphasized more clearly.

The novelty of the current study does not consist in the application of machine learning alone, because such ML-supported optical sensing was reported earlier. The innovation of this study consists in the development of a physics-guided hybrid optimization method combining the TMM, MLR, and Brute Force algorithms for designing a multilayer SPR sensor. Contrary to the existing ML-based sensing methodologies that concentrate on the classification or detection of signals using experimental datasets, the novel framework uses TMM-simulated data to train an MLR surrogate model to derive the connection between the multilayer structure parameters and the performance of the sensor. Further, the trained model is coupled with a Multidimensional Brute Force Optimization method to determine the optimum values of MgO, Ag, and $BaTiO_3$ thicknesses. This optimization technique does not require any experimental dataset and also reduces the computational time by eliminating the need for running multiple full-wave simulations. In addition, the proposed framework is used to optimize a BP/$BaTiO_3$ multilayer SPR sensor to detect milk refractive index sensing, achieving an enhancement in the maximum sensitivity from 401.40 deg/RIU to 424.61 deg/RIU. As a means of enhancing the contributions of the paper, we have reviewed the Introduction and Machine Learning Methodology sections.

Reviewer#2, Concern #2: The sensing mechanism itself is not sufficiently explained. It is unclear how variations in milk composition (fat content or adulteration) are translated into refractive index changes and how reliably SPR captures these variations.

Author Response: Thank you for your concern. We agree with the reviewer that there was some need for clarification regarding the sensing mechanism in the original manuscript.

The new manuscript has been extended to include an explanation of the sensing mechanism. The sensing mechanism works on the premise that changes in milk composition, such as a change in fat composition, affect the effective refractive index (RI) of the milk solution. Refractive indices used in our study were not taken arbitrarily; rather, they were taken from earlier experimental results found in literature. A change in the effective RI of the analyte leads to a change in the propagation constant of the surface plasmon wave at the metal-analyte interface, resulting in the disruption of the momentum matching condition necessary for SPR excitation. The result is a change in the resonance angle and this angular shift is measured using the angular interrogation method. In addition, in order to provide further insight into the potential path towards practical implementation, we provided an extended section discussing future work. Specifically, the proposed SPR device can be made into a selective biosensor by adding the biomarker specific biorecognition layers, like lipase, PAA-Chitosan-Lipase or PMMA-Lipase [1], [2], [3]. In the case of such an arrangement, the triglycerides, which are the main biomarkers for milk fat, will be specifically bound by the immobilized lipase layer. As a

result, there will be an interfacial biochemical reaction causing an alteration of the local dielectric environment, leading to an additional refractive index change, detectable as a shift of the SPR resonance angle.

To better specify the scope of the current paper, we also included the explanation that the current device is a refractive-index based sensing platform using experimentally obtained values of refractive index of milk. The quantitative calibration between fat content, bioreactions and SPR signal obtained experimentally will be a subject of our future work. These clarifications have been added to the new manuscript version.

[1] O. Kirk and M. W. Christensen, "Lipases from Candida antarctica: Unique Biocatalysts from a Unique Origin," Org. Process Res. Dev., vol. 6, no. 4, pp. 446–451, Jul. 2002, doi: 10.1021/op0200165.

[2] M. Chakraborty and K. Biswas, "Hardware Platform to Detect Fat Percent in Milk Using a Lipase Immobilized PMMA-Coated Sensor," IEEE Trans. Instrum. Meas., vol. 68, no. 11, pp. 4526–4534, 2019, doi: 10.1109/TIM.2018.2890320.

[3] S. Zhou, X. Li, J. Zhang, H. Yuan, X. Hong, and Y. Chen, "Dual-fiber optic bioprobe system for triglyceride detection using surface plasmon resonance sensing and lipase-immobilized magnetic bead hydrolysis," Biosens. Bioelectron., vol. 196, p. 113723, Jan. 2022, doi: 10.1016/J.BIOS.2021.113723.

Reviewer#2, Concern #3: A major concern is the source of refractive index values used for milk or fat variations. The manuscript does not clearly mention whether these values are taken from experimental data, literature, or assumed. Proper references or justification are required.

Author Response: We sincerely acknowledge the insightful feedback. It is important to state that all the results shown in the paper are based on computer simulation and do not involve any experimental data acquisition process. The values of the refractive index (RI) from 1.345 to 1.3621 were used in accordance with previous publications where experimental correlations between RI and composition changes in milk, including its fat content, have been established [4], [5]. Suitable literature references have been provided in the revised version of the manuscript. Additionally, it has been specified in the manuscript that variations of the RI due to the effect of temperature change were not taken into account in the present simulations since the aim was to assess the sensor intrinsic performance under controlled conditions. Moreover, it has been noted that the effect of temperature and other constituents, including proteins, lactose, and minerals in addition to fat, might also affect the RI in practical milk samples.

[4] A. J. Jääskeläinen, K. E. Peiponen, and J. A. Räty, "On reflectometric measurement of a refractive index of milk," J. Dairy Sci., vol. 84, no. 1, pp. 38–43, 2001, doi: 10.3168/jds.S0022-0302(01)74449-9.

[5] B. Karki, Y. Trabelsi, A. Uniyal, A. Pal, and R. Bharos Yadav, "Detection of fat concentration milk using TMDC-based surface plasmon resonance sensor," Modern Physics Letters B, vol. 38, no. 29, Oct. 2024, doi: 10.1142/S0217984924502531.

Revised text (Page 2, Line 92-98): "The detection principle behind the suggested SPR sensor is based on angular interrogation. The alterations in the milk's components composition, such as alterations in the fat component or in its adulteration, alter the effective refractive index of the medium, as shown by experimental studies [32]. Such alterations in the effective refractive index change the momentum matching required for exciting surface plasmons in the metal-analyte interface, thereby causing an alteration in the SPR resonance angle.

Consequently, the suggested sensor indirectly senses any alterations in milk's components composition through the alteration in its refractive index."

Reviewer#2, Concern #4: The manuscript does not address whether similar refractive index variations could arise from other components present in milk (e.g., proteins, lactose, added water, or other adulterants). This raises concerns regarding selectivity and specificity.

Author Response: We are truly grateful to the reviewer for this important comment. In our opinion, the bulk refractive index change in milk could not occur only due to the change in fat concentration in milk but may also be a result of some other ingredients such as protein, lactose, added water, etc. Hence, a simple SPR sensor based on refractive index measurement is not able to identify the cause of the refractive index change.

The main aim of the present study is the development and optimization of an SPR refractive index sensing model based on experimentally measured refractive index values for different concentrations of milk fat. In order to overcome this problem, the revised manuscript now clearly indicates that this proposed sensor can currently be considered as a refractive index sensing system rather than a specific milk fat biosensor. Also, the future work section has been updated to indicate that the sensing specificity can be greatly improved by using a biorecognition layer for a specific biomarker. Immobilization of lipase, PAA-Chitosan-Lipase or PMMA-Lipase functional layers which can selectively identify and catalyze the hydrolysis of triglycerides, which is the primary biomarker of milk fat, will produce localized refractive index change in response to the sensing of fat, thereby distinguishing from the refractive index change caused by other milk compounds or adulterants. Also, future experimental work will involve evaluation of the performance of this sensor when used with real milk samples having various compositions along with adulterants.

Reviewer#2, Concern #5: The role of machine learning is not deeply justified. It is not clear, What exact features are used (SPR angle, reflectance curve, etc.), also Why ML is necessary instead of direct analytical modelling and further, Whether the dataset size is sufficient for reliable training.

Author Response: Thank you very much for the valuable comments. In our opinion, the role of the machine learning (ML) framework in the manuscript could be explained in more detail.

Now, in the revised version of the manuscript, it is clearly mentioned that the ML framework does not replace the Transfer Matrix Method (TMM) framework, which is used as the physical-based forward model to calculate the optical properties of the multilayer surface plasmon resonance (SPR) sensors, whereas the ML framework is used as the surrogate modeling tool in order to explore the multidimensional design space during optimization. The simulation data set for the training of the ML model has been generated fully with the help of the TMM framework. The input variables to the ML model are the structural parameters of the sensor, i.e., thicknesses of the MgO, Ag, and $BaTiO_3$ layers. The output or the target variables are the values of the performance parameters of the sensors, i.e., sensitivity, Rmin, FWHM, Q-factor, DA, and FOM. Motivation behind the use of ML is the increase in computational complexity

in case of the optimization directly based on the simulation by TMM. In spite of the fact that TMM predicts the optical response precisely, many computations of thousands of designs become more difficult in such case. After the development of ML model predicting the performance of sensors for each combination of layers thicknesses, a multidimensional Brute Force search is used to obtain the optimal combination of layer thicknesses. Thus, the use of ML increases computational efficiency of the optimization process whereas the design of sensor itself is based on the physical TMM simulations.

In regards to the number of data samples in the dataset, 726 samples have been generated by TMM, out of which 80% was used for the training of the model, and 20% for the testing. The adopted algorithm, Multiple Linear Regression (MLR) does not require big number of data samples due to its relative simplicity and few numbers of input features. For this reason, the dataset of this size is enough to establish the dependencies between structural parameters and sensing properties. The regression equations and Mean Square Error (MSE) values are provided.

Revised text (Page 14, Line 385-404): "For angular interrogation SPR sensors, an intensive resonance dip is critical for determining the resonance angle correctly. The smaller R_{min} implies better coupling of the light from the beam into surface plasmons and, thus, the higher plasmon excitation and better resonance contrast. Therefore, one could consider $R_{min} < 0.1$ as a practical criterion for achieving high enough resonance dip while optimizing the sensor design. The method ensures an optimal solution by avoiding local minima. In order to determine the relationship between structural parameters and performance of sensors, the Multiple Linear Regression (MLR) model was utilized. Simulation dataset was created based on Transfer Matrix Method (TMM) by changing thicknesses of MgO (0-20 nm), Ag (30-60 nm), and $BaTiO_3$ (1-5 nm) layers, while fixing the thickness of silicon layer to be 0.53 nm. Thus, 726 samples were obtained, where the input features were thicknesses of MgO, Ag, and $BaTiO_3$ layers, and output features were sensitivity, Rmin, FWHM, Q, DA, and FOM. Training data constituted 80% of all available dataset, while testing data were the remaining 20%. The performance of regression model for prediction was determined via Root Mean Square Error (RMSE), Mean Absolute Error (MAE) and coefficient of determination (R^2). Trained regression model was further incorporated into Brute Force optimization algorithm to find the optimal set of layer thicknesses meeting the requirements of design process. The Multiple Linear Regression (MLR) analysis was employed on the data set created by TMM in order to formulate the mathematical relation between the structural parameters and sensor performance. The input parameters were made up of the thickness of MgO, Ag, and $BaTiO_3$ layers, while the response variables were sensitivity, R_{min}, FWHM, QF, DA, and FOM. In comparison to traditional optimization based on sweeping through values of the parameters resulting in maximum sensitivity of 401.40 deg/RIU, the proposed hybrid ML-assisted optimization resulted in increased sensitivity of 424.61 deg/RIU and improved sensing performance."

Reviewer#2, Concern #6: There is no clear discussion of dataset preparation such as How many samples were used, Whether the data is simulated or experimental, How the data is split (training/testing).

Author Response: We would like to sincerely appreciate the reviewer for this constructive feedback. We acknowledge the fact that the dataset creation process should have been elaborated better.

In the updated version of our manuscript, we have extended the section describing the machine learning methodology, where the process of dataset creation is described. The dataset used in this study is completely simulation based and has been created by changing the thicknesses of the MgO, Ag, and $BaTiO_3$ layers within their respective ranges through Transfer Matrix

Method (TMM). For each of the structural configurations, the sensing properties have been calculated using sensitivity, Rmin, FWHM, Q-factor, DA, and FOM.

The total number of data samples is 726, and the thicknesses of the layers form the input features while the calculated sensing properties form the output. For model performance evaluation purposes, the dataset has been split into 80% training data and 20% testing data. Since the data samples have been created through physics-based Transfer Matrix Method, all the training samples are physically meaningful.

Reviewer#2, Concern #7: How sensitive is the model to noise?

Author Response: We appreciate the reviewer for making such a valuable comment. We admit that the robustness of machine learning to the presence of noise in the input data is important. However, in our current research, the dataset was created exclusively based on the physics-driven Transfer Matrix Method (TMM). As a result, the training data are deterministic and have no experimental noise and uncertainties. Therefore, the noise sensitivity analysis was not done as it was not included in the scope of the current research. The selected Multiple Linear Regression (MLR) model is rather simple and has low complexity, which means that it learns the global dependence of the parameters and the characteristics, and therefore, it is less prone to overfitting compared to other ML models in case of physics-based data. We agree with the reviewer that the analysis of robustness to noise will increase the practicability of the developed framework. In this way, we have incorporated this point in the revised version of the manuscript in the section of future work.

Reviewer#2, Concern #8: Will small measurement errors affect classification?

Author Response: We would like to express our gratitude to the reviewer for providing such an important remark. To be more precise, we would like to emphasize that the key goal of the current study is to optimize sensors, but not to perform classification of samples. That is why the machine learning algorithm under development is applied for prediction of the sensor sensitivity depending on various parameters of multilayer SPR structures.

In addition, we should note that, since the training dataset was created via the deterministic method of TMM, it did not have any measurement errors. That is why we did not consider the impact of small errors on classification results in the course of the current research. Based on the sensing approach, any small change in the resonance angle should cause a slight uncertainty in the estimation of the refractive index, as long as the resolution of the measurements is high enough. Additionally, the sensor under development shows a strong resonance dip, characterized by high sensitivity and high detection accuracy, thereby making it possible to detect small changes in the resonance angle. However, we understand that some experimental errors in measurements might affect sensing performance. In order to overcome this weakness, we have modified the manuscript such that future research will involve experimental evaluation of the sensor using actual milk samples, along with measurement uncertainty, noise resistance, and the performance of machine learning algorithms for classification of biomarker-assisted functionalized surfaces.

Reviewer#2, Concern #9: From the SPR side, the analysis is somewhat limited. The paper would benefit if authors mention the Clear resonance curve analysis, Shift explanation with changing refractive index and Physical interpretation of sensing behavior.

Author Response: We thank the reviewer for this helpful comment. To address this comment, we have updated our discussion in the revised manuscript to better explain the characteristics of the resonance curves along with the working principle of the SPR device. In this context, it has been discussed that the reason behind the prominent resonance dip observed in the reflectance curve is that there is a strong coupling between the incident p-polarized light beam and the surface plasmon polaritons (SPPs) present at the interface of the metal and dielectric medium [6]. At the resonant angle, momentum of the photons of the incident light is matched with that of the surface plasmons and consequently, the energy transfer is maximized giving rise to the minimum in the reflected light intensity.

Moreover, we have made it clear that the increase in the refractive index of the milk solution leads to the change in the propagation constant of the surface plasmon wave, thus leading to the matching of momenta at a higher value of the incident angle. Consequently, the resonance angle in the reflectance curve gets shifted towards right and the amount of this shift is directly proportional to the refractive index of the analyte [7]. In order to further augment the physical significance of the proposed design, it has also been explained that due to better electromagnetic field confinement by the optimized multilayer configuration, a sharper resonance dip with reduced reflectivity and increased sensitivity is observed, making the detection of slight variations in the refractive index possible. Furthermore, it has been highlighted that in order to implement the proposed design in a practical application, specific biorecognition layers based on the nature of the biomarker (lipase, PAA-Chitosan-Lipase, or PMMA-Lipase) could be utilized to react with the triglycerides, the primary biomarker of milk fat.

[6] M. A. Butt, "Surface Plasmon Resonance-Based Biodetection Systems: Principles, Progress and Applications—A Comprehensive Review," Jan. 01, 2025, Multidisciplinary Digital Publishing Institute (MDPI). doi: 10.3390/bios15010035.

[7] J. Homola, "Surface Plasmon Resonance Sensors for Detection of Chemical and Biological Species," Chem. Rev., vol. 108, no. 2, pp. 462–493, Feb. 2008, doi: 10.1021/cr068107d.

Revised text (Page 3, Line 100-114): "In future experiments, the sensitivity of the detection system may be improved by functionalizing the biosensor surface using biorecognition layers like lipase, PAA-Chitosan-Lipase, or PMMA-Lipase. Lipase enzyme acts on the triglycerides in milk fat and cleaves them into glycerol and fatty acids. In addition to generating the biochemical reaction, there is a corresponding change in the local refractive index, which causes a shift in SPR resonance, thus enabling selective detection of milk fat [33]. Immobilization of enzymes using polymer assistance, such as in PAA-Chitosan or PMMA matrices, enhances the enzyme immobilization, reusability, and selectivity of biosensor systems [34], [35]. The recent SPR studies conducted with regard to the sensing of the milk fat quality have shown some promising results when using different combinations of materials and structural arrangements. Nevertheless, the study presented in [36] was mostly concerned with the biosensing performance without looking into structural optimization in detail. At the same time, the study in [37] focused on the development of increased sensitivity due to the use of material engineering. The same can be noted about the works presented in [38], [39]. However, no mathematical model for the fast sensor design was built in the course of these experiments. In its turn, this work uses the approach of multilayer structure optimization together with the predictive MLR model for fast performance estimation."

Reviewer#2, Concern #10: The practical applicability is not fully convincing. It is unclear how the proposed system would work in real milk samples where multiple variables change simultaneously.

Author Response: We are extremely grateful to the reviewer for his/her constructive and insightful comments. We absolutely agree with the reviewer that real milk is an extremely complex biological system with a lot of factors affecting its optical properties, such as fat, proteins, lactose, minerals, amount of water, temperature, and potential adulterants.

In the current study, the aim is to design and optimize an SPR refractive index sensing platform based on numerical simulations, rather than to create a comprehensive milk analytical method for separate quantification of all components of milk. Therefore, we use the experimental refractive indices for numerical analysis of sensing capabilities of our proposed SPR platform. The practical application of the suggested sensor would require initial calibration based on milk samples of known composition and results measured via traditional analytical techniques. Such calibration will define the relationship between the SPR resonance shift and the changes in the parameters characterizing milk quality. Additionally, the selectivity of the suggested sensor can be drastically increased owing to the functionalization of its surface with a specific biorecognition layer that selectively reacts to biomarkers, e.g. lipase, PAA-Chitosan-lipase or PMMA-lipase, responsible for the detection of triglycerides, which is the primary biomarker of the milk fat content. Moreover, temperature control can be used during the measurements, and more sophisticated techniques of data analysis like machine learning and chemometrics can be used to account for the effect of several variables at once.

Reviewer#2, Concern #11: Manuscript requires a comparison table also in this field with the previous relevant published data.

Author Response: We sincerely thank the reviewer for this helpful suggestion. As to the manuscript, please be informed that a comparative study is already included in it between the current work and other studies involving sensing milk fat/other milk adulterations using SPR techniques. It is shown in Table 6 where the performance of the suggested $SiO_2/MgO/Ag/BaTiO_3/BP$ SPR sensor was compared to those of other sensor structures. This comparison reveals that the suggested design is competitive and even superior compared with other devices especially with respect to sensitivity and quality factor.

Table 6. Comparison between the present work and previous studies

Ref work	Application	Sensor configuration	Sensitivity (deg/RIU)	QF (RIU^{-1})	DA (Deg^{-1})	Ref.
This work	Milk Fat	SiO_2+MgO+Ag+$BaTiO_3$+BP (Iterative)	401.40	148.33	0.369	-
		SiO_2+MgO+Ag+$BaTiO_3$+BP (ML)	424.61	139.03	0.327	
Ref-1	Milk Fat	BK7+Ag+$AgGaS_2$+$LiGaS_2$+BlueP/WSe_2	359.00	97.42	1.66	[16]
Ref-2	TB	BK7+Ag+Tl3AsSe3+BP	378.36	108.10	1.848	[41]
Ref-3	Milk Adulteration	BK7+Cr+Au+AgBP/WS_2	237.20	29.27	0.123	[71]
Ref-4	Acetone	BaF_2+ZnO+Cu+Si+MXene	289.40	-	0.165	[72]
Ref-5	Fluorides	CaF_2/P3HT:PC61BM/Ag	315.10	73.63	-	[73]

Response to Reviewer #3

Reviewer#3, Concern #1: The manuscript claims a hybrid optimization approach (brute-force, machine learning, iterative refinement), but the evidence shown relies mainly on one-parameter-at-a-time thickness sweeps. This creates a mismatch: a true brute-force method should explore the full multidimensional parameter space and report details like search range, step size, total configurations, and layer interactions. If the method is actually sequential, it cannot be considered global optimization, since layer properties in multilayer SPR systems are interdependent. Optimizing layers independently ignores cross-coupling effects that influence resonance behavior. While sequential sweeps can provide insight, they should not be presented as equivalent to a full multidimensional optimization.

Author Response: Thank you very much for this concern. As the reviewer noted, the sequential parameter thickness sweeps described in the original paper cannot be treated as multidimensional brute-force optimization, as the optical properties of multilayer surface plasmon resonance systems depend on the coupled effect of all design parameters. Sequential parameter sweeps (one-factor-at-a-time analyses) can be effectively used to study the impact of design parameters. Nevertheless, they do not always ensure the optimum solution, as parameter interactions are neglected.

In order to solve this issue, we modified the text to make a clear distinction between sequential parameter sweeps and the suggested hybrid optimization method. Sequential thickness sweeps were used only for analysis of the individual effect of layer thicknesses on sensing properties as well as generation of a simulation database using the Transfer Matrix Method (TMM). The obtained dataset was used to train a Multiple Linear Regression (MLR) model that finds the dependence between the structural parameters and the sensing performance of the device. Then, the regression model was incorporated into a Brute Force optimization approach that allowed searching the multidimensional design space and optimizing simultaneously the thicknesses of the MgO, Ag, and $BaTiO_3$ layers. Therefore, the obtained optimal structure takes into account the influence of all layers at once instead of optimizing layers separately. In comparison with the traditional approach of the sequential parameter sweep, the proposed approach allowed increasing the sensitivity up to 424.61 deg/RIU and improving the detection accuracy, quality factor, and figure of merit at the same time. In order to avoid any misunderstanding, the flow of the optimization procedure has been modified and clearly explained in the manuscript. The terminology has been also clarified to show that the parameter sweeps were utilized for dataset generation and physical interpretation, while the optimization itself was done using the proposed ML-assisted multidimensional optimization approach.

Reviewer#3, Concern #2: The manuscript claims optimization for maximum sensitivity, but the chosen design does not always correspond to the highest sensitivity values. Instead, selections are based on trade-offs with factors like minimum reflectance and resonance quality. This indicates a multi-objective optimization problem, not a single-objective one, and it should be clearly presented as such. Additionally, the use of a fixed reflectance threshold (Rmin < 0.1) appears arbitrary and lacks proper justification. A more rigorous approach would define a clear merit function or use multiobjective (e.g., Pareto) optimization to balance sensitivity and resonance characteristics.

Author Response: We greatly appreciate this useful comment from the reviewer. We fully agree with the statement that the optimization procedure should be referred to as multi-criteria optimization and not a pure single objective sensitivity optimization. In the revised version of our manuscript, we have added the information that the final configuration of the sensors was not chosen by simply having the maximum angular sensitivity. The optimization took into consideration a compromise between various sensing parameters, such as sensitivity, minimum reflectance (Rmin), full width at half maximum (FWHM), quality factor (Q-factor), detection accuracy (DA), figure of merit (FOM), and the feasibility of having certain thicknesses of the layers.

Despite the fact that some configurations showed higher sensitivity, they demonstrated worse resonant properties with larger Rmin and FWHM, which decreased the efficiency of excitation of surface plasmons, reduced the localization of the resonance and made it more complicated to determine the resonance angle. Thus, they were not chosen as the final design even though they had higher sensitivity. With respect to the condition Rmin < 0.1, it should be noted that this limit was set as a rule for designing a sensor rather than an obligatory condition dictated theoretically. For the SPR sensors with angular interrogation of the surface plasmon resonance, a good coupling between the incoming light and the surface plasmons leads to formation of a deep resonance dip characterized by low reflectivity [7], [8]. The lower value of minimum reflectivity means better plasmon excitation and consequently higher energy transfer from the optical field to the surface plasmon mode, providing a resonance dip that can be reliably measured. Therefore, maintaining the Rmin < 0.1 provides a good balance between sensitivity and resonance dip depth, allowing one to get a clear resonance angle. Resonance-quality considerations are quite common in the past researches on SPR sensors, when both sensitivity and quality of resonances are optimized simultaneously rather than separately.

In order to remove any possible misunderstanding, the manuscript was edited to indicate that the proposed design uses a multi-criteria approach, where the final sensor parameters are chosen depending on both sensitivity and resonance quality.

[7] J. Homola, “Surface Plasmon Resonance Sensors for Detection of Chemical and Biological Species,” Chem. Rev., vol. 108, no. 2, pp. 462–493, Feb. 2008, doi: 10.1021/cr068107d.

[8] J. Homola and M. Piliarik, “Surface Plasmon Resonance (SPR) Sensors,” in Surface Plasmon Resonance Based Sensors, J. Homola, Ed., Berlin, Heidelberg: Springer Berlin Heidelberg, 2006, pp. 45–67. doi: 10.1007/5346_014.

Revised text (Page 14, Line 385-404): “For angular interrogation SPR sensors, an intensive resonance dip is critical for determining the resonance angle correctly. The smaller R_{min} implies better coupling of the light from the beam into surface plasmons and, thus, the higher plasmon excitation and better resonance contrast. Therefore, one could consider $R_{min} < 0.1$ as a practical criterion for achieving high enough resonance dip while optimizing the sensor design. The method ensures an optimal solution by avoiding local minima. In order to

determine the relationship between structural parameters and performance of sensors, the Multiple Linear Regression (MLR) model was utilized."

Reviewer#3, Concern #3: The manuscript claims a hybrid optimization approach involving machine learning, but provides no meaningful details about the ML model, such as architecture, data, training process, or validation. The mention of simple tools like correlation and linear regression is insufficient to support a true ML-based framework. If ML was only used for basic trend analysis, the novelty claim is overstated. If it played a real role in optimization, the paper should present clear evidence showing how it improves efficiency or accuracy compared to brute-force methods. As written, the "hybrid ML-driven optimization" claim appears overstated relative to the methodological documentation available in the manuscript.

Author Response: We are grateful to the reviewer for the above comment. We agree with the reviewer regarding the need for explicit elaboration on the role played by the machine learning model to prevent overstatement of the contribution of the proposed framework.

In response to the above suggestion, we have revised the section "Machine Learning Methodology" in our manuscript. It is now stated clearly that the proposed hybrid optimization methodology incorporates the Transfer Matrix Method (TMM), Multiple Linear Regression (MLR), and Brute Force optimization. Initially, the TMM was simulated to produce a dataset of 726 samples, where the inputs consisted of the thicknesses of the MgO, Ag, and $BaTiO_3$ layers, while the outputs were related to the sensing performance parameters such as sensitivity, minimum reflectance, full width at half maximum (FWHM), quality factor (Q-factor), detection accuracy, and figure of merit. The dataset was divided into 80% training set and 20% testing set. The prediction accuracy of the regression model was measured using MSE values. It is important to note that the MLR model was not only used for trend analysis but was employed as a surrogate predictive model, which evaluates the performance of the sensors under different layer thicknesses combinations. The predictive ability of the MLR was further incorporated into the Brute Force approach, providing an efficient way to find the optimal multilayer structure in the multidimensional design space. Compared to the traditional parameter sweep approach, the proposed hybrid approach led to an increase in sensitivity from 401.40 deg/RIU to 424.61 deg/RIU, along with the improvement in other sensor characteristics.

Revised text (Page 14, Line 385-404): "For angular interrogation SPR sensors, an intensive resonance dip is critical for determining the resonance angle correctly. The smaller R_{min} implies better coupling of the light from the beam into surface plasmons and, thus, the higher plasmon excitation and better resonance contrast. Therefore, one could consider $R_{min} < 0.1$ as a practical criterion for achieving high enough resonance dip while optimizing the sensor design. The method ensures an optimal solution by avoiding local minima. In order to determine the relationship between structural parameters and performance of sensors, the Multiple Linear Regression (MLR) model was utilized. Simulation dataset was created based on Transfer Matrix Method (TMM) by changing thicknesses of MgO (0-20 nm), Ag (30-60 nm), and $BaTiO_3$ (1-5 nm) layers, while fixing the thickness of silicon layer to be 0.53 nm. Thus, 726 samples were obtained, where the input features were thicknesses of MgO, Ag, and $BaTiO_3$ layers, and output features were sensitivity, Rmin, FWHM, Q, DA, and FOM. Training data constituted 80% of all available dataset, while testing data were the remaining 20%. The performance of regression model for prediction was determined via Root Mean Square Error (RMSE), Mean Absolute Error (MAE) and coefficient of determination (R^2). Trained regression model was further incorporated into Brute Force optimization algorithm to find the optimal set of layer thicknesses meeting the requirements of design process. The Multiple Linear Regression (MLR) analysis was employed on the data set created by TMM

in order to formulate the mathematical relation between the structural parameters and sensor performance. The input parameters were made up of the thickness of MgO, Ag, and $BaTiO_3$ layers, while the response variables were sensitivity, R_{min}, FWHM, QF, DA, and FOM. In comparison to traditional optimization based on sweeping through values of the parameters resulting in maximum sensitivity of 401.40 deg/RIU, the proposed hybrid ML-assisted optimization resulted in increased sensitivity of 424.61 deg/RIU and improved sensing performance."

Reviewer#3, Concern #4: The manuscript claims strong agreement between TMM, FEM, and FDTD, but does not provide a clear quantitative comparison of results (e.g., resonance angle, reflectance, sensitivity) across the methods. Since this agreement is a key validation point, the paper should include explicit comparisons with error metrics. Simply stating "outstanding agreement" without numerical evidence or uncertainty analysis is insufficient.

Author Response: We would like to sincerely thank the reviewer for such an important suggestion. We completely agree that there should be clear presentation of the agreement between the various numerical techniques through quantitative data.

In the revised manuscript, we have enhanced our validation section by making a comparison of the reflectance profiles that are derived through the TMM and FEM techniques. It can be observed that both techniques give almost identical resonance properties, such as the resonance angle and resonance dip. In addition, to prove the electromagnetic characteristics of the designed sensor, FDTD simulations were carried out in order to investigate the electric field profile under resonance condition. It was found that the results obtained by the help of FDTD analysis are highly compatible with FEM simulations, where there is about 2.5×10^5 V/m for the electric field enhancement factor and 199 nm for the surface plasmon penetration depth by using two techniques.

For more elaboration on the corresponding results, the figures related to the results are presented to the modified manuscript. It is noteworthy that TMM analysis was used to determine the reflectance response whereas FEM and FDTD analysis have been used for the electromagnetic fields.

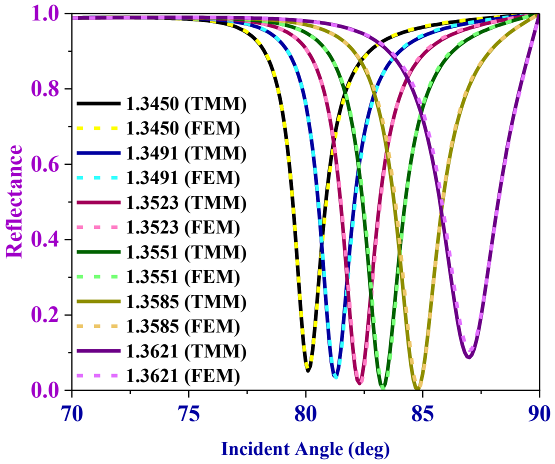

Fig. Numerical validation of the proposed SPR sensor: comparison of TMM and FEM-based reflectance curves.

Revised text (Page 18, Line 503-511, Fig. 13): "The validity of the proposed SPR sensor model was established by conducting a comparative analysis of the simulated reflectance spectrum and resonance angle calculated by both TMM (MATLAB) and FEM (COMSOL Multiphysics 6.2). Both models yielded very similar resonance curves with almost perfect matching in the resonance angle and minimum reflectance. The maximum difference in the calculated resonance angle by the two methods was shown to be less than 0.1°, ensuring the consistency and reliability of the used numerical models in terms of optical behavior predictions. In addition, the distribution of the electric field generated by the TMM model was verified by the FEM approach. Both models had a maximum electric field intensity of approximately 2.5×10^5 V/m and a plasmon penetration depth of approximately 200 nm."

Reviewer#3, Concern #5: The manuscript reports very fine mesh/grid settings and strict tolerances, but does not provide sufficient evidence of numerical convergence or stability. Key elements like mesh/grid convergence studies, time-window validation (for FDTD), and sensitivity to simulation parameters are missing. Without uncertainty analysis, it is unclear whether the reported performance improvements are numerically robust, especially since the gains over prior work are relatively modest.

Author Response: We are truly thankful to the reviewer for giving us such a constructive comment. The numerical convergence and convergence of the simulations are very important for making sure that the obtained performance of the sensors is reliable. As a result, the numerical convergence and simulation settings for both FEM and FDTD simulations have been added to **Section 2.1 (Modeling Framework and Simulation Strategy)**.

As far as FEM simulations are concerned, there was a very fine physics-controlled mesh used with mapped meshing for ultrathin multilayer films and tetrahedral meshing for the sensing region with the minimal mesh size of 0.2 nm. In order to achieve numerical convergence, the mesh was refined until variation in the obtained results was less than 0.1%. All simulations were carried out using PARDISO solver with relative tolerance of 1×10^{-6}. Likewise, for the FDTD simulations, grid sizes as small as 0.1 nm were employed to correctly capture the ultra-thin multi-layer structure and the associated evanescent electromagnetic fields. Moreover, a stringent convergence requirement of 1×10^{-6} was applied in order to guarantee the numerical stability and accuracy of the solutions. In addition, the results from the FDTD simulations were in good agreement with those from the TMM and FEM simulations in regards to the resonance properties and electromagnetic fields, which provides another support for the proposed model.

Also, the angle of resonance was computed based on an accurate angular sampling process to avoid any possible numerical error due to inaccurate angular discretization. Besides the above-mentioned analysis, in order to further prove the robustness of the proposed sensor, the performance under ±10% manufacturing tolerances was considered as well.

All the above information has been added in **Section 2.1(Modeling Framework and Simulation Strategy)** of the revised manuscript.

Reviewer#3, Concern #6: The manuscript models sensing as a simple refractive-index shift, assuming milk fat concentration directly maps to RI. However, milk is a complex, scattering colloidal medium, and its optical response depends on multiple factors beyond RI. Because these complexities are not considered, the study effectively validates only a generic RI sensor, not a realistic milk-fat detection system. The claim should be limited to RI sensing unless a more accurate milk model is included.

Author Response: In this regard, we are thankful to the reviewer for raising such an insightful point. We do acknowledge that milk is indeed a highly complicated colloidal solution where apart from the refractive index, its optical response is affected by scattering, particle size, proteins, temperature, etc. Thus, a comprehensive experimental model of milk needs to take into account all these factors as well.

However, in the current work, the main aim is to develop and optimize a sensing platform using simulation. The reason for modeling milk with the help of their effective refractive indices is because the refractive indices used in the current research are taken from experimentally reported values that can be found in the existing literature [4]. It should be kept in mind that the refractive index values are not arbitrary but rather experimental data relating milk composition and optical properties. In this regard, the current study is restricted by the fact that at present, our lab does not have the experimental setup required for making the proposed sensor.

To be more precise, the manuscript has been modified to show that the developed sensor works as a refractive index-based sensing platform due to the use of milk refractive index values as reported experimentally, and not as an experimentally proven milk-fat detection system. In addition, the section on future work has been extended. For specific and efficient milk-fat detection, the developed SPR platform could be functionalized with the biorecognition layer such as lipase, PAA-Chitosan-Lipase, and PMMA-Lipase. Lipase specifically binds with triglycerides which are the component of milk-fat, and through the process of enzyme hydrolysis breaks down triglycerides into fatty acids and glycerol [2], [3]. This interaction between biomolecules alters the local dielectric constant at the interface of sensor surface leading to shift in SPR resonance wavelength due to modification in the effective refractive index [7]. In addition, polymer supported immobilization of lipase such as PAA-Chitosan-Lipase and PMMA-Lipase enhances the stability of enzymes along with selective binding.

[2] M. Chakraborty and K. Biswas, "Hardware Platform to Detect Fat Percent in Milk Using a Lipase Immobilized PMMA-Coated Sensor," IEEE Trans. Instrum. Meas., vol. 68, no. 11, pp. 4526–4534, 2019, doi: 10.1109/TIM.2018.2890320.

[3] S. Zhou, X. Li, J. Zhang, H. Yuan, X. Hong, and Y. Chen, "Dual-fiber optic bioprobe system for triglyceride detection using surface plasmon resonance sensing and lipase-immobilized magnetic bead hydrolysis," Biosens. Bioelectron., vol. 196, p. 113723, Jan. 2022, doi: 10.1016/J.BIOS.2021.113723.

[4] A. J. Jääskeläinen, K. E. Peiponen, and J. A. Räty, "On reflectometric measurement of a refractive index of milk," J. Dairy Sci., vol. 84, no. 1, pp. 38–43, 2001, doi: 10.3168/jds.S0022-0302(01)74449-9.

[7] J. Homola, "Surface Plasmon Resonance Sensors for Detection of Chemical and Biological Species," Chem. Rev., vol. 108, no. 2, pp. 462–493, Feb. 2008, doi: 10.1021/cr068107d.

Revised text (Page 3, Line 100-114): "In future experiments, the sensitivity of the detection system may be improved by functionalizing the biosensor surface using biorecognition layers like lipase, PAA-Chitosan-Lipase, or PMMA-Lipase. Lipase enzyme acts on the triglycerides in milk fat and cleaves them into glycerol and

fatty acids. In addition to generating the biochemical reaction, there is a corresponding change in the local refractive index, which causes a shift in SPR resonance, thus enabling selective detection of milk fat [33]. Immobilization of enzymes using polymer assistance, such as in PAA-Chitosan or PMMA matrices, enhances the enzyme immobilization, reusability, and selectivity of biosensor systems [34], [35]. The recent SPR studies conducted with regard to the sensing of the milk fat quality have shown some promising results when using different combinations of materials and structural arrangements. Nevertheless, the study presented in [36] was mostly concerned with the biosensing performance without looking into structural optimization in detail. At the same time, the study in [37] focused on the development of increased sensitivity due to the use of material engineering. The same can be noted about the works presented in [38], [39]. However, no mathematical model for the fast sensor design was built in the course of these experiments. In its turn, this work uses the approach of multilayer structure optimization together with the predictive MLR model for fast performance estimation.

Reviewer#3, Concern #7: The manuscript refers to the device as a "biosensor," but the analysis is based only on bulk refractive-index sensing. It does not include key elements of true biosensing, such as surface functionalization, binding kinetics, or selective biorecognition mechanisms. As a result, the optical response is imposed through RI changes rather than a physical interfacial process, making the design more accurately a theoretical RI sensor rather than a demonstrated milk-fat biosensor.

Author Response: We are grateful to the reviewer for providing such insightful feedback. We agree with the reviewer that a real biosensor requires integration of a biorecognition layer, which should be selective enough, since sensing occurs due to biomolecular recognition processes taking place at the sensor surface. In the current paper, the proposed sensor was analyzed by computer simulations using experimentally measured refractive indices for various concentrations of fat in milk. Hence, the current research deals more with the sensing abilities of the proposed SPR platform, which is used as a refractive index sensor.

In order to eliminate possible misunderstandings, the manuscript has been corrected, specifying the scope of the current research, and stating that the achieved results depend on the variation of bulk refractive indices provided by experimental papers. Moreover, the section concerning future work has been elaborated by describing how the SPR sensor proposed in this study can become a biosensor due to the introduction of selective biorecognition films. For instance, lipase, PAA-Chitosan-Lipase, or PMMA-Lipase recognition films can specifically recognize and catalyze the hydrolysis of triglycerides that constitute milk fat [2], [3]. Such specific biochemical processes taking place at the interface influence the local dielectric properties near the surface, producing an SPR signal shift. The use of polymer-based support for enzyme immobilization enhances enzyme stability, immobilization efficacy, and selectivity of the biosensor, thus providing for the practical realization of milk-fat biosensing. Such clarifications were made to better formulate the boundaries of the current study and indicate a feasible way to future experiments.

[2] M. Chakraborty and K. Biswas, "Hardware Platform to Detect Fat Percent in Milk Using a Lipase Immobilized PMMA-Coated Sensor," IEEE Trans. Instrum. Meas., vol. 68, no. 11, pp. 4526–4534, 2019, doi: 10.1109/TIM.2018.2890320.

[3] S. Zhou, X. Li, J. Zhang, H. Yuan, X. Hong, and Y. Chen, "Dual-fiber optic bioprobe system for triglyceride detection using surface plasmon resonance sensing and lipase-immobilized magnetic bead hydrolysis," Biosens. Bioelectron., vol. 196, p. 113723, Jan. 2022, doi: 10.1016/J.BIOS.2021.113723.

Reviewer#3, Concern #8: The manuscript compares its sensor with prior SPR studies that are not specific to milk. [ref.51], [ref.52]. While general RI-based comparisons are acceptable for optical benchmarking, they do not fully support claims of superiority for milk fat sensing. The paper should clearly separate generic sensor comparisons from application-specific comparisons (e.g., milk or food matrices). Without this distinction, the practical relevance of the comparison is overstated.

Author Response: Thank you for this suggestion. We concur with the suggestion that there should be a distinction between the general SPR sensor benchmarking and the comparison specific to the applications.

The purpose of the comparison carried out in our manuscript was to compare the performance of the designed SPR sensor to those already in existence in the area of SPR sensors used for RI measurements in terms of sensitivity and other sensor parameters. This was done because there is currently a shortage of literature regarding milk fat detection using SPR techniques. As per the suggestion of the reviewer, we have made changes in our manuscript by including the target application for each of the reported SPR sensors in the comparison table. In addition, this change makes it clear that generic refractive index sensing and application-specific sensing are different aspects, and readers can judge the proposed sensor in its relevant context. In addition, it is clarified in the discussion that the purpose of comparison of the proposed sensor with generic SPR sensors is only to judge its performance in optical sensing, whereas comparison with application-specific sensors is to find out its utility for milk sensing.

Reviewer#3, Concern #9: The work is entirely simulation-based, with no experimental fabrication, measurements, or validation against standard milk-analysis methods. While simulation studies can be valid, this one lack sufficient physical rigor and clear model limitations. Combined with simplified analyte assumptions and unclear optimization, the results are not experimentally supported. The study should therefore be presented as a theoretical design rather than a validated milk-quality sensing solution.

Author Response: In any case, we want to express our sincere gratitude to the reviewer for making such a significant and relevant comment. It is necessary to admit that the current research paper is based on a computer simulation and does not include experimental fabrication, characterization, and validation by real milk samples. The main goal of this study was to create a theoretical model for the design, analysis, and optimization of a high-performing SPR sensor before its experimental realization.

The current sensor was created by applying well-known numerical techniques, such as Transfer Matrix Method (TMM), Finite Element Method (FEM), and Finite-Difference Time-Domain (FDTD). The refractive index of the milk samples was not assumed arbitrarily but taken from previously performed experiments described in scientific literature. That is why the simulation results are obtained on the basis of experimental optical parameters and structural optimization of the sensor only. Currently, our research laboratory lacks the experimental equipment needed to fabricate nanodevices, perform the optical and biochemical characterization of SPR devices. Thus, the scope of the current paper is confined to numerical design, optimization, and verification. However, we fully understand that in order to implement the proposed device it will be necessary to carry out experiments and calibrate the sensor by using actual milk

samples. In order to fulfill the request of the reviewer, we have modified the manuscript in order to clarify that the device proposed in the paper must be considered only as a theoretical platform for the refractive index sensing of milk rather than an experimentally validated device for milk quality analysis.

Moreover, we have provided the additional information about the future work which includes: fabrication of the optimized multilayer structures; surface functionalization of the devices with selective biorecognition layers, calibration with actual milk samples of different fat content and comparison with standard methods of milk analysis. We believe these modifications clarify the scope and limitations of our work.

Reviewer#3, Concern #10: The term "major breakthrough" is too strong for a simulation-only study without experimental validation

Author Response: Thank you very much for raising this valid point about our manuscript. We totally agree with the fact that the phrase "major breakthrough" may be inappropriate for the current study, which has been carried out solely on the basis of simulation without any kind of experimental validation. Thus, we have modified our manuscript accordingly and referred to our contribution as being a "promising numerical SPR sensing technique."

Revised text (Page 1, Line 23-24): "This study has made a promising numerical SPR sensing technique in the development of conventional SPR biosensors and has the potential to be used in the food industry for the monitoring of milk quality and adulteration.

Reviewer#3, Concern #11: The manuscript should clearly distinguish "high sensitivity" from "best practical sensor," because high angular sensitivity alone does not guarantee superior detectability.

Author Response: We sincerely thank the reviewer for this important comment. We agree that high angular sensitivity alone does not guarantee the best practical sensor. In the revised manuscript, we have clarified that the final design was not selected only based on maximum sensitivity. Other practical sensing parameters, such as minimum reflectance, FWHM, quality factor, detection accuracy, resonance sharpness, and fabrication feasibility, were also considered. For example, some Ag thickness values produced higher sensitivity, but they also showed higher minimum reflectance and poorer resonance behavior. Therefore, those configurations were not selected. The final design represents a balanced trade-off between high sensitivity and stable, detectable SPR response.

Reviewer#3, Concern #12: The wording around MgO as an "adhesive" and an optical enhancement layer should be clarified, since these are different physical roles.

Author Response: We appreciate the reviewer's valuable feedback and agree that clarification about the terminology in the discussion part of the paper is needed. MgO layer in the suggested biosensor is used mainly as the adhesion buffer layer for depositing Ag film on the prism

substrate. However, in addition to its main adhesion property, MgO layer affects the optical properties of the multilayer SPR sensor. Due to its dielectric nature, MgO layer alters the distribution of the electromagnetic field as well as the conditions of the surface plasmon wave coupling, leading to the shifting of the resonance angle and improved sensing capabilities. As seen from **Fig. 3**, adding the MgO layer leads to the variation in the resonance angle. Thus, while MgO layer is used as the adhesion buffer layer, it also acts as the optical enhancement layer due to the alteration of the resonance properties of the sensor.

Revised text (Page 9, Line 293-298): "The purpose of using MgO in the developed multilayer SPR sensor is mainly for improving the adhesion (buffering) function of the system that helps in achieving greater stability at the prism-Ag interface. Besides this, MgO also serves some other purposes in terms of optical behavior of the developed SPR sensor. Owing to its dielectric nature, MgO changes the condition of surface plasmon coupling and electromagnetic field distribution at the metal-dielectric interface, causing shifting in resonance angle and better sensing properties."

Reviewer#3, Concern #13: The BP layer thickness of 0.53 nm is effectively atomic-scale and needs a stronger fabrication and stability discussion.

Author Response: We sincerely appreciate the valuable suggestion made by the reviewer. It is true that we need to explain further on fabrication feasibility and stability of ultrathin BP layer.

The chosen thickness of BP (0.53 nm) is not an optimization parameter arbitrarily. In fact, this is the actual thickness of a single-layer black phosphorus (phosphorene) as reported in literature [9], [10]. This means the proposed sensor uses monolayer BP that has been extensively explored experimentally and successfully prepared using methods such as chemical vapor deposition (CVD), molecular beam epitaxy (MBE) and mechanical exfoliation [11], [12]. Here, monolayer BP was chosen owing to its excellent optical absorption, carrier mobility and light-matter interaction properties that enhance electromagnetic field confinement at sensing surface and consequently the performance of SPR sensing system. Furthermore, it should be noted that black phosphorus is prone to degradation in ambient atmosphere because of oxidation [13]. Nevertheless, it has been shown by some studies that its stability can be greatly enhanced if proper encapsulation or surface passivation is used by applying dielectric or polymeric protective layers, which renders BP applicable for photonic or plasmonic applications [14].

[9] H. Liu et al., "Phosphorene: An Unexplored 2D Semiconductor with a High Hole Mobility," ACS Nano, vol. 8, no. 4, pp. 4033–4041, Apr. 2014, doi: 10.1021/nn501226z.

[10] L. Li et al., "Black phosphorus field-effect transistors," Nat. Nanotechnol., vol. 9, no. 5, pp. 372–377, 2014, doi: 10.1038/nnano.2014.35.

[11] J. B. Smith, D. Hagaman, and H.-F. Ji, "Growth of 2D black phosphorus film from chemical vapor deposition," Nanotechnology, vol. 27, no. 21, p. 215602, Apr. 2016, doi: 10.1088/0957-4484/27/21/215602.

[12] X. Zhang and W. Zhang, "Synthesis of black phosphorus and its applications," Materials Today Physics, vol. 43, p. 101396, Apr. 2024, doi: 10.1016/J.MTPHYS.2024.101396.

[13] A. Favron et al., "Photooxidation and quantum confinement effects in exfoliated black phosphorus," Nat. Mater., vol. 14, no. 8, pp. 826–832, 2015, doi: 10.1038/nmat4299.

[14] J. Qiao, X. Kong, Z. X. Hu, F. Yang, and W. Ji, "High-mobility transport anisotropy and linear dichroism in few-layer black phosphorus," Nat. Commun., vol. 5, Jul. 2014, doi: 10.1038/ncomms5475.

Revised text (Page 4, Line 181-183): "It must be emphasized that the selected BP thickness value (0.53 nm) equals the experimental value of the thickness of a monolayer of black phosphorus It is a material characteristic; thus, the optimal thickness cannot be obtained [48], [49]."

Reviewer#3, Concern #14: The data-availability statement appears weak for a numerical paper; scripts, parameter files, and simulation settings should be shared if possible.

Author Response: Thank you very much for this useful recommendation, we appreciate the significance of making data available for the sake of transparency and reproducibility.

All the information about simulation settings, properties of the material, and methodological aspects needed to reproduce the presented results is described in detail in the revised version of our manuscript. Nevertheless, at the present moment, the complete set of optimization codes and parameters is not available for public use, as they include some proprietary research material.

For the sake of making scientific findings reproducible but also preserving proprietary research material, the Data Availability Statement has been revised in a way that makes it clear that the simulation data, parameter files, and computational materials related to this research can be provided by the corresponding author on request.

Reviewer#3, Concern #15: Perform true multidimensional optimization (or a well-documented surrogate/evolutionary method) instead of sequential sweeps.

Author Response: We are grateful to the reviewer for making such a constructive suggestion. Indeed, any optimization framework should consider the interaction between multiple design parameters in a design process instead of a simple sequential sweep through them. To address this issue, we have modified the manuscript to emphasize that the sweeps through individual parameters (thicknesses) of interest were conducted just for the initial screening and generation of the training data set using the TMM method. Therefore, such a sweep is not a part of the actual optimization algorithm.

This training data set is utilized next for the training of the Multiple Linear Regression (MLR) model representing a surrogate predictive model for predicting the relation between the thicknesses of the layers (MgO, Ag, $BaTiO_3$) and the corresponding sensing performance. This model is used further together with a Brute Force optimization algorithm to find the optimal parameters by conducting multidimensional search considering the interaction between multiple design parameters. In order to make the optimization procedure clear and unambiguous, we have revised and explained it clearly in the manuscript. It should also be noted that more sophisticated optimization procedures, like evolutionary algorithms or multi-objective Pareto-based optimization, are possible directions for future research.

Reviewer#3, Concern #16: Include convergence studies for mesh, grid, and angular steps.

Author Response: Thank you very much to the reviewer for such an important comment. It should be pointed out that the relevant information about the convergence is already presented in the manuscript. Specifically, in Section 2.1, the FEM calculation was done with an ultrafine mesh with a relative tolerance of 1×10^{-6}, and the convergence was estimated via mesh refinement, with variation below 0.1% between subsequent results. In the case of FDTD calculations, the grid size was set at 0.1 nm or below with a convergence threshold of 1×10^{-6}. Angular interrogation was conducted by means of an ultrafine angular sweep in the considered incident angle range for accurate determination of the resonance angle. This ensures independence of resonance and sensitivity from inadequate mesh/grid/angle resolution.

All the above information has been added in **Section 2.1 (Modeling Framework and Simulation Strategy)** of the revised manuscript.

Reviewer#3, Concern #17: Add fabrication-tolerance and uncertainty analysis to test robustness.

Author Response: We would like to extend our sincere gratitude to the reviewer for his/her suggestion. We would like to state clearly that the analysis of fabrication tolerance and robustness has already been included in the manuscript. Particularly, the effect of ±10% change in thickness of the multilayers has been investigated in terms of robustness, and the results obtained are tabulated in **Table 5**.

Table 5. Influence of ±10% thickness error on the performance metrics of the proposed sensor for RI of 1.3621

Performance parameter	Error possibility of all layers for +10%	Error possibility of all layers for -10%
Sensitivity (deg/RIU)	443.04	333.56
R_{min}	0.752	0.032
FWHM	3.343	2.625
DA (deg^{-1})	0.299	0.381
QF (RIU^{-1})	132.52	127.07
FOM	32.86	122.93

Reviewer#3, Concern #18: Use a more realistic model for milk (e.g., effective-medium approach) or limit claims to generic RI sensing.

Author Response: We would like to thank the reviewer for this suggestion. Indeed, a more elaborate optical model, such as the effective medium model considering the heterogeneity of milk, may lead to a more accurate representation of the sample of interest.

However, in the current study, our main aim was the development of an optimized refractive-index sensor through the numerical simulation of the SPR. For this purpose, the experimental data on the effective refractive index of milk was used to represent the samples instead of an elaborate optical model. Such approach is quite common in numerical simulations of SPR sensors. Following the suggestion of the reviewer, we modified our manuscript and mentioned that the proposed device should be considered as the refractive-index based sensing platform using the experimental data on the milk refractive index rather than the milk-fat sensor.

Moreover, we further develop the section on the potential avenues for future research by highlighting the fact that the suggested SPR platform may be converted into a selective and operational biosensor system upon introducing a biomarker-selective biorecognition layer. Thus, one can immobilize lipase, PAA-Chitosan-Lipase, or PMMA-Lipase sensing layers, which will enable the selective recognition and catalytic conversion of triglycerides, the main components of milk fat. The changes in the local dielectric constant due to such molecular interactions result in the SPR resonance shift, which allows us to realize selective detection of milk fat. Moreover, future research may also include the usage of effective medium approximations, combined with experimental validation, performed with real milk samples, taking into account the complicated nature of the milk optics. These improvements help to clarify the limitations and possibilities of the current study.

Revised text (Page 19, Line 514-531):

"3.8 Simulation Scope, Limitations, and Future Directions

This paper considers the modeling of the design, analysis, and optimization of the SPR sensing device proposed for the detection of milk fat by using the experimental refractive indices of different concentrations of milk fat. The purpose of this research is the investigation of the performance of the designed multilayer SPR structure with respect to the refractive index sensing and the optimal setting of its parameters for getting high sensitivity and narrow resonant spectrum. The validation of the numerical simulation model was performed by means of cross-checking of the results obtained by means of TMM and FEM methods. Despite all these promising results, some restrictions must be mentioned. The current research is totally theoretical and involves simulations only without actual manufacturing and characterization of the designed sensor. Additionally, the sensing principle is based on the variations in refractive index and does not take into account surface functionalization, biomolecular interactions or molecular selectivity. Thus, the obtained results should be treated as the refractive index sensing ability of the suggested SPR-based platform and not as the actual milk fat biosensor. Indeed, the real milk is quite a complicated biological medium which consists of fats, proteins, carbohydrates, minerals, water and other components; hence, some additional studies will be needed to test the sensor performance in the conditions where different physicochemical parameters are changing at the same time. The future works will involve the manufacturing and experimental testing of the suggested sensor using real milk samples. In addition, it is possible to use some selective bio-recognition layers, like lipase, PAA-Chitosan-Lipase and PMMA-Lipase coatings for the triglyceride recognition among others."

Reviewer#3, Concern #19: Analyze surface adsorption effects to support the "biosensor" claim.

Author Response: Thank you very much to the reviewer for making such a useful suggestion. It is our understanding that surface adsorption and specific molecular interactions are important properties of a real-life SPR biosensor.

The main goal of the present work was focused on development and optimization of the simulation of the SPR refractive-index detection platform. Thus, the sensing process in the current study was simulated based on experimentally obtained data about refractive index for various concentrations of milk fat without including surface adsorption or any binding kinetics. In order to make clearer the boundaries of the current research, we decided to modify the manuscript and explicitly state that the current research is an example of the performance of the proposed SPR refractive-index sensing platform rather than a developed biosensor. We also modified the section on further work where the described platform could be converted into the biosensor by adding specific recognition layers. Indeed, lipase, PAA-Chitosan-Lipase, or PMMA-Lipase functional layers can be used on the sensor surface to specifically react with

triglycerides, the main biomarker for the presence of milk fat. The specific adsorption and enzymatic reaction of the targeted molecules on the sensor surface will result in changes in the dielectric properties around that region and consequently induce a change in the resonance angle through SPR. This combination is considered a crucial future area of investigation.

These changes have been made to the paper to separate the current numerical investigation from future experimental realization of the biosensor.

Revised text (Page 3, Line 100-114): "In future experiments, the sensitivity of the detection system may be improved by functionalizing the biosensor surface using biorecognition layers like lipase, PAA-Chitosan-Lipase, or PMMA-Lipase. Lipase enzyme acts on the triglycerides in milk fat and cleaves them into glycerol and fatty acids. In addition to generating the biochemical reaction, there is a corresponding change in the local refractive index, which causes a shift in SPR resonance, thus enabling selective detection of milk fat [33]. Immobilization of enzymes using polymer assistance, such as in PAA-Chitosan or PMMA matrices, enhances the enzyme immobilization, reusability, and selectivity of biosensor systems [34], [35]. The recent SPR studies conducted with regard to the sensing of the milk fat quality have shown some promising results when using different combinations of materials and structural arrangements. Nevertheless, the study presented in [36] was mostly concerned with the biosensing performance without looking into structural optimization in detail. At the same time, the study in [37] focused on the development of increased sensitivity due to the use of material engineering. The same can be noted about the works presented in [38], [39]. However, no mathematical model for the fast sensor design was built in the course of these experiments. In its turn, this work uses the approach of multilayer structure optimization together with the predictive MLR model for fast performance estimation."

Response to Reviewer #4

Reviewer#4, Concern #1: It's worth taking note of the issue of selecting the right prism material for measuring milk's fat content, which was ultimately determined to be SiO_2. Also, a clear explanation of the mechanism of operation of the SPR sensors would be a good read.

Author Response: Thank you very much to the reviewer for giving this important recommendation. We agree with the reviewer that choosing the right prism material and also providing a clear understanding about the sensing principle is critical for increasing the scientific validity of the manuscript. On choosing the prism material, the choice of SiO_2 was not a mere coincidence. In our study, several materials were tested for use in prisms, such as SiO_2, BaF_2, CsF, BK7, and BAK1, in similar simulation conditions. Their sensing performance in relation to sensitivity, minimum reflectance (Rmin), Q-factor, and accuracy of detection at different refractive indices, which were related to different concentrations of milk fat, was compared. Out of the materials chosen for testing, SiO_2 showed a more consistent and better performance compared to others, as it offered higher sensitivity and also better resonance properties (shown in **Fig 4** in the manuscript). Thus, based on this comparison, SiO_2 was chosen as the best prism material.

Addressing the reviewer's second concern, we have elaborated further on the operating mechanism of our proposed SPR sensor in the revised manuscript. Our proposed sensor utilizes the angular interrogation mode in the Kretschmann geometry. The p-polarized monochromatic light is incident on the metal layer via the prism at different incident angles. When the incident light achieves a particular resonance angle, its in-plane component becomes equal to the propagation constant of the surface plasmon waves at the interface of the metal and dielectric medium, thereby leading to the energy transfer between the two and causing the formation of a dip in the reflected light intensity [15], [16]. If there is any change in the composition of the milk sample, especially the fat content, there is an effect on the effective refractive index of the analyte solution [17]. This causes a deviation in the surface plasmon resonance matching condition and subsequently leads to the change in the resonance angle [7].

The sensing mechanism can therefore be summarized as:
Variation in milk fat concentration → Change in analyte refractive index → Modification of SPR coupling condition → Resonance angle shift → Quantification of milk fat concentration.

[7] J. Homola, "Surface Plasmon Resonance Sensors for Detection of Chemical and Biological Species," Chem. Rev., vol. 108, no. 2, pp. 462–493, Feb. 2008, doi: 10.1021/cr068107d.

[15] A. Uniyal, G. Srivastava, A. Pal, S. Taya, and A. Muduli, "Recent Advances in Optical Biosensors for Sensing Applications: a Review," Plasmonics, vol. 18, no. 2, pp. 735–750, Apr. 2023, doi: 10.1007/S11468-023-01803-2.

[16] V. Yesudasu, H. S. Pradhan, and R. J. Pandya, "Recent progress in surface plasmon resonance based sensors: A comprehensive review," Mar. 01, 2021, Elsevier Ltd. doi: 10.1016/j.heliyon.2021.e06321.

[17] A. Poonia, A. Jha, R. Sharma, H. B. Singh, A. K. Rai, and N. Sharma, "Detection of adulteration in milk: A review," Int. J. Dairy Technol., vol. 70, no. 1, pp. 23–42, Feb. 2017, doi: https://doi.org/10.1111/1471-0307.12274.

Reviewer#4, Concern #2: On the other hand, I am concerned about the assumption in the simulations that the thickness of some layer's changes by 0.1 nm, which corresponds to the size of an atom or smaller, and therefore is probably not wholly justified.

Author Response: Thank you very much to the reviewer for this comment. We fully understand that a thickness variation of 0.1 nm is far from being a practical fabrication precision level. We would like to apologize if there were any ambiguities related to the presentation of our research results.

In this paper, a step of 0.1 nm has been used only as the resolution of the numeric search for optimization, but not as the required fabrication accuracy. The resolution of the scan has been chosen to find precisely the thickness for the global optimum in the simulated parameter space without missing any sharp resonances that are typical for the SPR sensors. In the case of the last designed sensor, there is no assumption of an unrealistically accurate thickness value for $BaTiO_3$. Even though the numerical optimization suggested that the optimum $BaTiO_3$ thickness is approximately 3.2 nm, the design of the practical sensor assumes 3 nm of $BaTiO_3$ material, which is a practical nominal thickness that could be achieved by the state-of-the-art methods such as ALD, Magnetron Sputtering, and Pulsed Laser Deposition (PLD) [18], [19]. The same way, the optimized thicknesses of MgO and Ag materials are integer nanometer values as well, highlighting the practicality of the design. In terms of fabrication practicality, we already have provided fabrication tolerance (±10%) analysis in the revised version of the manuscript. It shows that the proposed sensor is robust enough to perform sensing tasks with realistic thickness deviations; therefore, the performance of the sensor does not depend on a sub-nanometer accuracy of fabrication.

[18] S. M. George, "Atomic Layer Deposition: An Overview," Chem. Rev., vol. 110, no. 1, pp. 111–131, Jan. 2010, doi: 10.1021/cr900056b.

[19] S. R. Lee et al., "First Observation of Ferroelectricity in ~1 nm Ultrathin Semiconducting BaTiO3 Films," Nano Lett., vol. 19, no. 4, pp. 2243–2250, Apr. 2019, doi: 10.1021/acs.nanolett.8b04326.

Reviewer#4, Concern #3: It is also not entirely clear to me how the calculated sensitivity of the sensor translates into the sensitivity of measuring the fat content in milk.

Author Response: Thank you for your concern. We fully understand the importance of clarifying the correlation between SPR sensitivity and the detection of milk fat content. It is important to note that the suggested SPR sensor does not detect the concentration of milk fat, instead it detects changes in the effective refractive index (RI) of the milk sample. The changing concentration of milk fat changes the optical properties of the milk, which causes a change in the effective refractive index of the milk sample [7]. Accordingly, the sensing process can be summarized as follows: Variation in milk fat concentration → Change in effective refractive index → Modification of SPR resonance condition → Resonance angle shift.

The sensitivity is defined by the ratio of the variation of refractive index (Δn) and variation of resonance angle ($\Delta\theta_{res}$) with respect to each other. It is also defined by [20]:

$$S = \frac{\Delta\theta_{res}}{\Delta n} \text{(in deg/RIU)}$$

In the current study, the sensitivity of the designed sensor is provided in units of deg/RIU and represents the resonance angle shift with respect to the unit variation in refractive index. For instance, the highest sensitivity of 401.40 deg/RIU obtained at RI = 1.3621 shows that even slight changes in the value of refractive index due to any variations in milk fat concentration can result in detectable resonance angle shifts. It is important to emphasize that in order to convert the SPR signal from refractive index units into the absolute concentration of fat, an experimentally determined correlation between the refractive index and milk fat concentration needs to be used. In the case of the current work where the goal is the design and optimization of the SPR sensor by means of computer simulation, the experimentally reported values of refractive index for various fat concentrations have been used as the simulation input data.

[20] P. Maurya, A. Kushwaha, and R. Verma, "High Performance SPR Gas Sensor by Using Heterostructure of 2D Materials Graphene / Black Phosphorous / MoS 2," Sens. Imaging, pp. 1–20, 2024, doi: 10.1007/s11220-024-00518-w.

Reviewer#4, Concern #4: The authors could add a few review publications on the measurement of fat in milk and in general of its adulteration [1-3], and above all, refer to publications dealing with exactly the same problem, i.e. the measurement of fat content in milk using SPR sensors. I am referring to the publications listed in the list below [4-7].

Author Response: We sincerely appreciate this suggestion from the reviewer. The cited sources were added in the Introduction part of the study to further build on the literature review background and to place the current research in the appropriate context. In response, the recommended recent publications have been carefully reviewed and incorporated into the revised manuscript as Refs. [6], [7], [8], [36], [37], [38], and [39].

Reviewer#4, Concern #5: It's happened to me a few times that authors describe sensors in Kretschmann configuration and treat them as common knowledge, without citing their author [8]. It's crucial to give credit to the author and include him in the references.

Author Response: We are truly grateful to the reviewer for pointing out this pertinent. The reference has been included in the context where we have made a mention of the Kretschmann configuration. In response, the recommended recent publications have been carefully reviewed and incorporated into the revised manuscript as Refs. [23].

Response to Reviewer #5

Reviewer#5, Concern #1: How does the proposed hybrid ML optimization differ from conventional parameter sweeps? What scientific advancement beyond incremental sensitivity enhancement is achieved?

Author Response: We would like to express our gratitude to the reviewer for such a constructive remark. Indeed, the suggested ML-based hybrid optimization is different from the conventional parameter sweep in terms of the methods and goals. The conventional parameter sweep implies checking the performance of certain combinations of layer thicknesses and selecting the one that delivers the highest performance. Applying this method in our case, we obtained the sensitivity of 401.40 deg/RIU. In order to make the sensor more efficient, the dataset generated by the TMM was used to train an ML model that is able to learn the nonlinear dependencies between the structural parameters and the respective parameters of sensing. Then, the developed model was applied together with the Brute Force optimization algorithm to find optimal solutions in the design space. With the help of the described method, we have obtained the sensitivity of 424.61 deg/RIU, which is noticeably higher than that of the conventional parameter sweep. Moreover, improvements have been noticed in other performance parameters such as detection accuracy, quality factor, and figure of merit. Thus, the significance of this research goes beyond a mere enhancement in sensitivity. The novel method is a combination of the precision of the TMM with the predictability of machine learning to develop an optimization technique for SPR sensor design guided by physics. As opposed to the use of extensive parameter sweep optimization, the machine learning model is able to establish a connection between the layer thickness and performance of the sensor. Thus, the presented approach offers a viable framework for optimization that can be scaled up in the future to accommodate different SPR sensors with multiple layers.

Reviewer#5, Concern #2: The title emphasizes "Hybrid Machine Learning Driven Optimization," yet the ML methodology lacks sufficient technical detail such as: Type of ML model used, Input features and output targets, Training/testing dataset size, Data splitting strategy, Hyperparameters, Validation metrics (RMSE, MAE, R^2, etc.), Comparison with conventional optimization methods and Computational efficiency gains.

Author Response: We thank the reviewer very much for the constructive comment. According to the reviewer's advice, we have restructured the Machine Learning Methodology section to give a detailed explanation of our framework of hybrid optimization.

In the revised manuscript, it is clearly indicated that our framework combines the Transfer Matrix Method (TMM), Multiple Linear Regression (MLR), and Brute Force optimization approach. First, TMM was utilized to produce a simulation data set by adjusting the thicknesses of MgO, Ag, and $BaTiO_3$ in certain ranges and obtaining the corresponding sensing properties. There were 726 data points in this data set with the input features being the thicknesses of MgO, Ag, and $BaTiO_3$ and the output target variables being sensitivity, Rmin, FWHM, Q-factor, DA, and FOM. To investigate the prediction performance of the regression model, the data set was split into 80% training data and 20% testing data. To improve the technical description of the ML model, we have now included the developed Multiple Linear Regression

equations for all performance metrics together with their corresponding Mean Square Error (MSE) values in the revised manuscript. For example, the regression model for sensitivity is expressed as, $Sensitivity = 61.8687 + 0.4900(MgO) + 1.4649(Ag) + 60.0287(BaTiO_3)$, with an MSE of 3.4763. Similar regression models have also been presented for Rmin (MSE = 0.0004), FWHM (MSE = 0.0024), Q (MSE = 1.0857), DA (MSE ≈ 0.0000), and FOM (MSE = 1.2696).

Afterward, the trained regression model was combined with the Brute Force algorithm to explore the multidimensional design space and find the best layer thickness combination. The proposed hybrid optimization method showed higher sensitivity than the conventional parameter sweeps optimization method by reaching the highest sensitivity of 424.61 deg/RIU from 401.40 deg/RIU. At the same time, the sensing characteristics of the device were improved.

Revised text (Page 14, Line 385-404 and Page 15, Line 425-427): "For angular interrogation SPR sensors, an intensive resonance dip is critical for determining the resonance angle correctly. The smaller R_{min} implies better coupling of the light from the beam into surface plasmons and, thus, the higher plasmon excitation and better resonance contrast. Therefore, one could consider $R_{min} < 0.1$ as a practical criterion for achieving high enough resonance dip while optimizing the sensor design. The method ensures an optimal solution by avoiding local minima. In order to determine the relationship between structural parameters and performance of sensors, the Multiple Linear Regression (MLR) model was utilized. Simulation dataset was created based on Transfer Matrix Method (TMM) by changing thicknesses of MgO (0-20 nm), Ag (30-60 nm), and $BaTiO_3$ (1-5 nm) layers, while fixing the thickness of silicon layer to be 0.53 nm. Thus, 726 samples were obtained, where the input features were thicknesses of MgO, Ag, and $BaTiO_3$ layers, and output features were sensitivity, Rmin, FWHM, Q, DA, and FOM. Training data constituted 80% of all available dataset, while testing data were the remaining 20%. The performance of regression model for prediction was determined via Root Mean Square Error (RMSE), Mean Absolute Error (MAE) and coefficient of determination (R^2). Trained regression model was further incorporated into Brute Force optimization algorithm to find the optimal set of layer thicknesses meeting the requirements of design process. The Multiple Linear Regression (MLR) analysis was employed on the data set created by TMM in order to formulate the mathematical relation between the structural parameters and sensor performance. The input parameters were made up of the thickness of MgO, Ag, and $BaTiO_3$ layers, while the response variables were sensitivity, R_{min}, FWHM, QF, DA, and FOM. In comparison to traditional optimization based on sweeping through values of the parameters resulting in maximum sensitivity of 401.40 deg/RIU, the proposed hybrid ML-assisted optimization resulted in increased sensitivity of 424.61 deg/RIU and improved sensing performance."

"The obtained equations were capable of measuring the effect of each structural parameter on the sensor performance. The models that were developed showed very low mean square error (0.0000 ~ 3.4763), indicating their good predictive ability."

Reviewer#5, Concern #3: The manuscript assumes RI variation from 1.345 to 1.3621 corresponding to milk fat concentration. The authors should: provide experimental references correlating RI with fat percentage, clarify whether temperature dependence is considered, discuss compositional variability in real milk samples.

Author Response:

We sincerely acknowledge the insightful feedback of the reviewer. It is important to state that all the results shown in the paper are based on computer simulation and do not involve any experimental data acquisition process. The values of the refractive index (RI) from 1.345 to 1.3621 were used in accordance with previous publications where experimental correlations between RI and composition changes in milk, including its fat content, have been established

[4], [5]. Suitable literature references have been provided in the revised version of the manuscript. Additionally, it has been specified in the manuscript that variations of the RI due to the effect of temperature change were not taken into account in the present simulations since the aim was to assess the sensor intrinsic performance under controlled conditions. Moreover, it has been noted that the effect of temperature and other constituents, including proteins, lactose, and minerals in addition to fat, might also affect the RI in practical milk samples.

[4] A. J. Jääskeläinen, K. E. Peiponen, and J. A. Räty, "On reflectometric measurement of a refractive index of milk," J. Dairy Sci., vol. 84, no. 1, pp. 38–43, 2001, doi: 10.3168/jds.S0022-0302(01)74449-9.

[5] B. Karki, Y. Trabelsi, A. Uniyal, A. Pal, and R. Bharos Yadav, "Detection of fat concentration milk using TMDC-based surface plasmon resonance sensor," Modern Physics Letters B, vol. 38, no. 29, Oct. 2024, doi: 10.1142/S0217984924502531.

Revised text (Page 2, Line 92-98): "The detection principle behind the suggested SPR sensor is based on angular interrogation. The alterations in the milk's components composition, such as alterations in the fat component or in its adulteration, alter the effective refractive index of the medium, as shown by experimental studies [32]. Such alterations in the effective refractive index change the momentum matching required for exciting surface plasmons in the metal-analyte interface, thereby causing an alteration in the SPR resonance angle. Consequently, the suggested sensor indirectly senses any alterations in milk's components composition through the alteration in its refractive index."

Reviewer#5, Concern #4: Explain whether adulterants or proteins affect RI measurements.

Author Response: We appreciate the valuable input of the reviewer in this regard. Refractive index (RI) of milk depends upon its chemical composition in general. Milk proteins including whey protein and casein make up RI since they increase the number of dissolved solids. Similarly, any adulterant in the milk will change its chemical composition and thus, the RI of milk [21]. The addition of water in the milk will reduce RI due to dilution effect. At the same time, the presence of urea, melamine, or any other dissolved adulterant will increase RI depending upon their amounts. Thus, RI measurement can be affected by changes in the number of proteins or by adulteration of milk [17].

The significant point here is that the current research work concentrates on the use of the sensing technique based on refractive index and does not involve any selective bio-recognition layer. The selectivity of the new SPR technique in practical use can be increased by modifying the sensing surface using appropriate biomarkers or bio-recognition agents. For example, the coatings such as PAA-Chitosan-Lipase or PMMA can be used for selective binding of triglycerides without any interference from other components of milk such as proteins, lactose, and dissolved adulterants [1], [2]. Such a modification will lead to a conversion of the suggested SPR technique from the refractive index sensor to the milk fat biosensor.

[1] O. Kirk and M. W. Christensen, "Lipases from Candida antarctica: Unique Biocatalysts from a Unique Origin," Org. Process Res. Dev., vol. 6, no. 4, pp. 446–451, Jul. 2002, doi: 10.1021/op0200165.

[2] M. Chakraborty and K. Biswas, "Hardware Platform to Detect Fat Percent in Milk Using a Lipase Immobilized PMMA-Coated Sensor," IEEE Trans. Instrum. Meas., vol. 68, no. 11, pp. 4526–4534, 2019, doi: 10.1109/TIM.2018.2890320.

[17] A. Poonia, A. Jha, R. Sharma, H. B. Singh, A. K. Rai, and N. Sharma, “Detection of adulteration in milk: A review,” Int. J. Dairy Technol., vol. 70, no. 1, pp. 23–42, Feb. 2017, doi: https://doi.org/10.1111/1471-0307.12274.

[21] J. R. de Wolf, K. Ali, C. G. Legtenberg, W. Verveld, and N. Bosschaart, “The Refractive Index of Human Milk Serum: Natural Variations and Dependency on Macronutrient Concentrations,” Foods, vol. 13, no. 24, Dec. 2024, doi: 10.3390/foods13244124.

Reviewer#5, Concern #5: Fig. 11 captions appear inconsistent: “The RI of the corresponding fluoride concentration...” This is likely an editing error because the manuscript concerns milk fat detection, not fluoride concentration. All figure captions should be revised carefully.

Author Response: We sincerely thank the reviewer for identifying this editing error. We agree that the phrase “fluoride concentration” is incorrect and not relevant to the present study, which focuses on milk fat detection. The caption of Fig. 11 has been corrected and all figure captions have been carefully checked to avoid similar inconsistencies.

Reviewer#5, Concern #6: Several grammatical and stylistic issues are present throughout the manuscript. Professional English editing throughout the manuscript is highly recommended.

Author Response: We truly appreciate this valuable feedback from the reviewer. We fully acknowledge that language plays a key role in making the paper more understandable and readable. As a result, we have made some significant edits concerning grammar, sentence structure, vocabulary, and scientific writing. Many ambiguous sentences have been corrected for better readability and consistency.

Reviewer#5, Concern #7: Repetitive sentence structures are observed, such as: “This is because...” appears repeatedly in the Introduction.

Author Response: We sincerely thank the reviewer for this valuable observation. We have thoroughly checked the introduction and corrected all the repeating sentence constructions in the manuscript. In particular, repeated expressions such as "This is because..." have been rephrased or replaced with more appropriate alternatives to improve the readability, flow, and overall quality of the writing. These revisions have been incorporated into the revised manuscript.

Revised text (Page 2, Line 69-70): “The dip is caused by transfer of energy from photons to plasmons. It is due to high sensitivity of surface plasmon resonance (SPR) to refractive index changes [21].”

Reviewer#5, Concern #8: Some references appear duplicated: References [15] and [16] seem identical.

Author Response: We sincerely thank the reviewer for identifying this issue. We have carefully checked the reference list and confirmed that References [15] and [16] were duplicated. The duplicate reference has been removed, and the reference numbering and in-text citations have been updated accordingly throughout the manuscript.

Reviewer#5, Concern #9: Typographical issues in many instances such as: "Polinomial Fit" should be "Polynomial Fit."

Author Response: We sincerely thank the reviewer for pointing out this typographical issue. The typo "Polinomial Fit" has been corrected to "Polynomial Fit." In addition, the manuscript has been carefully checked to correct similar spelling, typographical, and formatting errors throughout the text, figures, and captions.

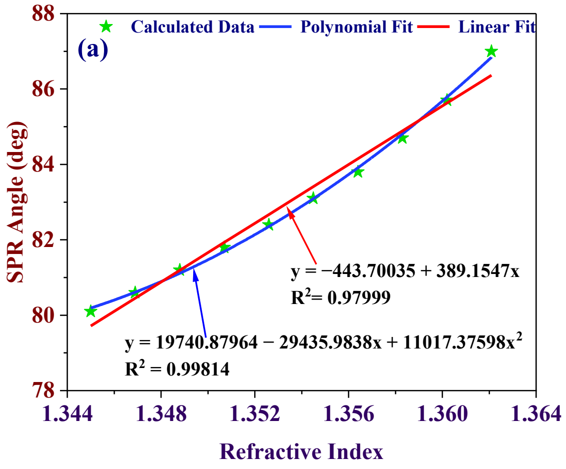

Fig. 11 (a) The RI of the corresponding ~~fluoride concentration~~ fat in milk is polynomial and linear correlated with the resonance dip angle.

Reviewer#5, Concern #10: The introduction discussion is poor and insufficiently structured. The literature review lacks critical analysis of recent advancements in multilayer SPR biosensors, particularly those involving hybrid dielectric materials, 2D materials, and machine learning-assisted optimization techniques. The authors are strongly advised to enrich the Introduction section using:doi.org/10.1007/s11468-025-02961-1, 10.1016/j.microc.2026.117902, doi: 10.1109/TNB.2026.3664930, doi.org/10.1007/s11468-025-02823-w.

Author Response: We would like to thank the reviewer for this useful suggestion. In the Introduction part, these new references have been added as well as the discussion on multilayer surface plasmon resonance (SPR) biosensors, hybrid dielectrics, two-dimensional materials, and machine learning (ML)-based optimization techniques. These help improve the background information and explain the gap in this research. In response, the recommended recent publications have been carefully reviewed and incorporated into the revised manuscript as Refs. [15], [28], [53], and [66].
