# Supplementary material for: Hybrid machine learning driven optimization of multilayer SPR sensor for high sensitivity milk fat detection
Source: PLoS One. 2026 Sep 2;21(9):e0355996. doi: 10.1371/journal.pone.0355996 (PMC13537604; doi:10.1371/journal.pone.0355996)
Supplement: Peer review history

# Peer review: Decision Letter 0

**DOI:** 10.1371/journal.pone.0355996.r001

**Authors:** Zaky Zaky

21 May 2026

Dear Dr. Utshob,

Thank you for submitting your manuscript to PLOS ONE. After careful consideration, we feel that it has merit but does not fully meet PLOS ONE’s publication criteria as it currently stands. Therefore, we invite you to submit a revised version of the manuscript that addresses the points raised during the review process.

==============================

**ACADEMIC EDITOR:**

In light of the reviewers’ comments, the manuscript is invited for a major revision. The authors are requested to review the comments carefully and submit a revised manuscript with appropriate responses.

==============================

Please submit your revised manuscript by Jul 05 2026 11:59PM. If you will need more time than this to complete your revisions, please reply to this message or contact the journal office at plosone@plos.org. When you're ready to submit your revision, log on to https://www.editorialmanager.com/pone/ and select the 'Submissions Needing Revision' folder to locate your manuscript file.

Please include the following items when submitting your revised manuscript:

- A letter that responds to each point raised by the academic editor and reviewer(s). You should upload this letter as a separate file labeled 'Response to Reviewers'.
- A marked-up copy of your manuscript that highlights changes made to the original version. You should upload this as a separate file labeled 'Revised Manuscript with Track Changes'.
- An unmarked version of your revised paper without tracked changes. You should upload this as a separate file labeled 'Manuscript'.

If you would like to make changes to your financial disclosure, please include your updated statement in your cover letter. Guidelines for resubmitting your figure files are available below the reviewer comments at the end of this letter.

If applicable, we recommend that you deposit your laboratory protocols in protocols.io to enhance the reproducibility of your results. Protocols.io assigns your protocol its own identifier (DOI) so that it can be cited independently in the future. For instructions see: https://journals.plos.org/plosone/s/submission-guidelines#loc-laboratory-protocols. Additionally, PLOS ONE offers an option for publishing peer-reviewed Lab Protocol articles, which describe protocols hosted on protocols.io. Read more information on sharing protocols at https://plos.org/protocols?utm_medium=editorial-email&utm_source=authorletters&utm_campaign=protocols.

As the corresponding author, your ORCID iD is verified in the submission system and will appear in the published article. PLOS supports the use of ORCID, and we encourage all coauthors to register for an ORCID iD and use it as well. Please encourage your coauthors to verify their ORCID iD within the submission system before final acceptance, as unverified ORCID iDs will not appear in the published article. *Only*  the individual author can complete the verification step; PLOS staff *cannot*  verify ORCID iDs on behalf of authors.

We look forward to receiving your revised manuscript.

Kind regards,

Zaky A. Zaky, Ph.D.

Academic Editor

PLOS One

Journal Requirements:

When submitting your revision, we need you to address these additional requirements.

1. Please ensure that your manuscript meets PLOS ONE's style requirements, including those for file naming. The PLOS ONE style templates can be found at

https://journals.plos.org/plosone/s/file?id=wjVg/PLOSOne_formatting_sample_main_body.pdf and

https://journals.plos.org/plosone/s/file?id=ba62/PLOSOne_formatting_sample_title_authors_affiliations.pdf

2. Please note that PLOS One has specific guidelines on code sharing for submissions in which author-generated code underpins the findings in the manuscript. In these cases, we expect all author-generated code to be made available without restrictions upon publication of the work. Please review our guidelines at https://journals.plos.org/plosone/s/materials-and-software-sharing#loc-sharing-code and ensure that your code is shared in a way that follows best practice and facilitates reproducibility and reuse.

3. Please provide a complete Data Availability Statement in the submission form, ensuring you include all necessary access information or a reason for why you are unable to make your data freely accessible. If your research concerns only data provided within your submission, please write "All data are in the manuscript and/or supporting information files" as your Data Availability Statement.

4. If the reviewer comments include a recommendation to cite specific previously published works, please review and evaluate these publications to determine whether they are relevant and should be cited. There is no requirement to cite these works unless the editor has indicated otherwise.

Reviewers' comments:

Reviewer's Responses to Questions

**Comments to the Author**

1. Is the manuscript technically sound, and do the data support the conclusions?

Reviewer #1: Yes

Reviewer #2: Partly

Reviewer #3: Yes

Reviewer #4: Yes

Reviewer #5: Partly

**********

2. Has the statistical analysis been performed appropriately and rigorously?

Reviewer #1: Yes

Reviewer #2: N/A

Reviewer #3: Yes

Reviewer #4: N/A

Reviewer #5: Yes

**********

3. Have the authors made all data underlying the findings in their manuscript fully available?

The PLOS Data policy

Reviewer #1: Yes

Reviewer #2: No

Reviewer #3: No

Reviewer #4: Yes

Reviewer #5: Yes

**********

4. Is the manuscript presented in an intelligible fashion and written in standard English?

Reviewer #1: Yes

Reviewer #2: Yes

Reviewer #3: Yes

Reviewer #4: Yes

Reviewer #5: No

**********

Reviewer #1: No novelty in the present state of the manuscript.

Authors have done work on the titlte "Hybrid Machine Learning Driven Optimization of Multilayer SPR Sensor for High Sensitivity Milk Fat Detection".

There are several papers on the similar topic.

Reviewer #2: Reviewer’s Comments

Manuscript Title: Hybrid Machine Learning Driven Optimization of Multilayer SPR Sensor for High Sensitivity Milk Fat Detection

Recommendation: Major Revision

The manuscript combines SPR sensing with machine learning for fat/milk analysis, which is an interesting direction. However, in its current form, the work needs significant clarification and strengthening in terms of methodology, validation, and practical relevance.

1. The idea of combining SPR with machine learning is promising, but the novelty is not clearly established. Similar approaches using ML for optical sensing and classification have already been reported. The authors should clearly highlight what is new in their framework (e.g., dataset, model, feature extraction, or sensing strategy).

2. The sensing mechanism itself is not sufficiently explained. It is unclear how variations in milk composition (fat content or adulteration) are translated into refractive index changes and how reliably SPR captures these variations.

3. A major concern is the source of refractive index values used for milk or fat variations. The manuscript does not clearly mention whether these values are taken from experimental data, literature, or assumed. Proper references or justification are required.

4. The manuscript does not address whether similar refractive index variations could arise from other components present in milk (e.g., proteins, lactose, added water, or other adulterants). This raises concerns regarding selectivity and specificity.

5. The role of machine learning is not deeply justified. It is not clear, What exact features are used (SPR angle, reflectance curve, etc.), also Why ML is necessary instead of direct analytical modelling and further, Whether the dataset size is sufficient for reliable training

6. There is no clear discussion of dataset preparation such as How many samples were used, Whether the data is simulated or experimental, How the data is split (training/testing)

7. How sensitive is the model to noise?

8. Will small measurement errors affect classification?

9. From the SPR side, the analysis is somewhat limited. The paper would benefit if authors mention the Clear resonance curve analysis, Shift explanation with changing refractive index and Physical interpretation of sensing behavior.

10. The practical applicability is not fully convincing. It is unclear how the proposed system would work in real milk samples where multiple variables change simultaneously.

11. Manuscript requires a comaparision table also in this field with the previous relevant published data.

Reviewer #3: I am uploading my detailed review as an attachment, as it exceeds the character limit of this section.

The manuscript describes a technically sound piece of scientific research, and the data presented adequately support the conclusions drawn by the authors. However, it requires further revisions and clarifications in certain sections to improve its overall quality and rigor. Detailed comments and specific suggestions are provided in the attached review document.

Reviewer #4: The manuscript deals with the numerical analysis of the surface plasmon resonance (SPR) sensor based on the Kretschmann configuration for the measurement of fat content in milk. The authors of the contribution form a team specializing in the design and optimization of highly sensitive SPR biosensors, particularly utilizing multilayer configurations for medical diagnostics.

It's worth taking note of the issue of selecting the right prism material for measuring milk's fat content, which was ultimately determined to be SiO2. Also, a clear explanation of the mechanism of operation of the SPR sensors would be a good read.

On the other hand, I am concerned about the assumption in the simulations that the thickness of some layers changes by 0.1 nm, which corresponds to the size of an atom or smaller, and therefore is probably not wholly justified.

It is also not entirely clear to me how the calculated sensitivity of the sensor translates into the sensitivity of measuring the fat content in milk.

Finally, the authors could add a few review publications on the measurement of fat in milk and in general of its adulteration [1-3], and above all, refer to publications dealing with exactly the same problem, i.e. the measurement of fat content in milk using SPR sensors. I am referring to the publications listed in the list below [4-7].

It's happened to me a few times that authors describe sensors in Kretschmann configuration and treat them as common knowledge, without citing their author [8]. It's crucial to give credit to the author and include him in the references.

In conclusion, I would be in favor of publishing this work if the authors could respond convincingly to the above comments.

References:

1. D Maheswara Reddy, K Venkatesh, and C Venkata Sesha Reddy: “Adulteration of Milk and Its Detection: A Review,” Int. J. Chem. Stud. 5, No. 4, (2017), 613–617.

2. Siuli Das, Bhaswati Goswami, and Karabi Biswas: “Milk Adulteration and Detection: A Review,” Sens. Lett. 14, No. 1, (2016), 4–18.

3. Sneh Lata Chauhan, Priyanka, Kruti Debnath Mandal, Babul Rudra Paul, and Chinmoy Maji: “Adulteration of milk: A Review,” Int. J. Chem. Stud. 7, No. 1, (2019), 2055–2057.

4. Abdulkarem H. M.Almawgani, Malek G. Daher, Sofyan A. Taya, Mohammad Mashagbeh, and Ilhami Colak: “Optical detection of fat concentration in milk using MXene-based surface plasmon resonance structure,” Biosensors 12, No. 7, (2022), 535.

5. Bhishma Karki, Youssef Trabelsi, Arun Uniyal, Amrindra Pal, and Ram Bharos Yadav: “Detection of fat concentration milk using TMDC-based surface plasmon resonance sensor,” Mod. Phys. Lett. B 38, No. 29, (2024), 2450253.

6. Leila Fatolahi, Tamara Shamil Addulrahman, Sepideh Alemi, Mohammed N. Al-Delfi, Ayat H. Athab, and Baadal Jushi Janani: “Optical detection of fat and adulterants concentration milk using TMDC (WS2 and MoS2)-surface plasmon resonance sensor via high sensitivity and detection accuracy,” Opt. Mater. 147, (2024), 114723.

7. Emranul Haque, Abdullah Al Noman, S. M. Rakibul Islam, Kh Shahriya Zaman, and Feroz Ahmed: “Numerical Analysis of a PCF-SPR Sensor for Adulterant Detection in Milk,” 2025 Photonics Global Conference (PGC), Sanya, China, 2025, pp. 1-3.

8. E. Kretschmann and H. Raether, "Notizen, "Radiative decay of non-radiative surface plasmons excited by light," Zeitschrift Naturforschung A 23, 2135-2136, (1968).

Reviewer #5: Comments

Manuscript Title: Hybrid Machine Learning Driven Optimization of Multilayer SPR Sensor for High Sensitivity Milk Fat Detection

Recommendation: Major Revision

The manuscript presents a multilayer SPR biosensor based on SiO₂/MgO/Ag/BaTiO₃/BP configuration for milk fat detection using refractive index variation. The authors employ TMM, FEM, and FDTD analyses along with hybrid optimization involving brute-force and machine learning-assisted refinement. The topic is relevant to optical biosensing and food-quality monitoring, and the manuscript demonstrates promising sensor sensitivity and comprehensive numerical analysis. However, the manuscript in its current form requires substantial revision before it can be considered for publication.

1. How does the proposed hybrid ML optimization differ from conventional parameter sweeps? What scientific advancement beyond incremental sensitivity enhancement is achieved?

2. The title emphasizes “Hybrid Machine Learning Driven Optimization,” yet the ML methodology lacks sufficient technical detail such as: Type of ML model used, Input features and output targets, Training/testing dataset size, Data splitting strategy, Hyperparameters, Validation metrics (RMSE, MAE, R², etc.), Comparison with conventional optimization methods and Computational efficiency gains.

3. The manuscript assumes RI variation from 1.345 to 1.3621 corresponding to milk fat concentration. The authors should: provide experimental references correlating RI with fat percentage, clarify whether temperature dependence is considered, discuss compositional variability in real milk samples

4. Explain whether adulterants or proteins affect RI measurements.

5. Fig. 11 captions appear inconsistent:

“The RI of the corresponding fluoride concentration...”

This is likely an editing error because the manuscript concerns milk fat detection, not fluoride concentration. All figure captions should be revised carefully.

6. Several grammatical and stylistic issues are present throughout the manuscript. Professional English editing throughout the manuscript is highly recommended.

7. Repetitive sentence structures are observed, such as:

“This is because...” appears repeatedly in the Introduction.

8. Some references appear duplicated: References [15] and [16] seem identical.

9. Typographical issues in many instances such as:

“Polinomial Fit” should be “Polynomial Fit.”

10. The introduction discussion is poor and insufficiently structured. The literature review lacks critical analysis of recent advancements in multilayer SPR biosensors, particularly those involving hybrid dielectric materials, 2D materials, and machine learning-assisted optimization techniques. The authors are strongly advised to enrich the Introduction section using:doi.org/10.1007/s11468-025-02961-1, 10.1016/j.microc.2026.117902, doi: 10.1109/TNB.2026.3664930, doi.org/10.1007/s11468-025-02823-w.

**********

what does this mean?). If published, this will include your full peer review and any attached files.

If you choose “no”, your identity will remain anonymous but your review may still be made public.

**Do you want your identity to be public for this peer review?**  For information about this choice, including consent withdrawal, please see our Privacy Policy

Reviewer #1: **Yes:**  Dr. Chella Santhosh

Reviewer #2: No

Reviewer #3: No

Reviewer #4: No

Reviewer #5: No

**********

[NOTE: If reviewer comments were submitted as an attachment file, they will be attached to this email and accessible via the submission site. Please log into your account, locate the manuscript record, and check for the action link "View Attachments". If this link does not appear, there are no attachment files.]

To ensure your figures meet our technical requirements, please review our figure guidelines: https://journals.plos.org/plosone/s/figures

You may also use PLOS’s free figure tool, NAAS, to help you prepare publication quality figures: https://journals.plos.org/plosone/s/figures#loc-tools-for-figure-preparation.

NAAS will assess whether your figures meet our technical requirements by comparing each figure against our figure specifications.

Attachment Submitted filename: review_report_PONE-D-26-15273.pdf

# Author comment: Author response to Decision Letter 1

**DOI:** 10.1371/journal.pone.0355996.r002

3 Jul 2026

Subject: Thank You for the Revision Opportunity - Manuscript

Dear Professor Zaky A. Zaky,

I would like to sincerely thank you for your valuable guidance and for giving me the opportunity to revise my manuscript entitled “Hybrid Machine Learning Driven Optimization of Multilayer SPR Sensor for High Sensitivity Milk Fat Detection.”

I deeply appreciate the constructive feedback provided by you and the reviewers. Your comments have been instrumental in identifying key areas for improvement, including section organization, figure and table modifications, abstract and conclusion refinement, and overall presentation quality. I have carefully addressed every point raised and made the necessary revisions to enhance the manuscript’s readability, accuracy, and clarity. I also ensured that the data presentation, references, and formatting fully comply with the journal’s requirements.

Thank you once again for your and the reviewers time, effort, and thoughtful insights. The revision process has been an enriching experience, and I believe that the manuscript has significantly improved as a result of your valuable feedback.

I look forward to your further assessment of the revised version.

Sincerely,

Md. Al Amin Islam Utshob

Response to Editor

Dear Professor Zaky A. Zaky,

On behalf of all co-authors, I would like to express our sincere gratitude for your valuable time and consideration in handling our manuscript entitled “Hybrid Machine Learning Driven Optimization of Multilayer SPR Sensor for High Sensitivity Milk Fat Detection.” We are also truly grateful to the reviewers for their insightful and constructive comments, which have been invaluable in improving the clarity, rigor, and overall quality of our work.

We have carefully revised the manuscript in accordance with all the comments and suggestions provided by the reviewers and have included a detailed point-by-point response outlining the changes made. We believe that the revisions have strengthened the manuscript considerably.

We sincerely thank you for the valuable comments and suggestions, which have significantly improved the quality of our manuscript. Based on the reviewer’s and editor’s recommendations, we have thoroughly revised the manuscript. The major revisions are summarized below:

Page 1: Content from Line 23 has been revised and updated. Additionally, new text has been added in Lines 42-47.

Page 2: Lines 67-69 have been removed. New content has been incorporated in Lines 69-70, 72-77, and 92-98.

Page 3: New content has been added in Lines 99-114.

Page 4: New content has been added in Lines 181-183.

Page 7: The content from Line 245 has been revised and updated.

Page 10: New content has been added in Lines 293-298.

Page 14: New content has been added in Lines 385-404.

Page 15: Lines 405-410 have been removed, and new content has been added in Lines 425-427.

Page 16: Part of the content in Lines 445-446 has been removed, and new content has been added in Lines 438-445.

Page 17: The caption/title of Figure 11 has been revised.

Page 18: A new subsection (Section 3.7) has been introduced, and new content has been added in Lines 504-511.

Page 19: A new subsection (Section 3.8) has been added. New content has been incorporated in Lines 515-531, and a new Figure 13 has been included.

Page 20: A new row titled "Application" has been added to Table 6.

In addition, the manuscript has been carefully proofread, and grammatical, typographical, and language-related errors have been corrected throughout the manuscript to improve its clarity and readability.

As per the reviewer’s feedback, new citations have been added at 6, 7, 8, 15, 19, 26, 28, 32-39, 48, 49, 51, 53, 62, 63, 64, and 66.

To make revisions transparent for the reviewers and editor, we have applied the following color-coding in the revised manuscript:

Red text → indicates removed lines/content.

Green text → indicates newly added lines/content.

This ensures that all changes are easily traceable.

We believe these revisions significantly improve the clarity, focus, and overall quality of the manuscript. We thank you and the reviewers for your constructive feedback and look forward to your further consideration.

Response to Reviewer #2

Reviewer#2, Concern #1: The idea of combining SPR with machine learning is promising, but the novelty is not clearly established. Similar approaches using ML for optical sensing and classification have already been reported. The authors should clearly highlight what is new in their framework (e.g., dataset, model, feature extraction, or sensing strategy).

Author Response: Thank you for this important remark from the reviewer. We do agree that the novelty of the suggested machine learning hybrid method must be emphasized more clearly.

The novelty of the current study does not consist in the application of machine learning alone, because such ML-supported optical sensing was reported earlier. The innovation of this study consists in the development of a physics-guided hybrid optimization method combining the TMM, MLR, and Brute Force algorithms for designing a multilayer SPR sensor. Contrary to the existing ML-based sensing methodologies that concentrate on the classification or detection of signals using experimental datasets, the novel framework uses TMM-simulated data to train an MLR surrogate model to derive the connection between the multilayer structure parameters and the performance of the sensor. Further, the trained model is coupled with a Multidimensional Brute Force Optimization method to determine the optimum values of MgO, Ag, and BaTiO3 thicknesses. This optimization technique does not require any experimental dataset and also reduces the computational time by eliminating the need for running multiple full-wave simulations. In addition, the proposed framework is used to optimize a BP/BaTiO3 multilayer SPR sensor to detect milk refractive index sensing, achieving an enhancement in the maximum sensitivity from 401.40 deg/RIU to 424.61 deg/RIU. As a means of enhancing the contributions of the paper, we have reviewed the Introduction and Machine Learning Methodology sections.

Reviewer#2, Concern #2: The sensing mechanism itself is not sufficiently explained. It is unclear how variations in milk composition (fat content or adulteration) are translated into refractive index changes and how reliably SPR captures these variations.

Author Response: Thank you for your concern. We agree with the reviewer that there was some need for clarification regarding the sensing mechanism in the original manuscript.

The new manuscript has been extended to include an explanation of the sensing mechanism. The sensing mechanism works on the premise that changes in milk composition, such as a change in fat composition, affect the effective refractive index (RI) of the milk solution. Refractive indices used in our study were not taken arbitrarily; rather, they were taken from earlier experimental results found in literature. A change in the effective RI of the analyte leads to a change in the propagation constant of the surface plasmon wave at the metal-analyte interface, resulting in the disruption of the momentum matching condition necessary for SPR excitation. The result is a change in the resonance angle and this angular shift is measured using the angular interrogation method. In addition, in order to provide further insight into the potential path towards practical implementation, we provided an extended section discussing future work. Specifically, the proposed SPR device can be made into a selective biosensor by adding the biomarker specific biorecognition layers, like lipase, PAA-Chitosan-Lipase or PMMA-Lipase [1], [2], [3]. In the case of such an arrangement, the triglycerides, which are the main biomarkers for milk fat, will be specifically bound by the immobilized lipase layer. As a result, there will be an interfacial biochemical reaction causing an alteration of the local dielectric environment, leading to an additional refractive index change, detectable as a shift of the SPR resonance angle.

To better specify the scope of the current paper, we also included the explanation that the current device is a refractive-index based sensing platform using experimentally obtained values of refractive index of milk. The quantitative calibration between fat content, bioreactions and SPR signal obtained experimentally will be a subject of our future work. These clarifications have been added to the new manuscript version.

[1] O. Kirk and M. W. Christensen, “Lipases from Candida antarctica: Unique Biocatalysts from a Unique Origin,” Org. Process Res. Dev., vol. 6, no. 4, pp. 446–451, Jul. 2002, doi: 10.1021/op0200165.

[2] M. Chakraborty and K. Biswas, “Hardware Platform to Detect Fat Percent in Milk Using a Lipase Immobilized PMMA-Coated Sensor,” IEEE Trans. Instrum. Meas., vol. 68, no. 11, pp. 4526–4534, 2019, doi: 10.1109/TIM.2018.2890320.

[3] S. Zhou, X. Li, J. Zhang, H. Yuan, X. Hong, and Y. Chen, “Dual-fiber optic bioprobe system for triglyceride detection using surface plasmon resonance sensing and lipase-immobilized magnetic bead hydrolysis,” Biosens. Bioelectron., vol. 196, p. 113723, Jan. 2022, doi: 10.1016/J.BIOS.2021.113723.

Reviewer#2, Concern #3: A major concern is the source of refractive index values used for milk or fat variations. The manuscript does not clearly mention whether these values are taken from experimental data, literature, or assumed. Proper references or justification are required.

Author Response: We sincerely acknowledge the insightful feedback. It is important to state that all the results shown in the paper are based on computer simulation and do not involve any experimental data acquisition process. The values of the refractive index (RI) from 1.345 to 1.3621 were used in accordance with previous publications where experimental correlations between RI and composition changes in milk, including its fat content, have been established [4], [5]. Suitable literature references have been provided in the revised version of the manuscript. Additionally, it has been specified in the manuscript that variations of the RI due to the effect of temperature change were not taken into account in the present simulations since the aim was to assess the sensor intrinsic performance under controlled conditions. Moreover, it has been noted that the effect of temperature and other constituents, including proteins, lactose, and minerals in addition to fat, might also affect the RI in practical milk samples.

[4] A. J. Jääskeläinen, K. E. Peiponen, and J. A. Räty, “On reflectometric measurement of a refractive index of milk,” J. Dairy Sci., vol. 84, no. 1, pp. 38–43, 2001, doi: 10.3168/jds.S0022-0302(01)74449-9.

[5] B. Karki, Y. Trabelsi, A. Uniyal, A. Pal, and R. Bharos Yadav, “Detection of fat concentration milk using TMDC-based surface plasmon resonance sensor,” Modern Physics Letters B, vol. 38, no. 29, Oct. 2024, doi: 10.1142/S0217984924502531.

Revised text (Page 2, Line 92-98): “The detection principle behind the suggested SPR sensor is based on angular interrogation. The alterations in the milk’s components composition, such as alterations in the fat component or in its adulteration, alter the effective refractive index of the medium, as shown by experimental studies [32]. Such alterations in the effective refractive index change the momentum matching required for exciting surface plasmons in the metal-analyte interface, thereby causing an alteration in the SPR resonance angle. Consequently, the suggested sensor indirectly senses any alterations in milk’s components composition through the alteration in its refractive index.”

Reviewer#2, Concern #4: The manuscript does not address whether similar refractive index variations could arise from other components present in milk (e.g., proteins, lactose, added water, or other adulterants). This raises concerns regarding selectivity and specificity.

Author Response: We are truly grateful to the reviewer for this important comment. In our opinion, the bulk refractive index change in milk could not occur only due to the change in fat concentration in milk but may also be a result of some other ingredients such as protein, lactose, added water, etc. Hence, a simple SPR sensor based on refractive index measurement is not able to identify the cause of the refractive index change.

The main aim of the present study is the development and optimization of an SPR refractive index sensing model based on experimentally measured refractive index values for different concentrations of milk fat. In order to overcome this problem, the revised manuscript now clearly indicates that this proposed sensor can currently be considered as a refractive index sensing system rather than a specific milk fat biosensor. Also, the future work section has been updated to indicate that the sensing specificity can be greatly improved by using a biorecognition layer for a specific biomarker. Immobilization of lipase, PAA-Chitosan-Lipase or PMMA-Lipase functional layers which can selectively identify and catalyze the hydrolysis of triglycerides, which is the primary biomarker of milk fat, will produce localized refractive index change in response to the sensing of fat, thereby distinguishing from the refractive index change caused by other milk compounds or adulterants. Also, future experimental work will involve evaluation of the performance of this sensor when used with real milk samples having various compositions along with adulterants.

Reviewer#2, Concern #5: The role of machine learning is not deeply justified. It is not clear, What exact features are used (SPR angle, reflectance curve, etc.), also Why ML is necessary instead of direct analytical modelling and further, Whether the dataset size is sufficient for reliable training.

Author Response: Thank you very much for the valuable comments. In our opinion, the role of the machine learning (ML) framework in the manuscript could be explained in more detail.

Now, in the revised version of the manuscript, it is clearly mentioned that the ML framework does not replace the Transfer Matrix Method (TMM) framework, which is used as the physical-based forward model to calculate the optical properties of the multilayer surface plasmon resonance (SPR) sensors, whereas the ML framework is used as the surrogate modeling tool in order to explore the multidimensional design space during optimization. The simulation data set for the training of the ML model has been generated fully with the help of the TMM framework. The input variables to the ML model are the structural parameters of the sensor, i.e., thicknesses of the MgO, Ag, and BaTiO3 layers. The output or the target variables are the values of the performance parameters of the sensors, i.e., sensitivity, Rmin, FWHM, Q-factor, DA, and FOM. Motivation behind the use of ML is the increase in computational complexity in case of the optimization directly based on the simulation by TMM. In spite of the fact that TMM predicts the optical response precisely, many computations of thousands of designs become more difficult in such case. After the development of ML model predicting the performance of sensors for each combination of layers thicknesses, a multidimensional Brute Force search is used to obtain the optimal combination of layer thicknesses. Thus, the use of ML increases computational efficiency of the optimization process whereas the design of sensor itself is based on the physical TMM simulations.

In regards to the number of data samples in the dataset, 726 samples have been generated by TMM, out of which 80% was used for the training of the model, and 20% for the testing. The adopted algorithm, Multiple Linear Regression (MLR) does not require big number of data samples due to its relative simplicity and few numbers of input features. For this reason, the dataset of this size is enough to establish the dependencies between structural parameters and sensing properties. The regression equations and Mean Square Error (MSE) values are provided.

Revised text (Page 14, Line 385-404): “For angular interrogation SPR sensors, an intensive resonance dip is critical for de

Attachment Submitted filename: Response Letter.pdf

# Peer review: Decision Letter 1

**DOI:** 10.1371/journal.pone.0355996.r003

**Authors:** Zaky Zaky

28 Jul 2026

Hybrid Machine Learning Driven Optimization of Multilayer SPR Sensor for High Sensitivity Milk Fat Detection

PONE-D-26-15273R1

Dear Dr. Utshob,

We’re pleased to inform you that your manuscript has been judged scientifically suitable for publication and will be formally accepted for publication once it meets all outstanding technical requirements.

Within one week, you’ll receive an e-mail detailing the required amendments. When these have been addressed, you’ll receive a formal acceptance letter and your manuscript will be scheduled for publication.

An invoice will be generated when your article is formally accepted. Please note, if your institution has a publishing partnership with PLOS and your article meets the relevant criteria, all or part of your publication costs will be covered. Please make sure your user information is up-to-date by logging into Editorial Manager at Editorial Manager® and clicking the ‘Update My Information' link at the top of the page. For questions related to billing, please contact billing support.

If your institution or institutions have a press office, please notify them about your upcoming paper to help maximize its impact. If they’ll be preparing press materials, please inform our press team as soon as possible -- no later than 48 hours after receiving the formal acceptance. Your manuscript will remain under strict press embargo until 2 pm Eastern Time on the date of publication. For more information, please contact onepress@plos.org.

Kind regards,

Zaky A. Zaky, Ph.D.

Academic Editor

PLOS One

Additional Editor Comments (optional):

Reviewers' comments:

Reviewer's Responses to Questions

**Comments to the Author**

Reviewer #1: All comments have been addressed

Reviewer #2: All comments have been addressed

Reviewer #4: All comments have been addressed

Reviewer #5: All comments have been addressed

**********

2. Is the manuscript technically sound, and do the data support the conclusions?

Reviewer #1: Yes

Reviewer #2: Yes

Reviewer #4: Yes

Reviewer #5: Yes

**********

3. Has the statistical analysis been performed appropriately and rigorously?

Reviewer #1: Yes

Reviewer #2: Yes

Reviewer #4: N/A

Reviewer #5: Yes

**********

4. Have the authors made all data underlying the findings in their manuscript fully available?

The PLOS Data policy

Reviewer #1: Yes

Reviewer #2: Yes

Reviewer #4: Yes

Reviewer #5: Yes

**********

5. Is the manuscript presented in an intelligible fashion and written in standard English?

Reviewer #1: Yes

Reviewer #2: Yes

Reviewer #4: Yes

Reviewer #5: Yes

**********

Reviewer #1: The response given by the authors are satisfactory and can go for the accpetance of the publication.

Reviewer #2: Authors have incorporated all the comments and suggestions in the revised manuscript. on the basis of that, the manuscript can be accepted for publication.

Reviewer #4: First and foremost, it should be appreciated that the authors put a lot of effort into preparing a revised version of their manuscript. Honestly, this is the first time I have encountered a situation where a manuscript after corrections and responses to reviewers' comments is almost four times larger than its first version.

My comments were fully considered by the authors, and I no longer have any reservations about the content of the new version of the paper. Some overly far-reaching statements have been corrected or limited. I am thinking here in particular of the authors' clear statement in response to the second reviewer that “… the revised manuscript now clearly indicates that this proposed sensor can currently be considered as a refractive index sensing system rather than a specific milk fat biosensor” and to me “It is important to note that the suggested SPR sensor does not detect the concentration of milk fat, instead it detects changes in the effective refractive index (RI) of the milk sample”.

Concluding, in my opinion the manuscript in its current form can be accepted for publication in PLOS One.

Reviewer #5: (No Response)

**********

what does this mean?). If published, this will include your full peer review and any attached files.

If you choose “no”, your identity will remain anonymous but your review may still be made public.

**Do you want your identity to be public for this peer review?**  For information about this choice, including consent withdrawal, please see our Privacy Policy

Reviewer #1: **Yes:**  Dr. Chella Santhosh

Reviewer #2: No

Reviewer #4: No

Reviewer #5: No

**********

# Editor report: Acceptance letter

**DOI:** 10.1371/journal.pone.0355996.r004

**Authors:** Zaky Zaky

PONE-D-26-15273R1

PLOS One

Dear Dr. Utshob,

I'm pleased to inform you that your manuscript has been deemed suitable for publication in PLOS One. Congratulations! Your manuscript is now being handed over to our production team.

At this stage, our production department will prepare your paper for publication. This includes ensuring the following:

* All references, tables, and figures are properly cited

* All relevant supporting information is included in the manuscript submission,

* There are no issues that prevent the paper from being properly typeset

You will receive further instructions from the production team, including instructions on how to review your proof when it is ready. Please keep in mind that we are working through a large volume of accepted articles, so please give us a few days to review your paper and let you know the next and final steps.

Lastly, if your institution or institutions have a press office, please let them know about your upcoming paper now to help maximize its impact. If they'll be preparing press materials, please inform our press team within the next 48 hours. Your manuscript will remain under strict press embargo until 2 pm Eastern Time on the date of publication. For more information, please contact onepress@plos.org.

You will receive an invoice from PLOS for your publication fee after your manuscript has reached the completed accept phase. If you receive an email requesting payment before acceptance or for any other service, this may be a phishing scheme. Learn how to identify phishing emails and protect your accounts at https://explore.plos.org/phishing.

If we can help with anything else, please email us at customercare@plos.org.

Thank you for submitting your work to PLOS One and supporting open access.

Kind regards,

PLOS ONE Editorial Office Staff

on behalf of

Dr. Zaky A. Zaky

Academic Editor

PLOS One
